# Supplementary material for: A Humanized Yeast Phenomic Model of Deoxycytidine Kinase to Predict Genetic Buffering of Nucleoside Analog Cytotoxicity
Source: Genes (Basel). 2019 Sep 30;10(10):770. doi: 10.3390/genes10100770 (PMC6826991; doi:10.3390/genes10100770)
Supplement: Supplementary file 1 [file genes-10-00770-s001.zip › Additional_Files/Additional_File7_GOTermHeatmaps.zip!/Additional_File7_GOTermHeatmaps/RNA_processing.pdf]

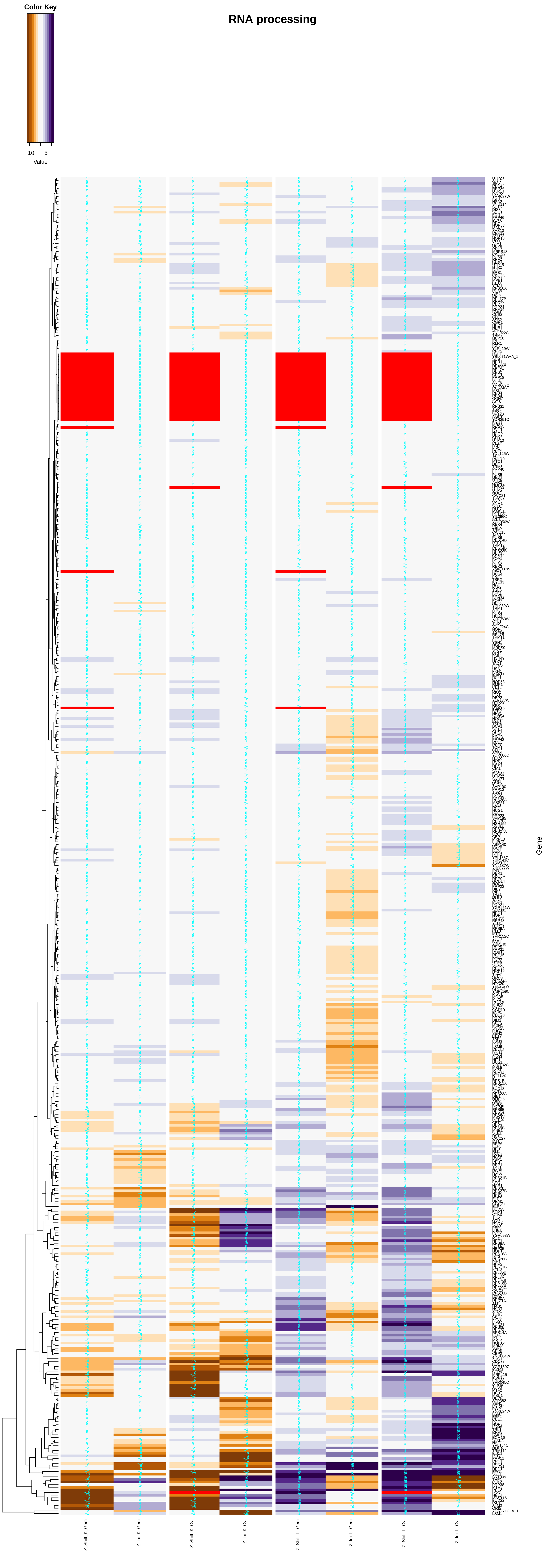

Color Key
−10
5
Value
RNA processing
Gene
Z_Shift_K_Gem
Z_Im_K_Gem
Z_Shift_K_Cyt
Z_Im_K_Cyt
Z_Shift_L_Gem
Z_Im_L_Gem
Z_Shift_L_Cyt
Z_Im_L_Cyt

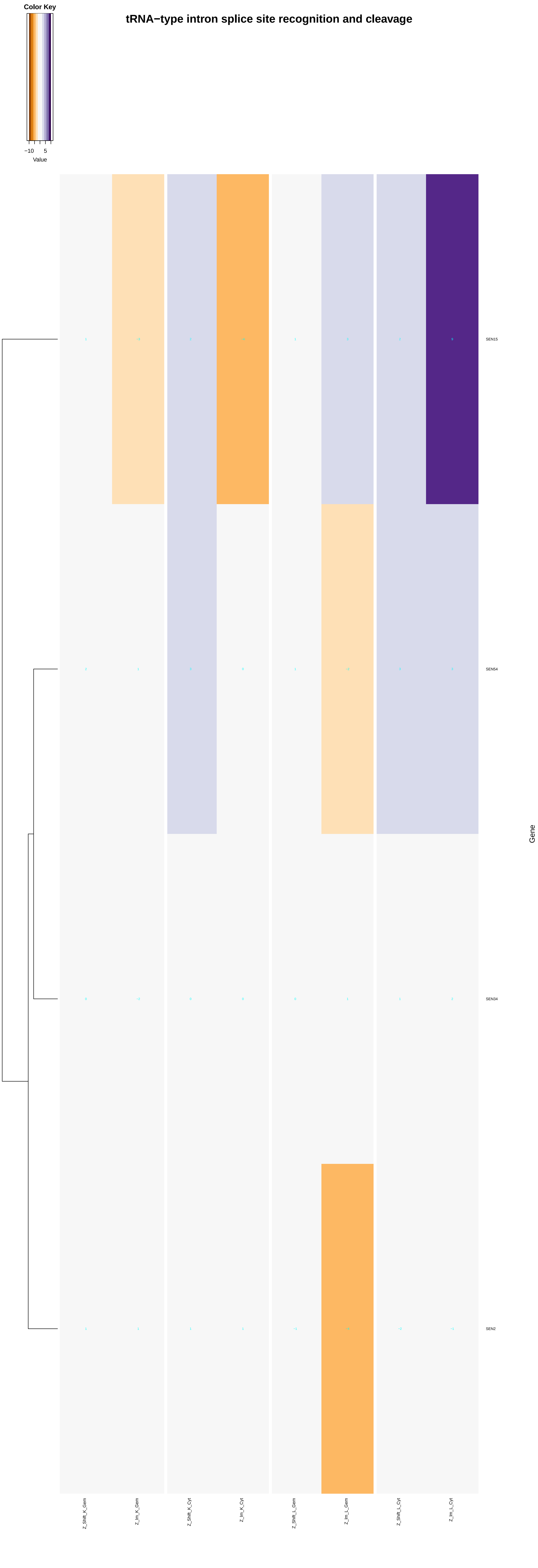
tRNA−type intron splice site recognition and cleavage
Color Key
−10
5
Value
SEN15
SEN54
SEN34
SEN2
Gene
Z_Shift_K_Gem
Z_Im_K_Gem
Z_Shift_K_Cyt
Z_Im_K_Cyt
Z_Shift_L_Gem
Z_Im_L_Gem
Z_Shift_L_Cyt
Z_Im_L_Cyt

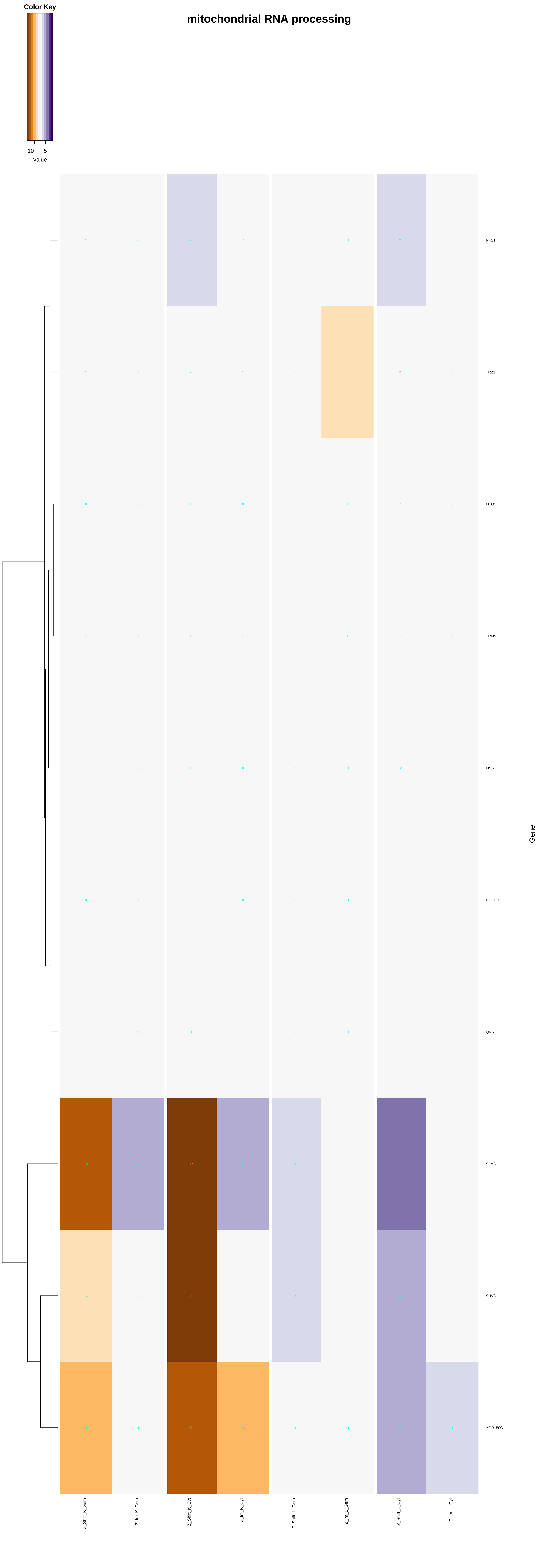
mitochondrial RNA processing
Color Key
−10
5
Value
NFS1
TRZ1
MTO1
TRM5
MSS1
PET127
QRI7
SLM3
SUV3
YGR150C
Gene
Z_Shift_K_Gem
Z_Im_K_Gem
Z_Shift_K_Cyt
Z_Im_K_Cyt
Z_Shift_L_Gem
Z_Im_L_Gem
Z_Shift_L_Cyt
Z_Im_L_Cyt

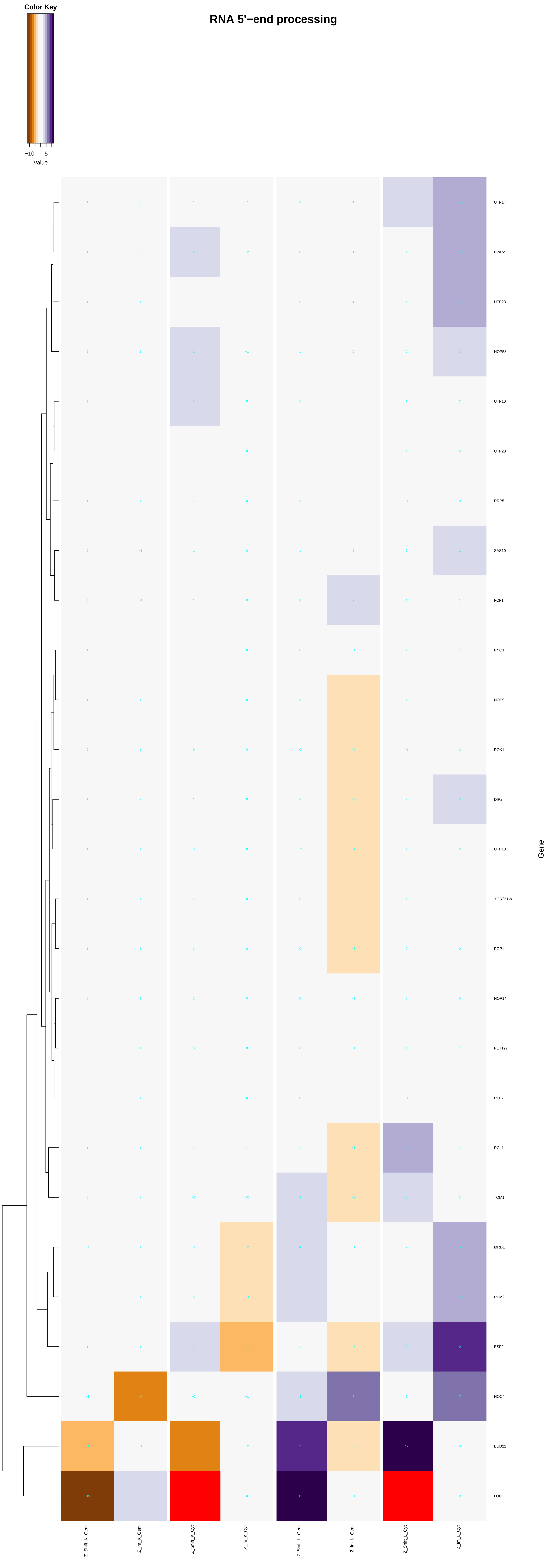
RNA 5'−end processing
Color Key
−10
5
Value
UTP14
PWP2
UTP23
NOP58
UTP10
UTP20
RRP5
SAS10
FCF1
PNO1
NOP9
ROK1
DIP2
UTP13
YGR251W
POP1
NOP14
PET127
RLP7
RCL1
TOM1
MRD1
RPM2
ESF2
NOC4
BUD21
LOC1
Gene
Z_Shift_K_Gem
Z_Im_K_Gem
Z_Shift_K_Cyt
Z_Im_K_Cyt
Z_Shift_L_Gem
Z_Im_L_Gem
Z_Shift_L_Cyt
Z_Im_L_Cyt

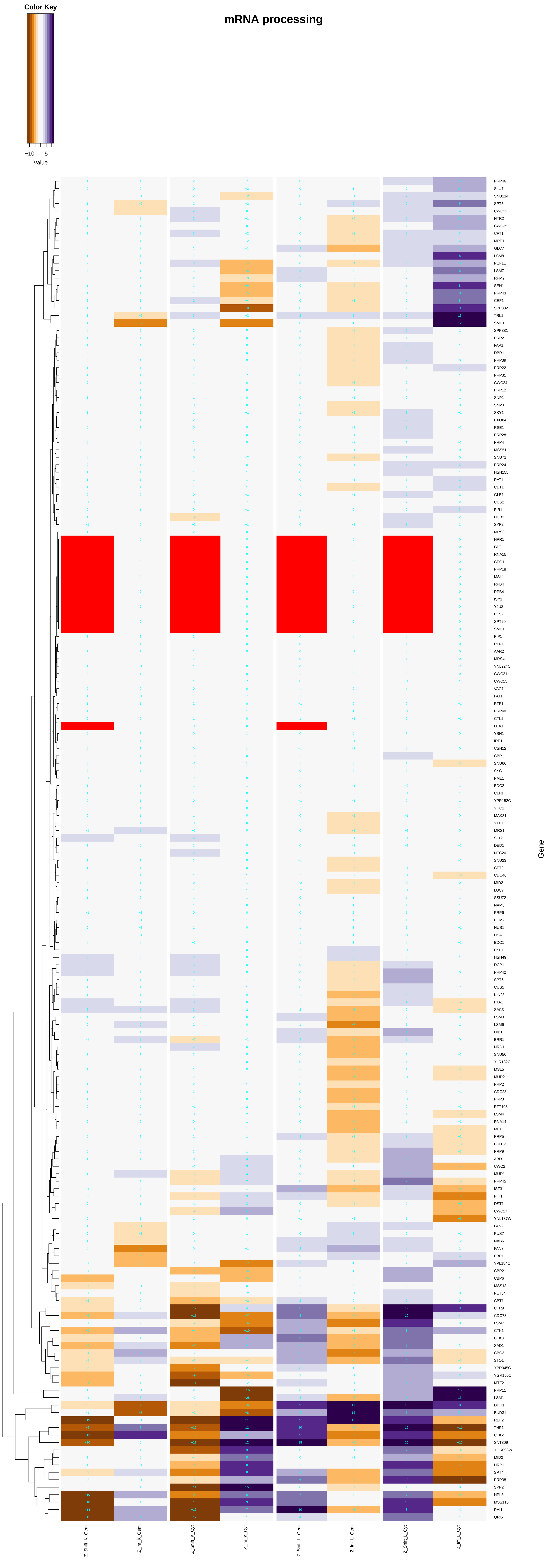
Color Key
−10
5
Value
mRNA processing
PRP46
SLU7
SNU114
SPT5
CWC22
NTR2
CWC25
CFT1
MPE1
GLC7
LSM8
PCF11
LSM7
RPM2
SEN1
PRP43
CEF1
SPP382
TRL1
SMD1
SPP381
PRP21
PAP1
DBR1
PRP39
PRP22
PRP31
CWC24
PRP12
SNP1
SNM1
SKY1
EXO84
RSE1
PRP28
PRP4
MSS51
SNU71
PRP24
HSH155
RAT1
CET1
GLE1
CUS2
FIR1
HUB1
SYF2
MRS3
HPR1
PAF1
RNA15
CEG1
PRP18
MSL1
RPB4
RPB4
ISY1
YJU2
PFS2
SPT20
SME1
FIP1
RLR1
AAR2
MRS4
YNL224C
CWC21
CWC15
VAC7
PAT1
RTF1
PRP40
CTL1
LEA1
YSH1
IRE1
CSN12
CBP1
SNU66
SYC1
PML1
EDC2
CLF1
YPR152C
YHC1
MAK31
YTH1
MRS1
SLT2
DED1
NTC20
SNU23
CFT2
CDC40
MID2
LUC7
SSU72
NAM8
PRP6
ECM2
HUS1
USA1
EDC1
FKH1
HSH49
DCP1
PRP42
SPP6
CUS1
KIN28
PTA1
SAC3
LSM3
LSM6
DIB1
BRR1
NRD1
SNU56
YLR132C
MSL5
MUD2
PRP2
CDC28
PRP3
RTT103
LSM4
RNA14
MFT1
PRP5
BUD13
PRP9
ABD1
CWC2
MUD1
PRP45
IST3
PIH1
DST1
CWC27
YNL187W
PAN2
PUS7
NAB6
PAN3
PBP1
YPL184C
CBP2
CBP6
MSS18
PET54
CBT1
CTR9
CDC73
LSM7
CTK1
CTK3
SAD1
CBC2
STO1
YPR045C
YGR150C
MTF2
PRP11
LSM1
DHH1
BUD31
REF2
THP1
CTK2
SNT309
YGR093W
MID2
HRP1
SPT4
PRP38
SPP2
NPL3
MSS116
RAI1
QRI5
Gene
Z_Shift_K_Gem
Z_Im_K_Gem
Z_Shift_K_Cyt
Z_Im_K_Cyt
Z_Shift_L_Gem
Z_Im_L_Gem
Z_Shift_L_Cyt
Z_Im_L_Cyt

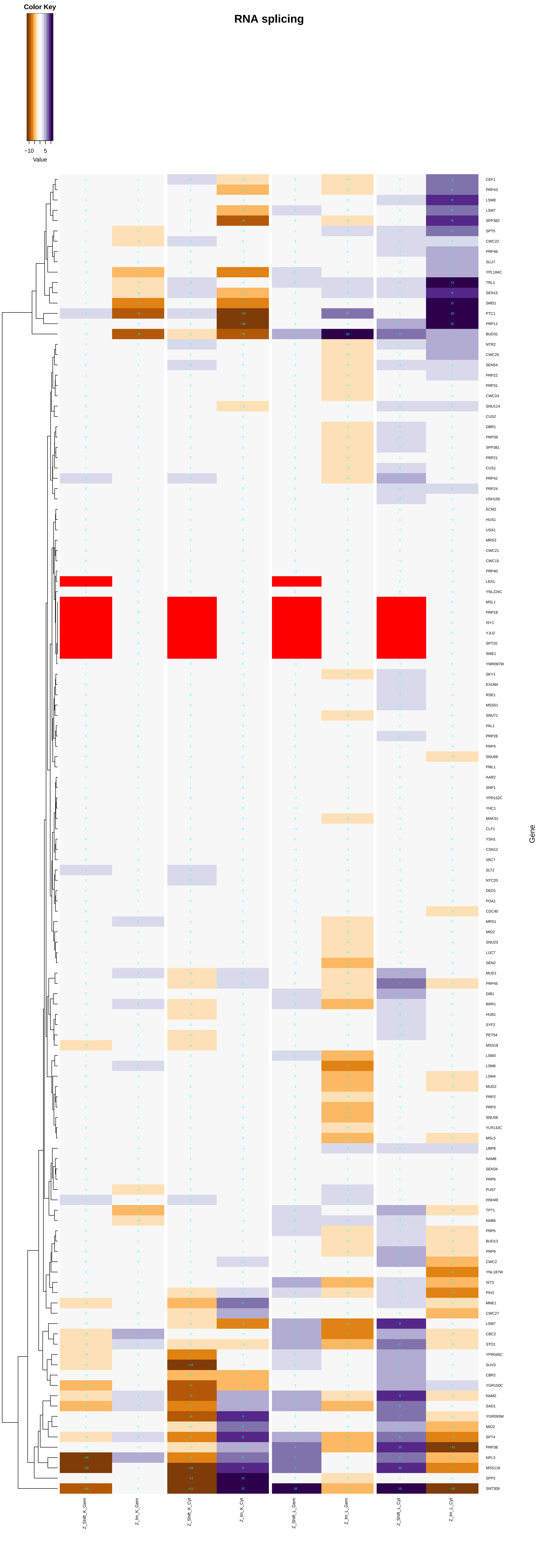

RNA splicing
Color Key
−10
5
Value
CEF1
PRP43
LSM8
LSM7
SPP382
SPT5
CWC22
PRP46
SLU7
YPL184C
TRL1
SEN15
SMD1
PTC1
PRP11
BUD31
NTR2
CWC25
SEN54
PRP22
PRP31
CWC24
SNU114
CUS2
DBR1
PRP39
SPP381
PRP21
CUS1
PRP42
PRP24
HSH155
ECM2
HUS1
USA1
MRS3
CWC21
CWC15
PRP40
LEA1
YNL224C
MSL1
PRP18
ISY1
YJU2
SPT20
SME1
YMR087W
SKY1
EXO84
RSE1
MSS51
SNU71
FAL1
PRP28
PRP4
SNU66
PML1
AAR2
SNP1
YPR152C
YHC1
MAK31
CLF1
YSH1
CSN12
VAC7
SLT2
NTC20
DED1
POA1
CDC40
MRS1
MID2
SNU23
LUC7
SEN2
MUD1
PRP45
DIB1
BRR1
HUB1
SYF2
PET54
MSS18
LSM3
LSM6
LSM4
MUD2
PRP2
PRP3
SNU56
YLR132C
MSL5
UBP8
NAM8
SEN34
PRP6
PUS7
HSH49
TPT1
NAB6
PRP5
BUD13
PRP9
CWC2
YNL187W
IST3
PIH1
MNE1
CWC27
LSM7
CBC2
STO1
YPR045C
SUV3
CBP2
YGR150C
NAM2
SAD1
YGR093W
MID2
SPT4
PRP38
NPL3
MSS116
SPP2
SNT309
Gene
Z_Shift_K_Gem
Z_Im_K_Gem
Z_Shift_K_Cyt
Z_Im_K_Cyt
Z_Shift_L_Gem
Z_Im_L_Gem
Z_Shift_L_Cyt
Z_Im_L_Cyt

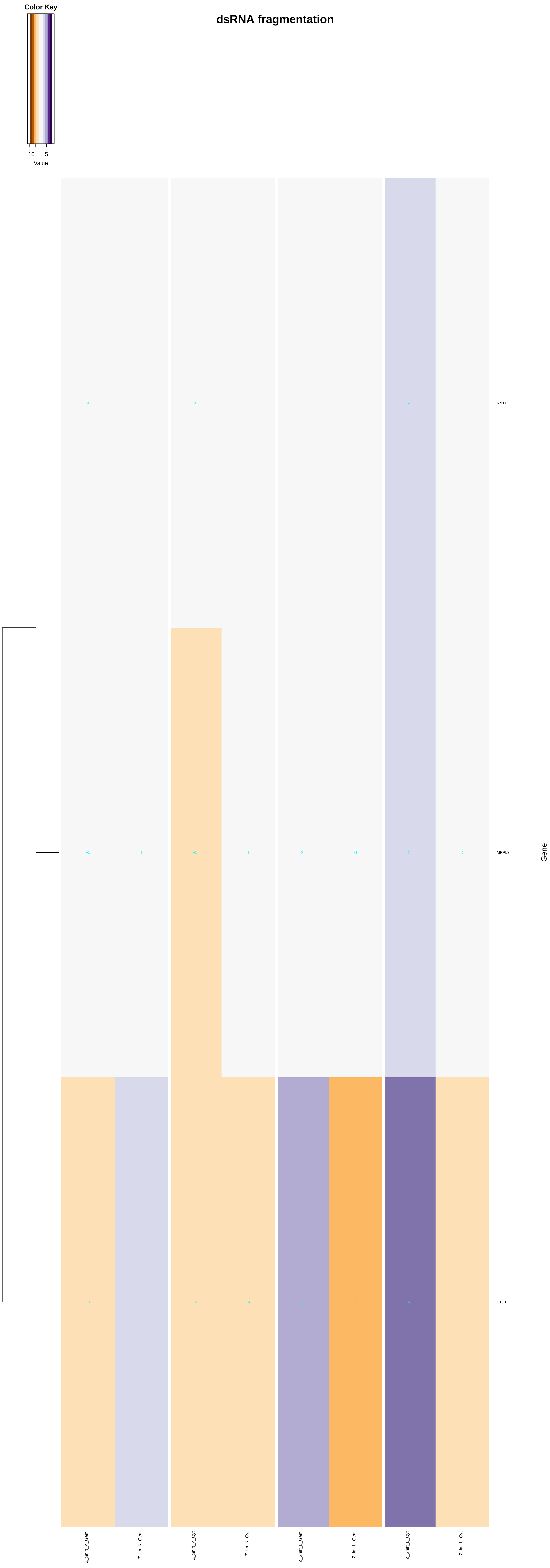
dsRNA fragmentation
Color Key
−10
5
Value
0
0
0
0
1
0
4
2
RNT1
−1
1
−2
1
0
−2
3
0
MRPL3
−3
3
−2
−4
5
−6
6
−3
STO1
Gene
Z_Shift_K_Gem
Z_Im_K_Gem
Z_Shift_K_Cyt
Z_Im_K_Cyt
Z_Shift_L_Gem
Z_Im_L_Gem
Z_Shift_L_Cyt
Z_Im_L_Cyt

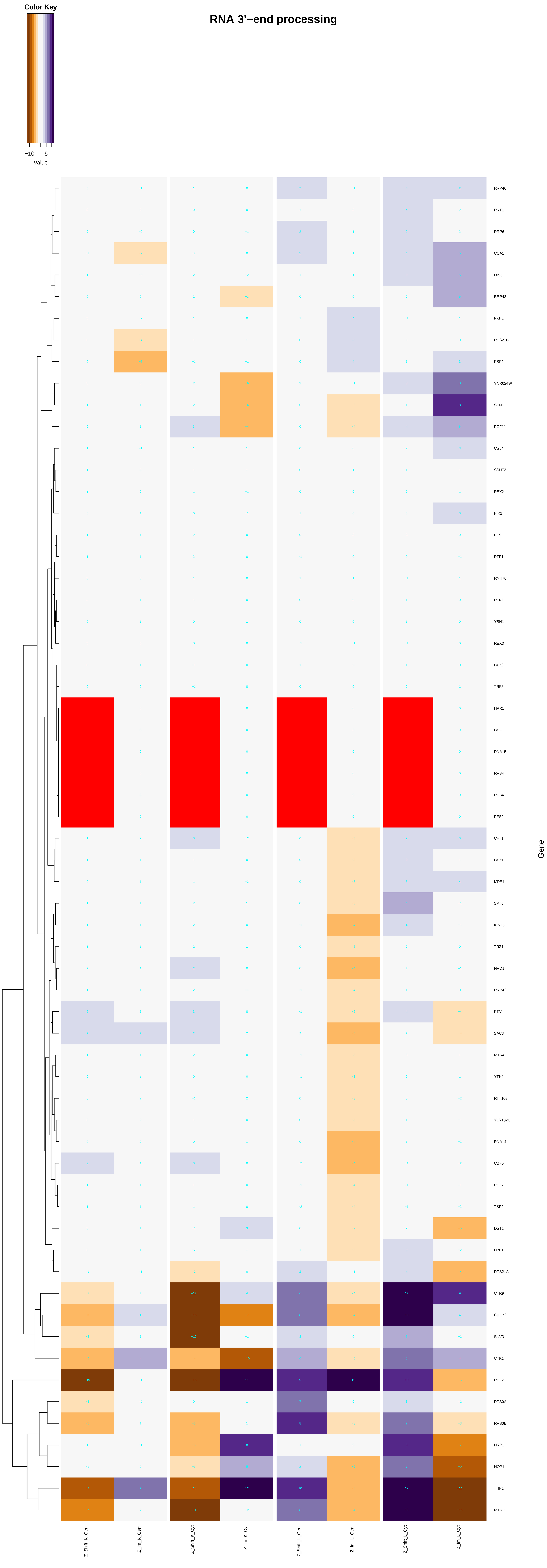

Color Key
−10
5
Value
RNA 3'−end processing
RRP46
RNT1
RRP6
CCA1
DIS3
RRP42
FKH1
RPS21B
PBP1
YNR024W
SEN1
PCF11
CSL4
SSU72
REX2
FIR1
FIP1
RTF1
RNH70
RLR1
YSH1
REX3
PAP2
TRF5
HPR1
PAF1
RNA15
RPB4
RPB4
PFS2
CFT1
PAP1
MPE1
SPT6
KIN28
TRZ1
NRD1
RRP43
PTA1
SAC3
MTR4
YTH1
RTT103
YLR132C
RNA14
CBF5
CFT2
TSR1
DST1
LRP1
RPS21A
CTR9
CDC73
SUV3
CTK1
REF2
RPS0A
RPS0B
HRP1
NOP1
THP1
MTR3
Z_Shift_K_Gem
Z_Im_K_Gem
Z_Shift_K_Cyt
Z_Im_K_Cyt
Z_Shift_L_Gem
Z_Im_L_Gem
Z_Shift_L_Cyt
Z_Im_L_Cyt
Gene

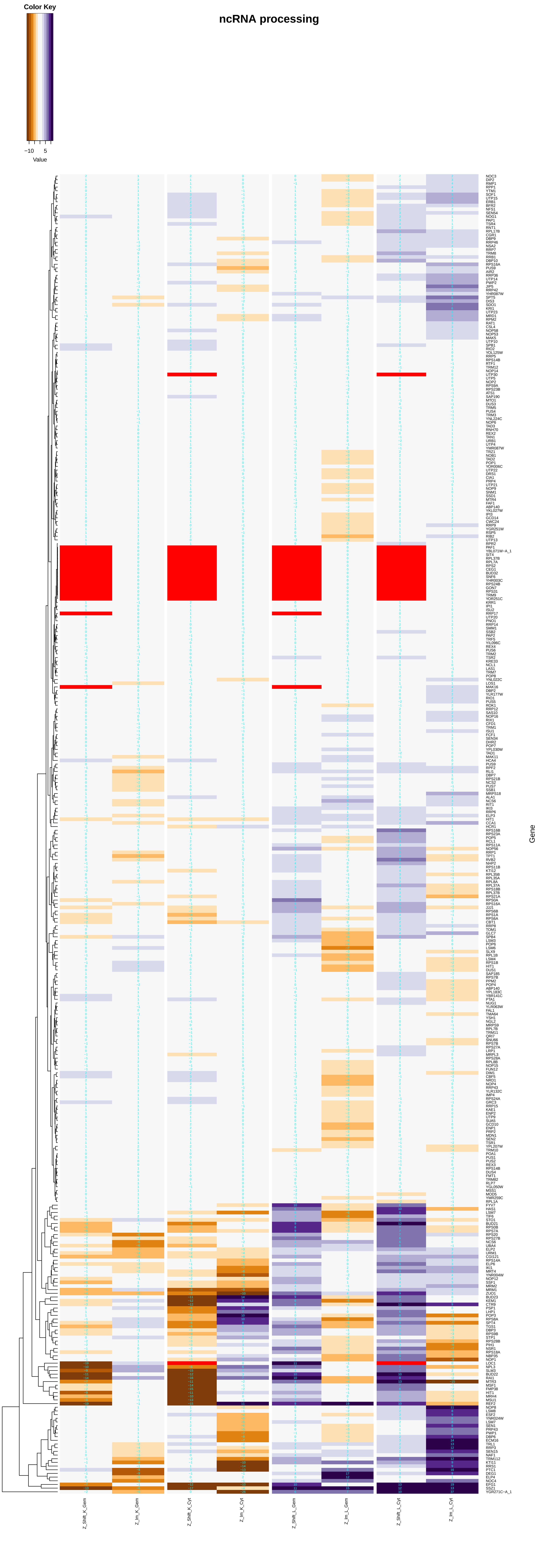

Color Key
−10
5
Value
ncRNA processing
Gene
Z_Shift_K_Gem
Z_Im_K_Gem
Z_Shift_K_Cyt
Z_Im_K_Cyt
Z_Shift_L_Gem
Z_Im_L_Gem
Z_Shift_L_Cyt
Z_Im_L_Cyt

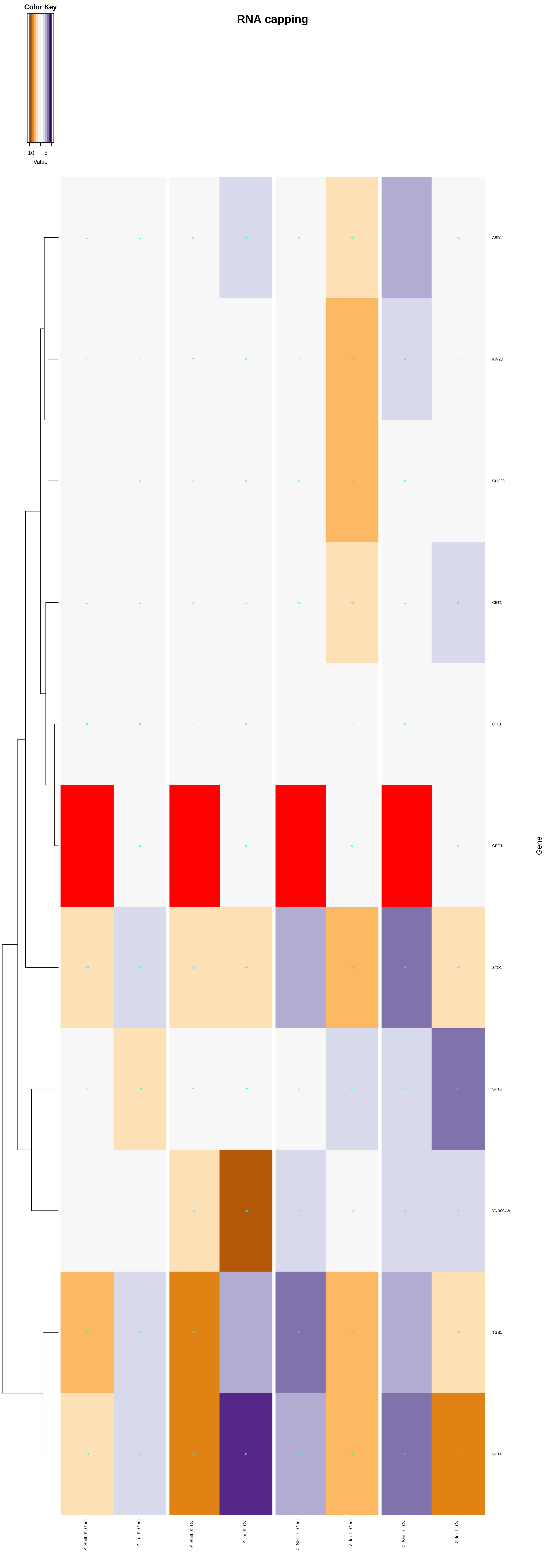
Color Key
−10
5
Value
RNA capping
ABD1
KIN28
CDC28
CET1
CTL1
CEG1
STO1
SPT5
YNR004W
TGS1
SPT4
Gene
Z_Shift_K_Gem
Z_Im_K_Gem
Z_Shift_K_Cyt
Z_Im_K_Cyt
Z_Shift_L_Gem
Z_Im_L_Gem
Z_Shift_L_Cyt
Z_Im_L_Cyt

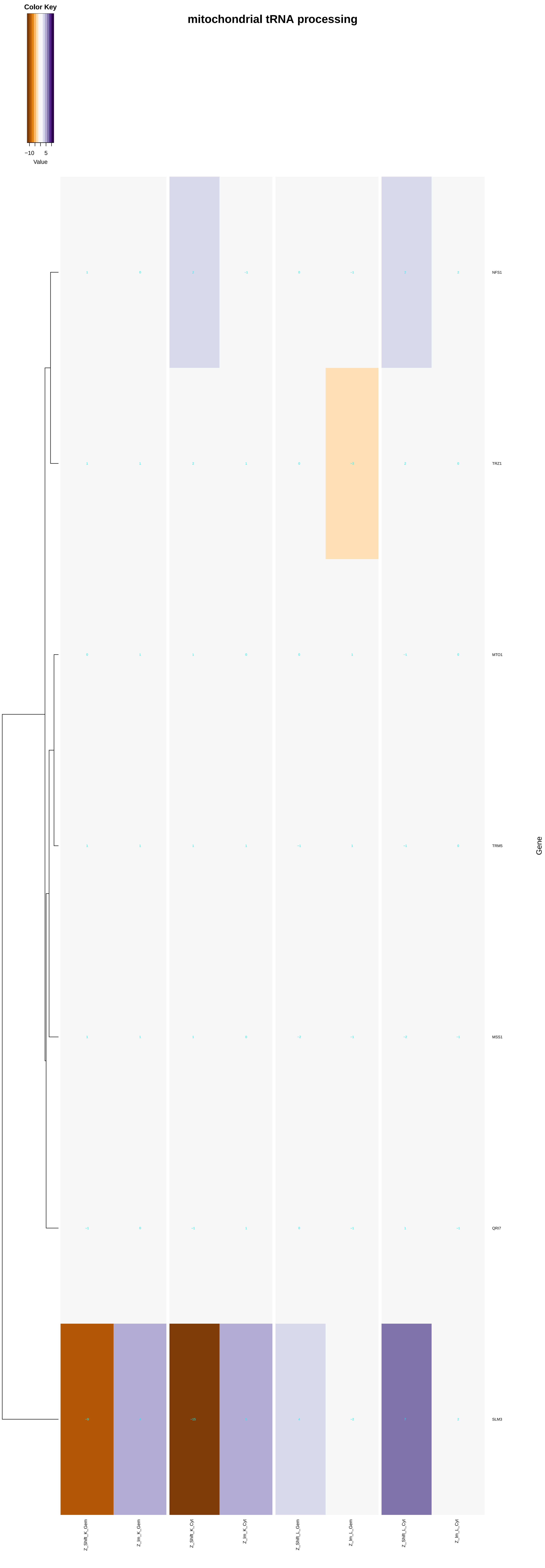
mitochondrial tRNA processing
Color Key
−10
5
Value
NFS1
TRZ1
MTO1
TRM5
MSS1
QRI7
SLM3
Gene
Z_Shift_K_Gem
Z_Im_K_Gem
Z_Shift_K_Cyt
Z_Im_K_Cyt
Z_Shift_L_Gem
Z_Im_L_Gem
Z_Shift_L_Cyt
Z_Im_L_Cyt

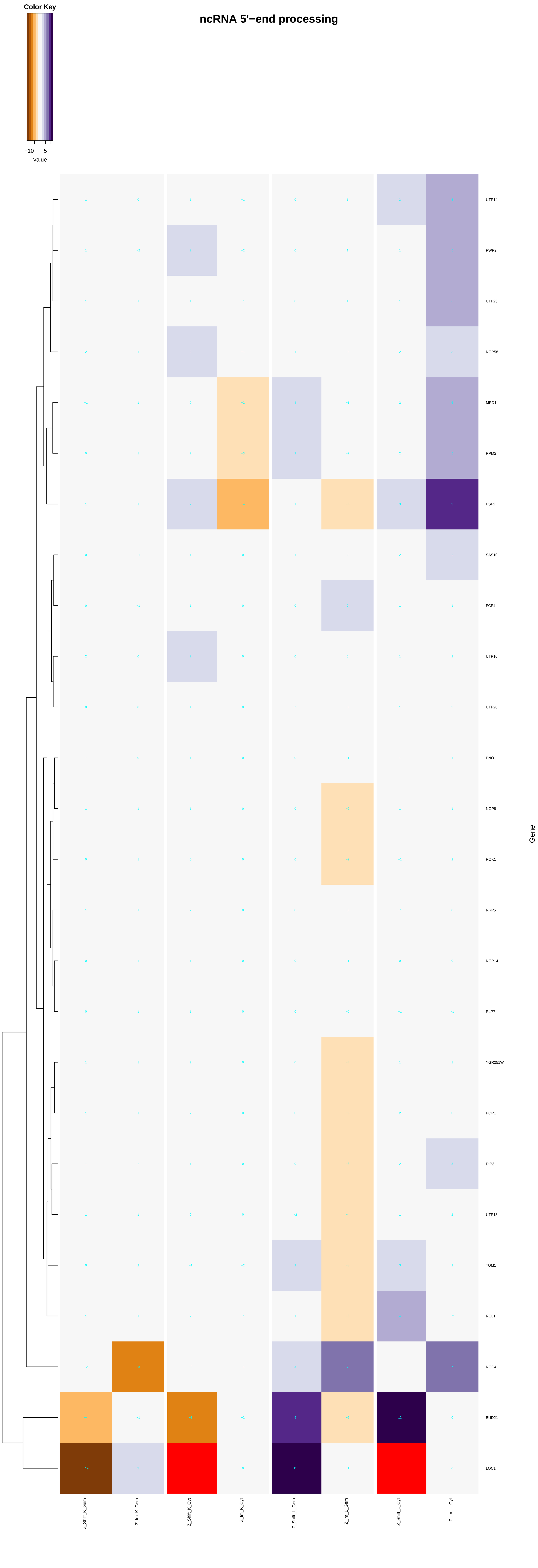

ncRNA 5'−end processing
Color Key
−10
5
Value
UTP14
PWP2
UTP23
NOP58
MRD1
RPM2
ESF2
SAS10
FCF1
UTP10
UTP20
PNO1
NOP9
ROK1
RRP5
NOP14
RLP7
YGR251W
POP1
DIP2
UTP13
TOM1
RCL1
NOC4
BUD21
LOC1
Gene
Z_Shift_K_Gem
Z_lm_K_Gem
Z_Shift_K_Cyt
Z_lm_K_Cyt
Z_Shift_L_Gem
Z_lm_L_Gem
Z_Shift_L_Cyt
Z_lm_L_Cyt

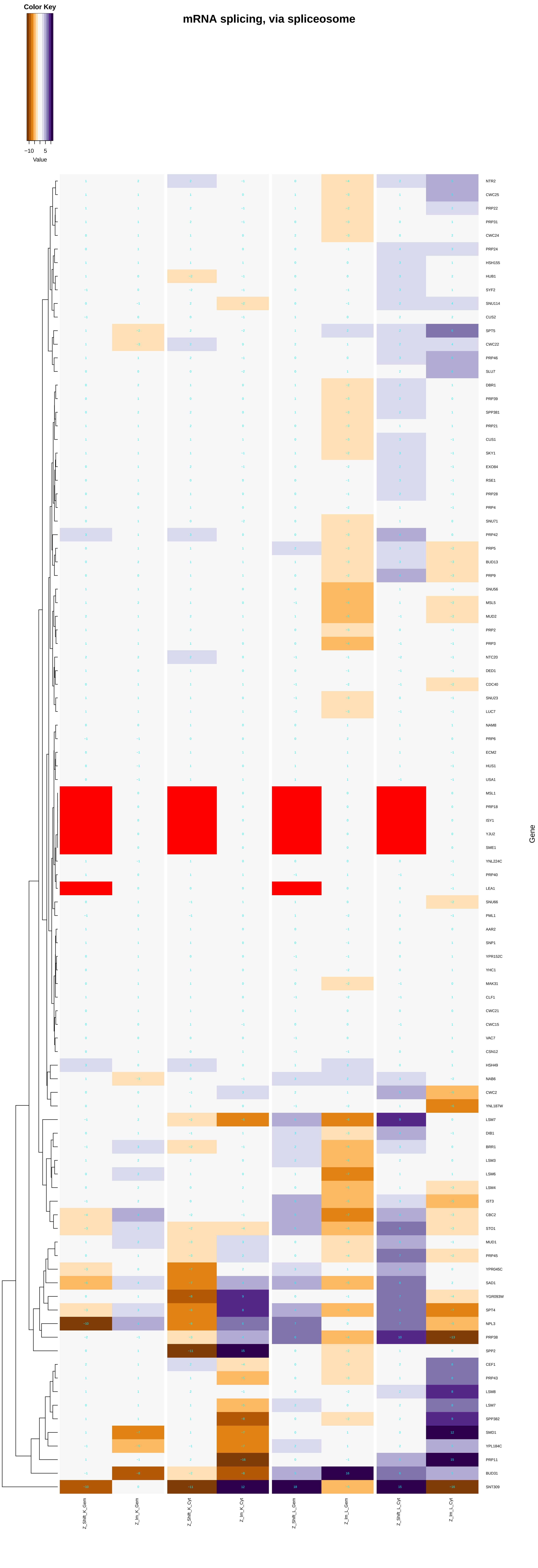

mRNA splicing, via spliceosome
Color Key
−10 5
Value
NTR2
CWC25
PRP22
PRP31
CWC24
PRP24
HSH155
HUB1
SYF2
SNU114
CUS2
SPT5
CWC22
PRP46
SLU7
DBR1
PRP39
SPP381
PRP21
CUS1
SKY1
EXO84
RSE1
PRP28
PRP4
SNU71
PRP42
PRP5
BUD13
PRP9
SNU56
MSL5
MUD2
PRP2
PRP3
NTC20
DED1
CDC40
SNU23
LUC7
NAM8
PRP6
ECM2
HUS1
USA1
MSL1
PRP18
ISY1
YJU2
SME1
YNL224C
PRP40
LEA1
SNU66
PML1
AAR2
SNP1
YPR152C
YHC1
MAK31
CLF1
CWC21
CWC15
VAC7
CSN12
HSH49
NAB6
CWC2
YNL187W
LSM7
DIB1
BRR1
LSM3
LSM6
LSM4
IST3
CBC2
STO1
MUD1
PRP45
YPR045C
SAD1
YGR093W
SPT4
NPL3
PRP38
SPP2
CEF1
PRP43
LSM8
LSM7
SPP382
SMD1
YPL184C
PRP11
BUD31
SNT309
Gene
Z_Shift_K_Gem
Z_Im_K_Gem
Z_Shift_K_Cyt
Z_Im_K_Cyt
Z_Shift_L_Gem
Z_Im_L_Gem
Z_Shift_L_Cyt
Z_Im_L_Cyt

7−methylguanosine mRNA capping

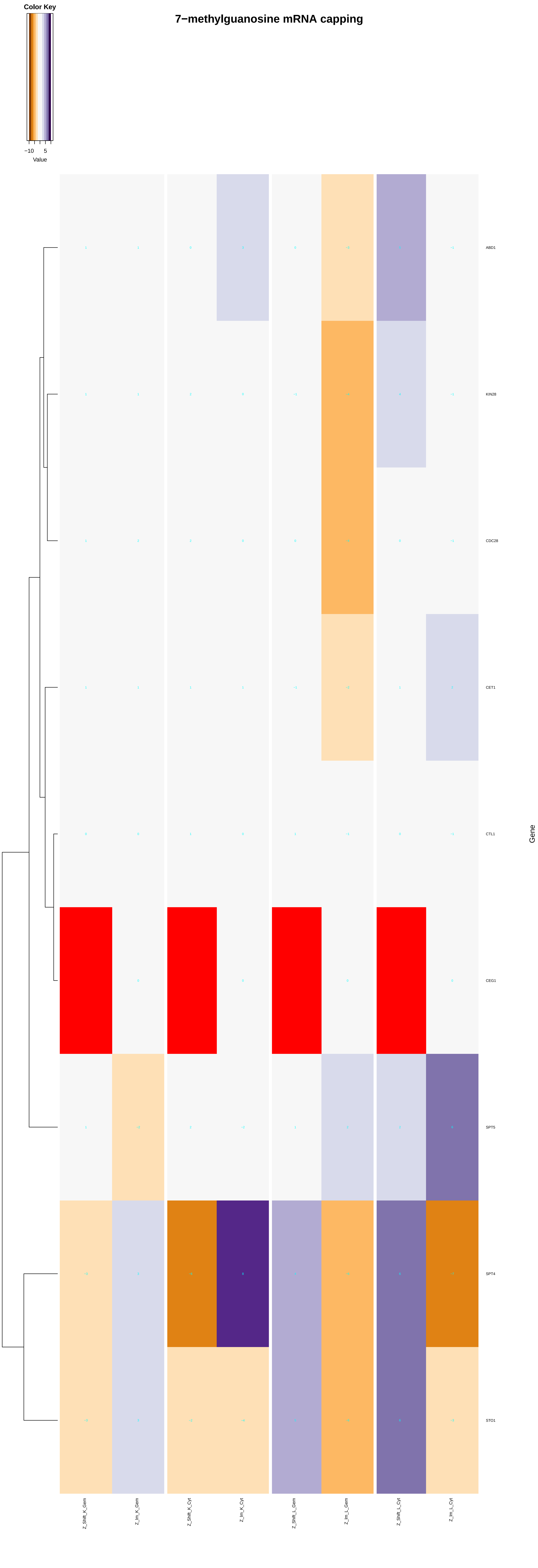

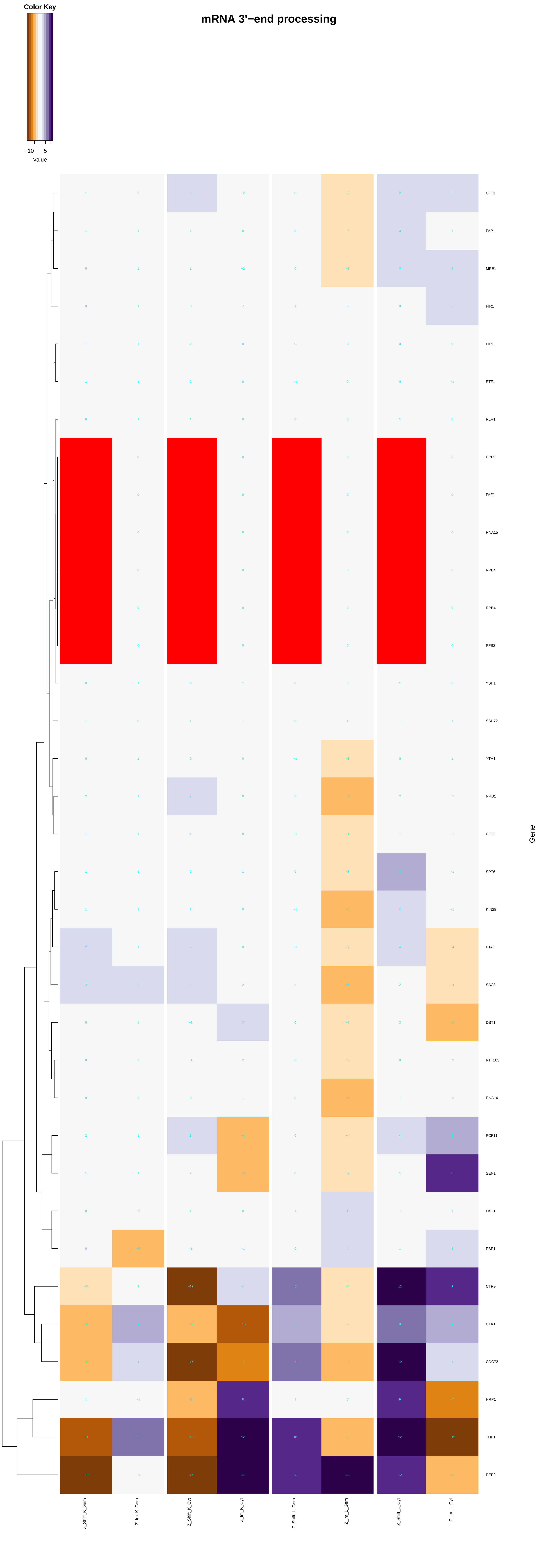
mRNA 3'−end processing
Color Key
−10
5
Value
CFT1
PAP1
MPE1
FIR1
FIP1
RTF1
RLR1
HPR1
PAF1
RNA15
RPB4
RPB4
PFS2
YSH1
SSU72
YTH1
NRD1
CFT2
SPT6
KIN28
PTA1
SAC3
DST1
RTT103
RNA14
PCF11
SEN1
FKH1
PBP1
CTR9
CTK1
CDC73
HRP1
THP1
REF2
Gene
Z_Shift_K_Gem
Z_Im_K_Gem
Z_Shift_K_Cyt
Z_Im_K_Cyt
Z_Shift_L_Gem
Z_Im_L_Gem
Z_Shift_L_Cyt
Z_Im_L_Cyt

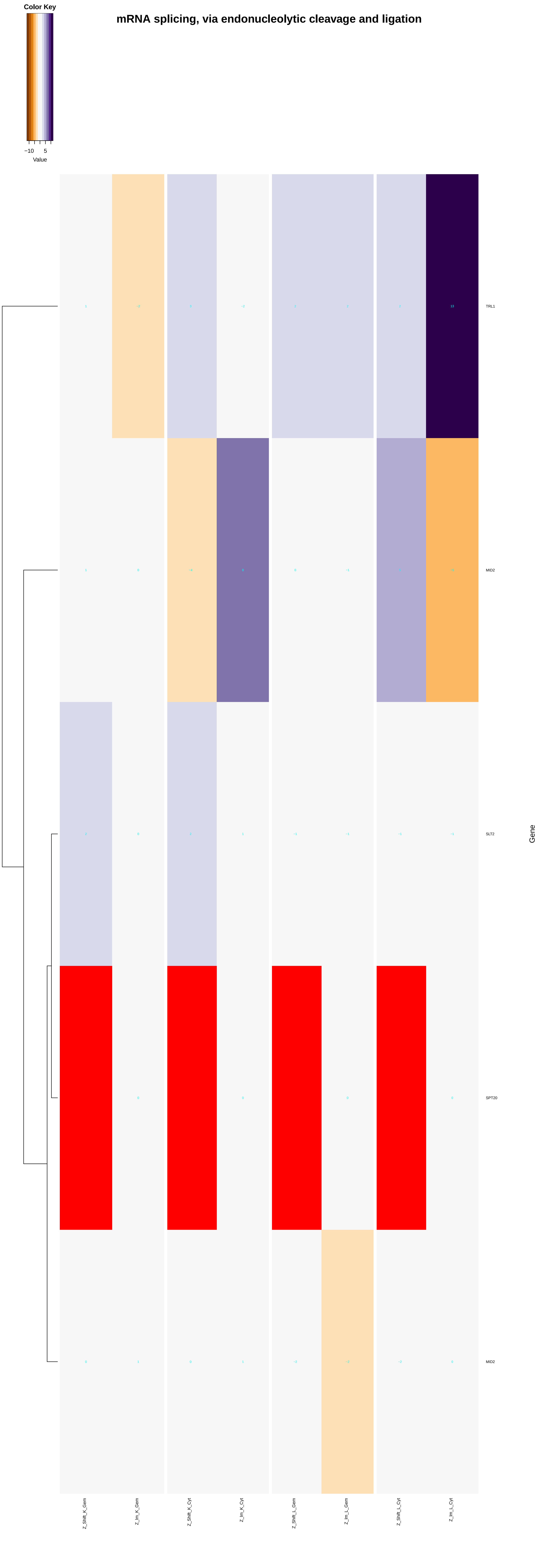
mRNA splicing, via endonucleolytic cleavage and ligation
Color Key
−10
5
Value
TRL1
MID2
SLT2
SPT20
MID2
Gene
Z_Shift_K_Gem
Z_Im_K_Gem
Z_Shift_K_Cyt
Z_Im_K_Cyt
Z_Shift_L_Gem
Z_Im_L_Gem
Z_Shift_L_Cyt
Z_Im_L_Cyt

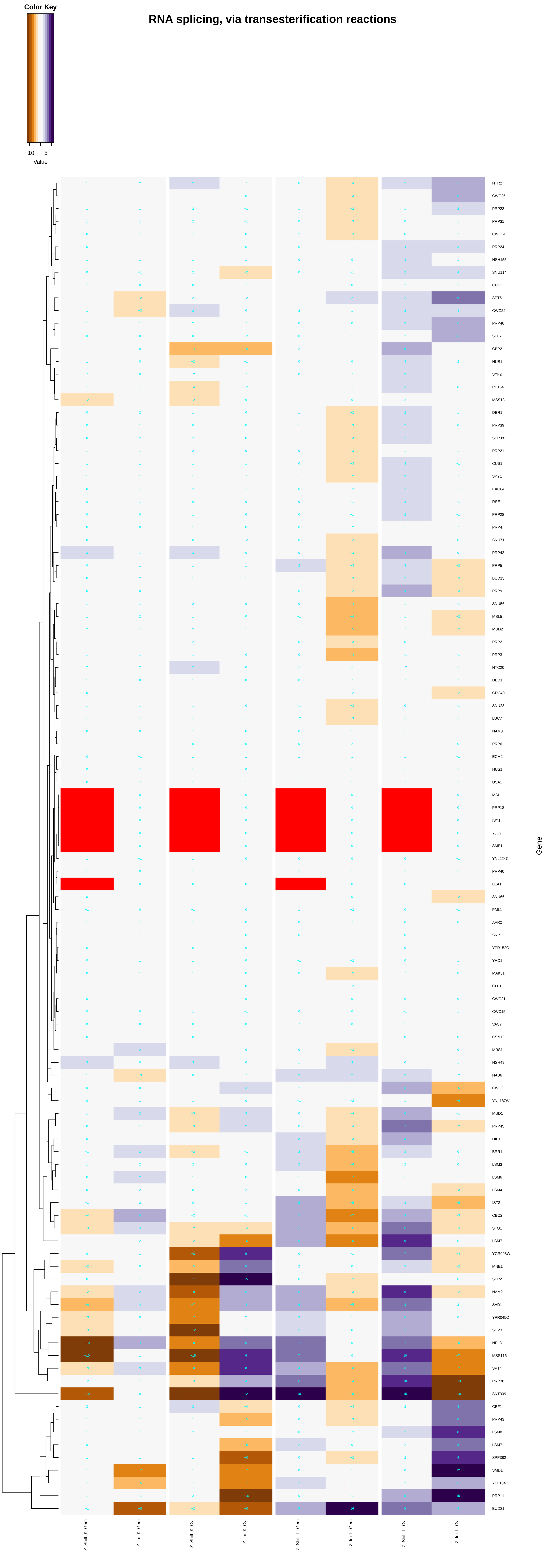
RNA splicing, via transesterification reactions
Color Key
−10
5
Value
NTR2
CWC25
PRP22
PRP31
CWC24
PRP24
HSH155
SNU114
CUS2
SPT5
CWC22
PRP46
SLU7
CBP2
HUB1
SYF2
PET54
MSS18
DBR1
PRP39
SPP381
PRP21
CUS1
SKY1
EXO84
RSE1
PRP28
PRP4
SNU71
PRP42
PRP5
BUD13
PRP9
SNU56
MSL5
MUD2
PRP2
PRP3
NTC20
DED1
CDC40
SNU23
LUC7
NAM8
PRP6
ECM2
HUS1
USA1
MSL1
PRP18
ISY1
YJU2
SME1
YNL224C
PRP40
LEA1
SNU66
PML1
AAR2
SNP1
YPR152C
YHC1
MAK31
CLF1
CWC21
CWC15
VAC7
CSN12
MRS1
HSH49
NAB6
CWC2
YNL187W
MUD1
PRP45
DIB1
BRR1
LSM3
LSM6
LSM4
IST3
CBC2
STO1
LSM7
YGR093W
MNE1
SPP2
NAM2
SAD1
YPR045C
SUV3
NPL3
MSS116
SPT4
PRP38
SNT309
CEF1
PRP43
LSM8
LSM7
SPP382
SMD1
YPL184C
PRP11
BUD31
Gene
Z_Shift_K_Gem
Z_Im_K_Gem
Z_Shift_K_Cyt
Z_Im_K_Cyt
Z_Shift_L_Gem
Z_Im_L_Gem
Z_Shift_L_Cyt
Z_Im_L_Cyt

RNA splicing, via endonucleolytic cleavage and ligation

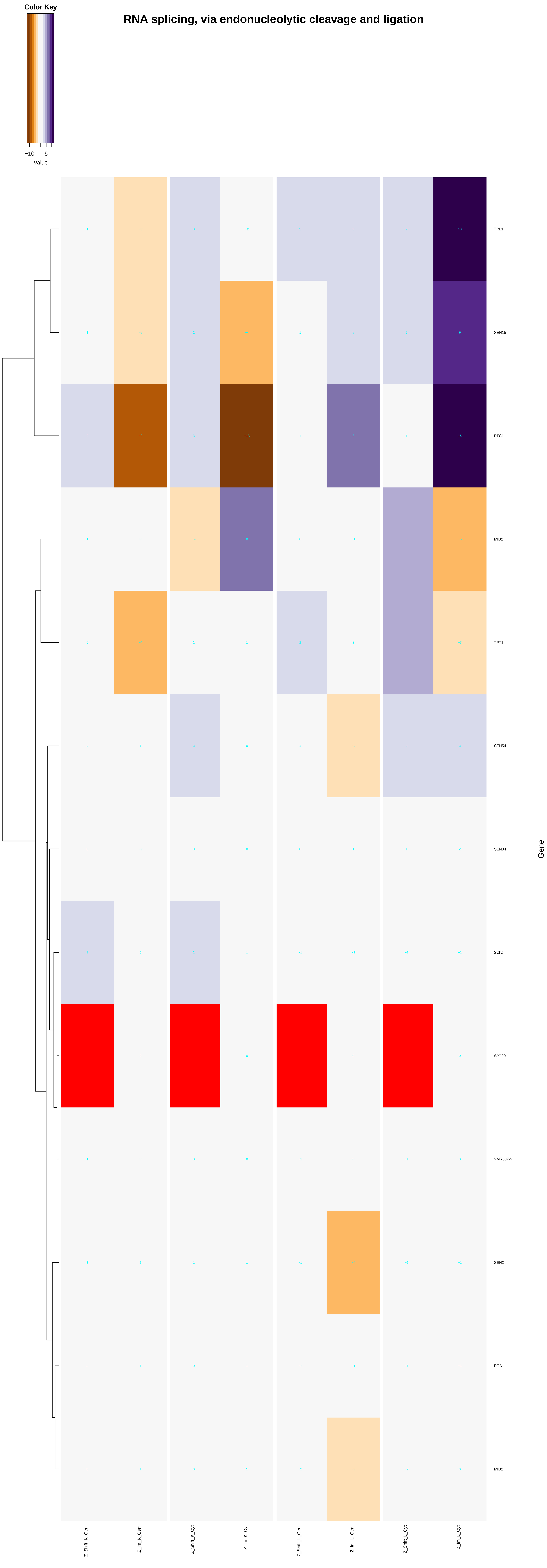

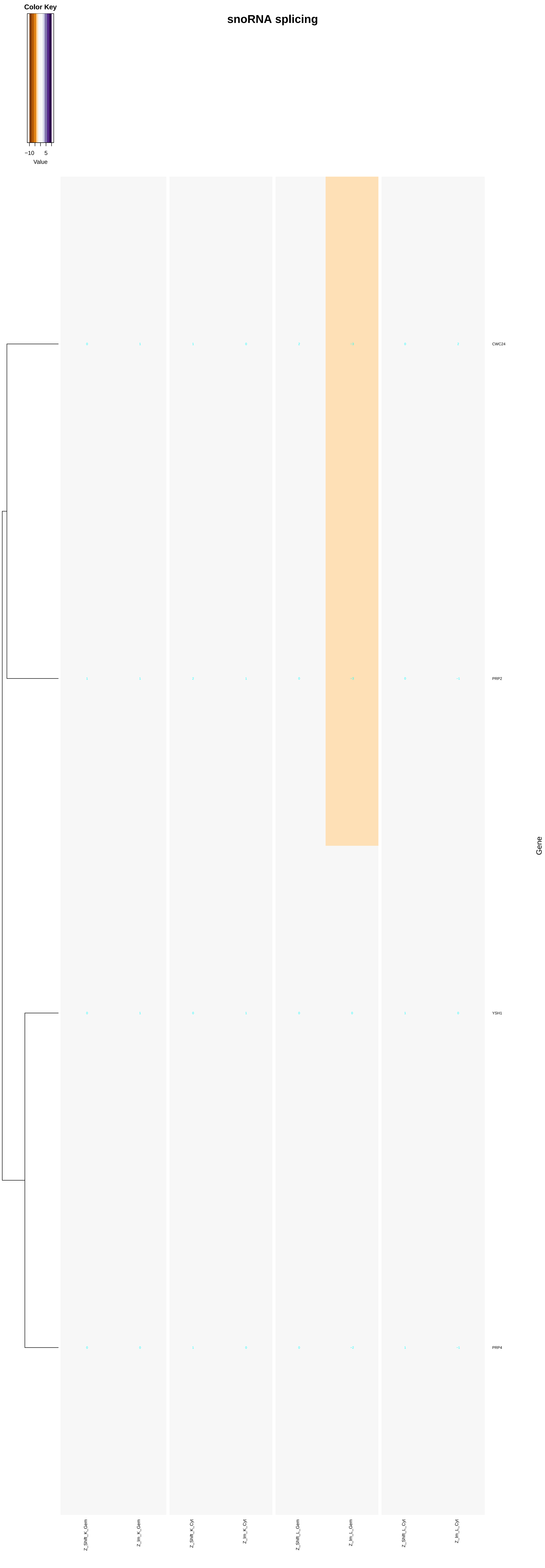
snoRNA splicing
Color Key
−10
5
Value
CWC24
PRP2
YSH1
PRP4
Gene
Z_Shift_K_Gem
Z_Im_K_Gem
Z_Shift_K_Cyt
Z_Im_K_Cyt
Z_Shift_L_Gem
Z_Im_L_Gem
Z_Shift_L_Cyt
Z_Im_L_Cyt

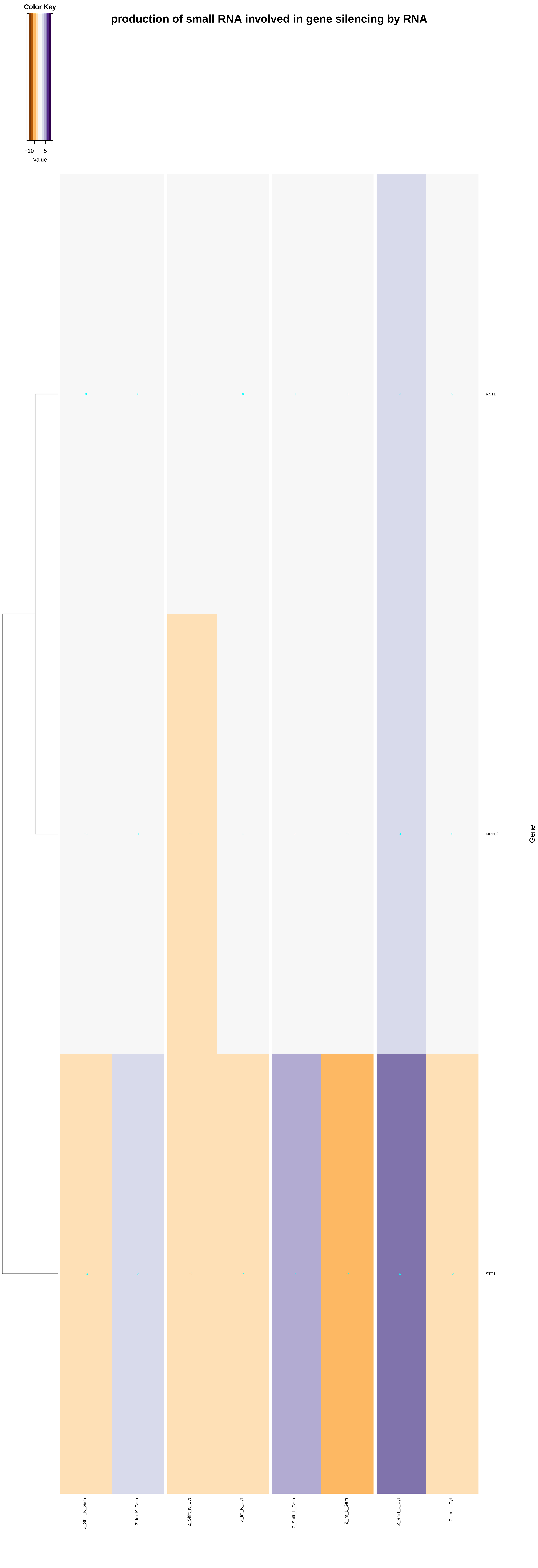

production of small RNA involved in gene silencing by RNA
Color Key
−10
5
Value
RNT1
MRPL3
STO1
Gene
Z_Shift_K_Gem
Z_Im_K_Gem
Z_Shift_K_Cyt
Z_Im_K_Cyt
Z_Shift_L_Gem
Z_Im_L_Gem
Z_Shift_L_Cyt
Z_Im_L_Cyt

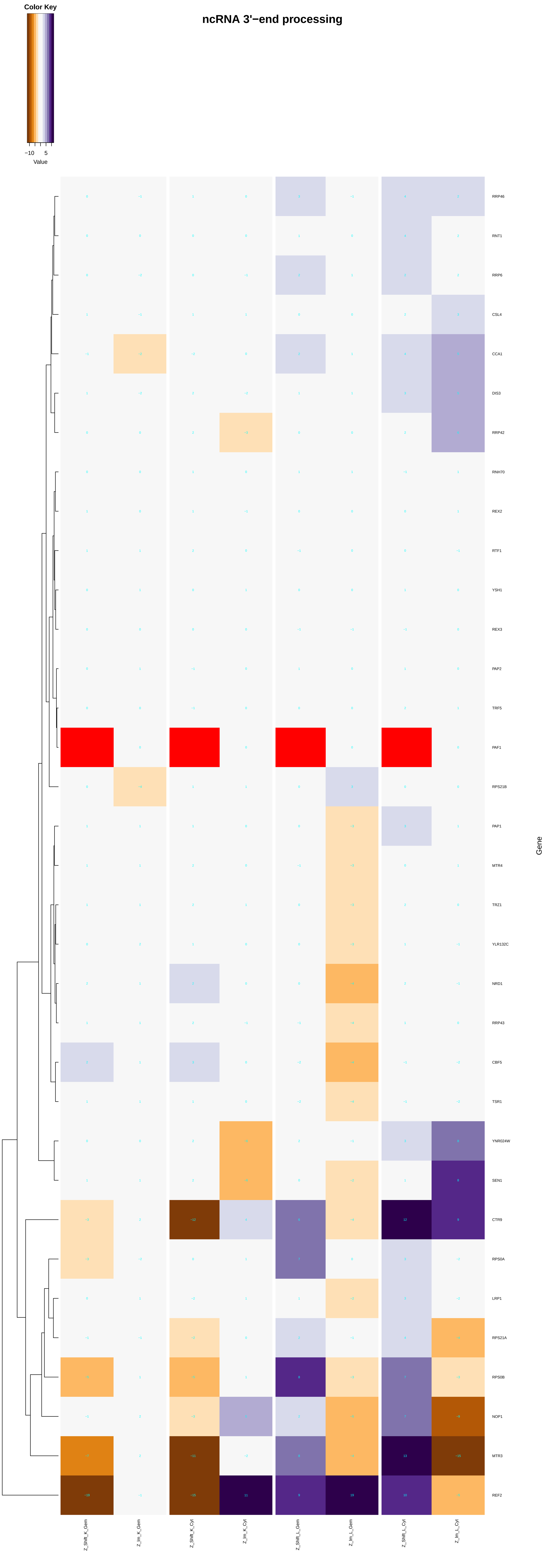

ncRNA 3'−end processing
Color Key
−10
5
Value
RRP46
RNT1
RRP6
CSL4
CCA1
DIS3
RRP42
RNH70
REX2
RTF1
YSH1
REX3
PAP2
TRF5
PAF1
RPS21B
PAP1
MTR4
TRZ1
YLR132C
NRD1
RRP43
CBF5
TSR1
YNR024W
SEN1
CTR9
RPS0A
LRP1
RPS21A
RPS0B
NOP1
MTR3
REF2
Gene
Z_Shift_K_Gem
Z_Im_K_Gem
Z_Shift_K_Cyt
Z_Im_K_Cyt
Z_Shift_L_Gem
Z_Im_L_Gem
Z_Shift_L_Cyt
Z_Im_L_Cyt

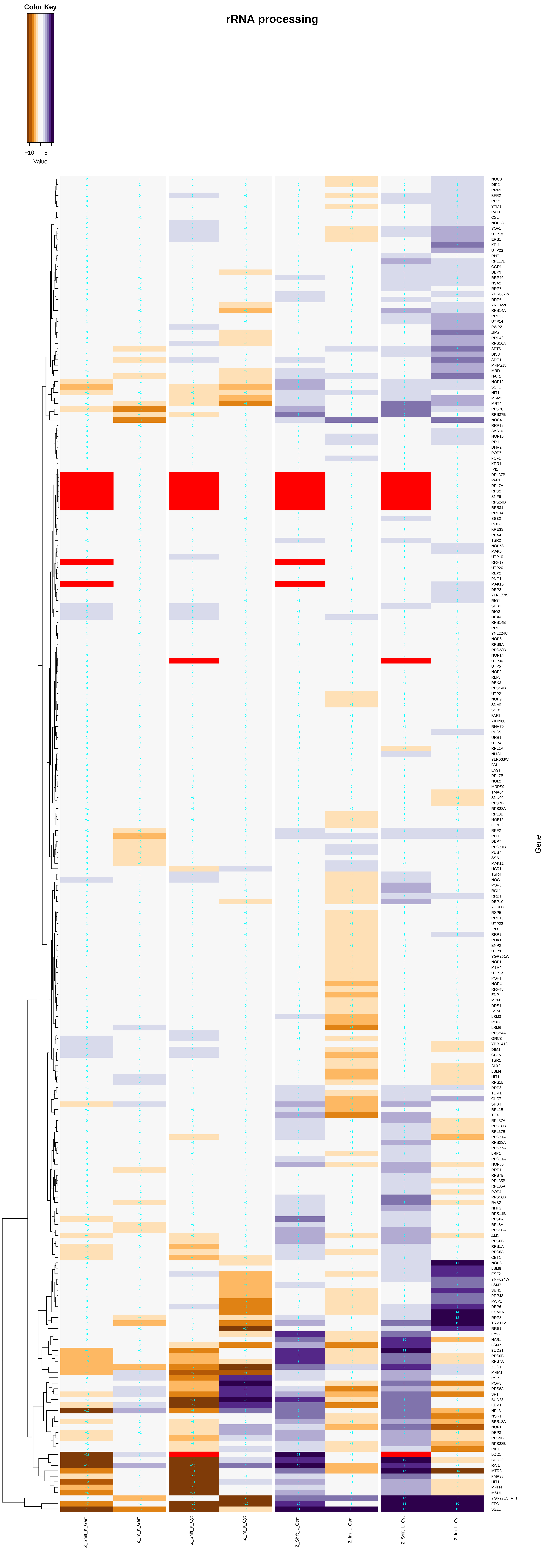
rRNA processing
Color Key
−10
5
Value
Gene
NOC3
DIP2
RMP1
BFR2
RPP1
YTM1
RAT1
CSL4
NOP58
SOF1
UTP15
ERB1
KRI1
UTP23
RNT1
RPL17B
CGR1
DBP9
RRP46
NSA2
RRP7
YHR087W
RRP6
YNL022C
RPS14A
RRP36
UTP14
PWP2
JIP5
RRP42
RPS16A
SPT5
DIS3
SDO1
MRPS18
MRD1
NAF1
NOP12
SSF1
HIT1
MRM2
MRT4
RPS20
RPS27B
NOC4
RRP12
SAS10
NOP16
RIX1
DHR2
POP7
FCF1
KRR1
IPI1
RPL37B
PAF1
RPL7A
RPS2
SNF6
RPS24B
RPS31
RRP14
SSB2
POP8
KRE33
REX4
TSR2
NOP53
MAK5
UTP10
RRP17
UTP20
REX2
PNO1
MAK16
DBP2
YLR177W
RIO1
SPB1
RIO2
HCA4
RPS14B
RRP5
YNL224C
NOP6
RPS9A
RPS23B
NOP14
UTP30
UTP5
NOP2
RLP7
REX3
RPS14B
UTP21
NOP9
SNM1
SSD1
FAF1
YIL096C
RNH70
PUS5
URB1
UTP4
RPL1A
NUG1
YLR063W
FAL1
LAS1
RPL7B
NGL2
MRPS9
TMA64
SNU66
RPS7B
RPS28A
RPL8B
NOP15
FUN12
RPF2
RLI1
DBP7
RPS21B
PUS7
SSB1
MAK11
HCR1
TSR4
NOG1
POP5
RCL1
RRB1
DBP10
YOR006C
RSP5
RRP15
UTP22
IPI3
RRP9
ROK1
ENP2
UTP9
YGR251W
NOB1
MTR4
UTP13
POP1
NOP4
RRP43
ENP1
MDN1
DRS1
IMP4
LSM3
POP6
LSM6
RPS24A
GRC3
YBR141C
DIM1
CBF5
TSR1
SLX9
LSM4
HIT1
RPS1B
RRP8
TOM1
GLC7
SPB4
RPL1B
TIF6
RPL37A
RPS18B
RPL37B
RPS21A
RPS23A
RPS27A
LRP1
RPS11A
NOP56
RRP1
RPS7B
RPL35B
RPL35A
POP4
RPS16B
RVB2
NHP2
RPS11B
RPS0A
RPL8A
RPS16A
JJJ1
RPS6B
RPS1A
RPS6A
CBT1
NOP8
LSM8
ESF2
YNR024W
LSM7
SEN1
PRP43
PWP1
DBP6
ECM16
RRP3
TRM112
RRS1
FYV7
HAS1
LSM7
BUD21
RPS0B
RPS7A
ZUO1
MRM1
PSP1
POP3
RPS8A
SPT4
BUD23
KEM1
NPL3
NSR1
RPS18A
NOP1
DBP3
RPS9B
RPS28B
PIH1
LOC1
BUD22
RAI1
MTR3
FMP38
HIT1
MRH4
MSU1
YGR271C−A_1
EFG1
SSZ1
Z_Shift_K_Gem
Z_Im_K_Gem
Z_Shift_K_Cyt
Z_Im_K_Cyt
Z_Shift_L_Gem
Z_Im_L_Gem
Z_Shift_L_Cyt
Z_Im_L_Cyt

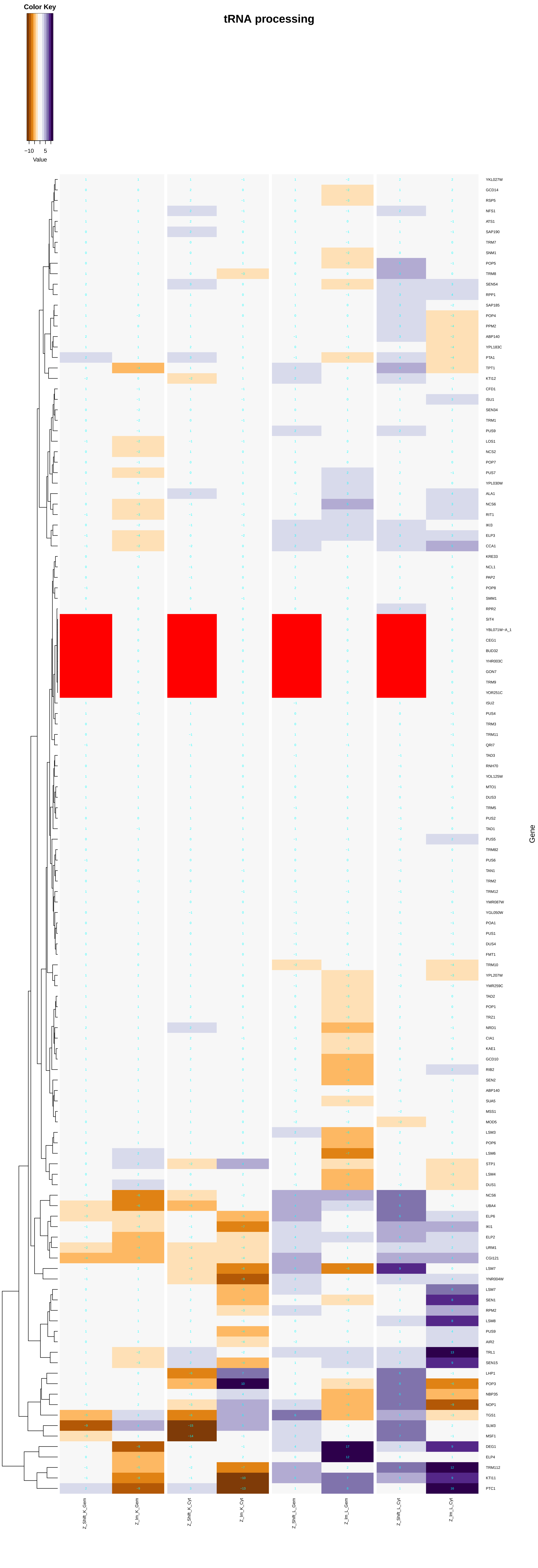
Color Key
−10
5
Value
tRNA processing
YKL027W
GCD14
RSP5
NFS1
ATS1
SAP190
TRM7
SNM1
POP5
TRM8
SEN54
RPP1
SAP185
POP4
PPM2
ABP140
YPL183C
PTA1
TPT1
KTI12
CFD1
ISU1
SEN34
TRM1
PUS9
LOS1
NCS2
POP7
PUS7
YPL030W
ALA1
NCS6
RIT1
IKI3
ELP3
CCA1
KRE33
NCL1
PAP2
POP8
SMM1
RPR2
SIT4
YBL071W−A_1
CEG1
BUD32
YHR003C
GON7
TRM9
YOR251C
ISU2
PUS4
TRM3
TRM11
QRI7
TAD3
RNH70
YOL125W
MTO1
DUS3
TRM5
PUS2
TAD1
PUS5
TRM82
PUS6
TAN1
TRM2
TRM12
YMR087W
YGL050W
POA1
PUS1
DUS4
FMT1
TRM10
YPL207W
YMR259C
TAD2
POP1
TRZ1
NRD1
CIA1
KAE1
GCD10
RIB2
SEN2
ABP140
SUA5
MSS1
MOD5
LSM3
POP6
LSM6
STP1
LSM4
DUS1
NCS6
UBA4
ELP6
IKI1
ELP2
URM1
CGI121
LSM7
YNR004W
LSM7
SEN1
RPM2
LSM8
PUS8
AIR2
TRL1
SEN15
LHP1
POP3
NBP35
NOP1
TGS1
SLM3
MSF1
DEG1
ELP4
TRM112
KTI11
PTC1
Gene
Z_Shift_K_Gem
Z_Im_K_Gem
Z_Shift_K_Cyt
Z_Im_K_Cyt
Z_Shift_L_Gem
Z_Im_L_Gem
Z_Shift_L_Cyt
Z_Im_L_Cyt

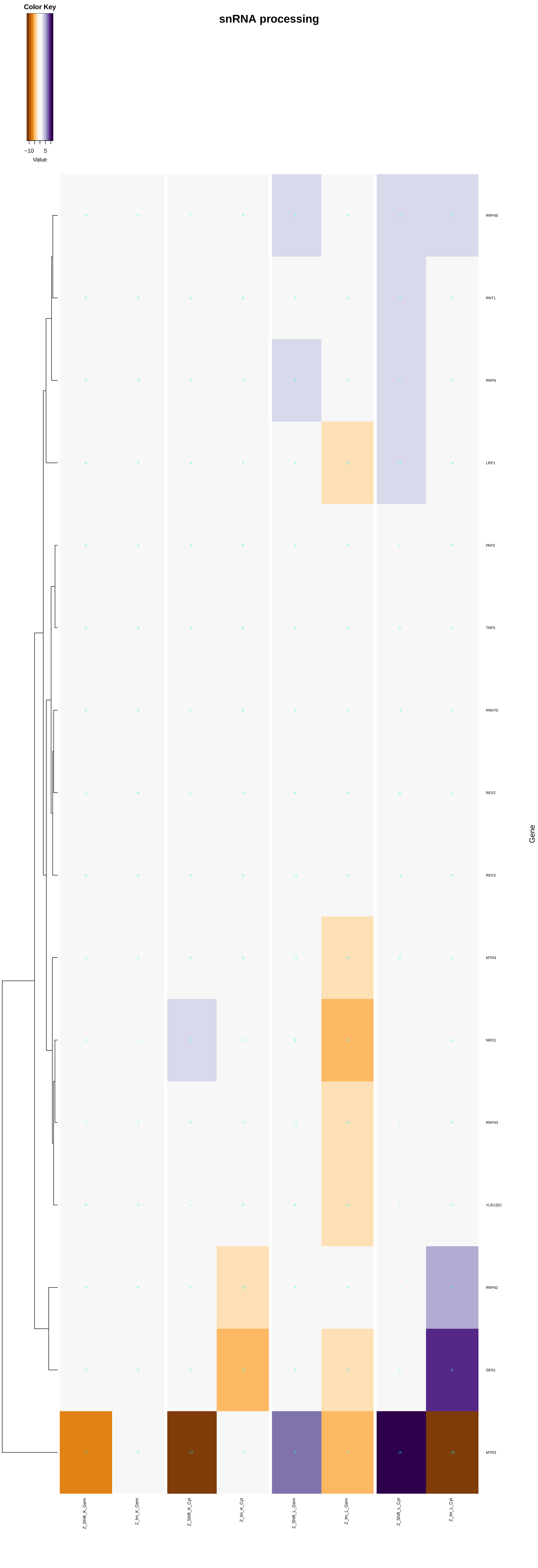
Color Key
−10
5
Value
snRNA processing
RRP46
RNT1
RRP6
LRP1
PAP2
TRF5
RNH70
REX2
REX3
MTR4
NRD1
RRP43
YLR132C
RRP42
SEN1
MTR3
Gene
Z_Shift_K_Gem
Z_Im_K_Gem
Z_Shift_K_Cyt
Z_Im_K_Cyt
Z_Shift_L_Gem
Z_Im_L_Gem
Z_Shift_L_Cyt
Z_Im_L_Cyt

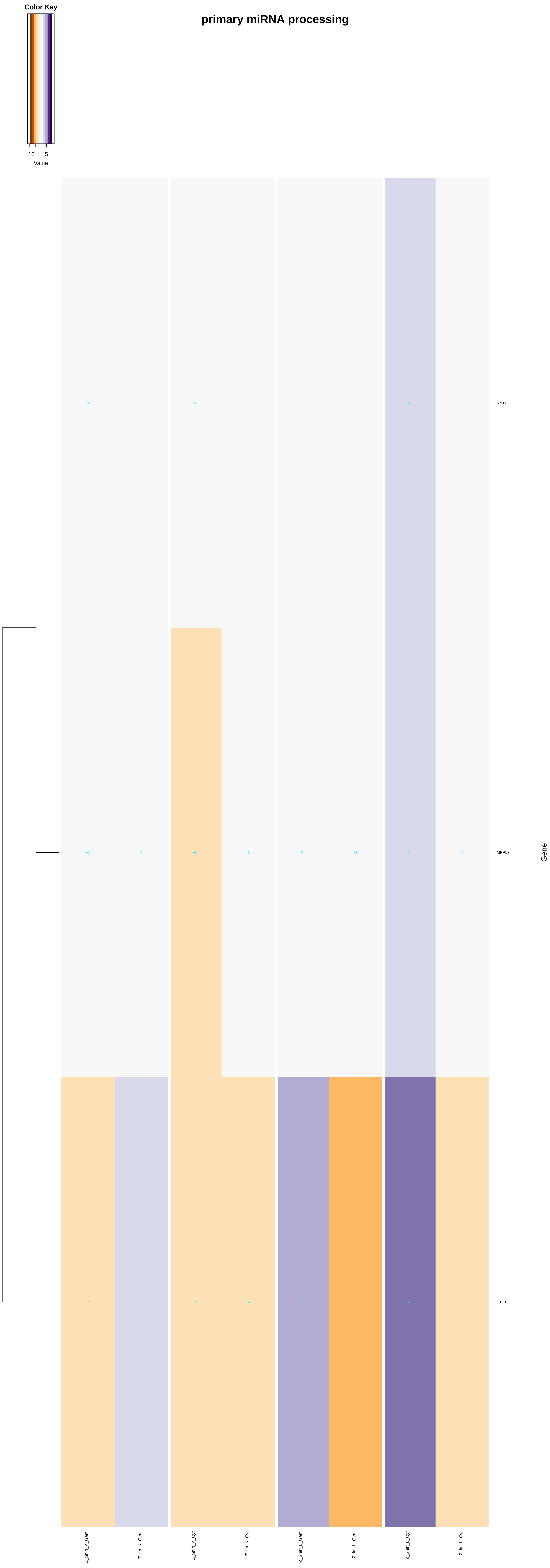
primary miRNA processing
Color Key
−10
5
Value
RNT1
MRPL3
STO1
Gene
Z_Shift_K_Gem
Z_Im_K_Gem
Z_Shift_K_Cyt
Z_Im_K_Cyt
Z_Shift_L_Gem
Z_Im_L_Gem
Z_Shift_L_Cyt
Z_Im_L_Cyt

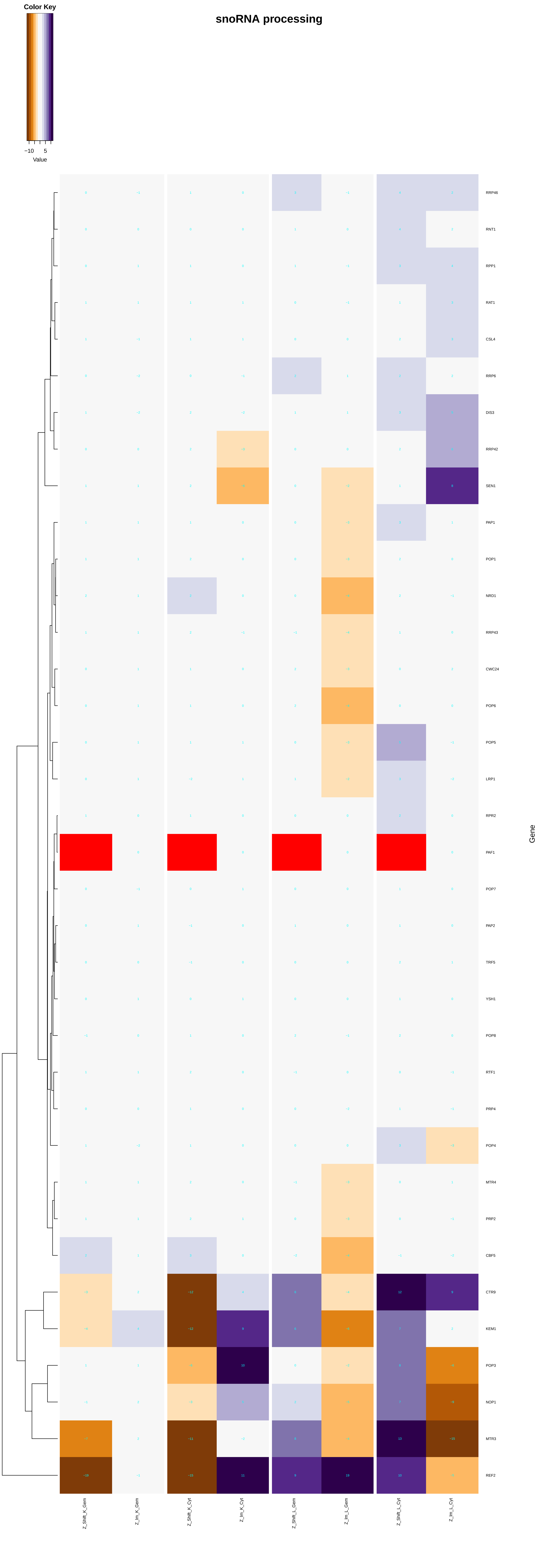
snoRNA processing
Color Key
−10
5
Value
RRP46
RNT1
RPP1
RAT1
CSL4
RRP6
DIS3
RRP42
SEN1
PAP1
POP1
NRD1
RRP43
CWC24
POP6
POP5
LRP1
RPR2
PAF1
POP7
PAP2
TRF5
YSH1
POP8
RTF1
PRP4
POP4
MTR4
PRP2
CBF5
CTR9
KEM1
POP3
NOP1
MTR3
REF2
Gene
Z_Shift_K_Gem
Z_Im_K_Gem
Z_Shift_K_Cyt
Z_Im_K_Cyt
Z_Shift_L_Gem
Z_Im_L_Gem
Z_Shift_L_Cyt
Z_Im_L_Cyt

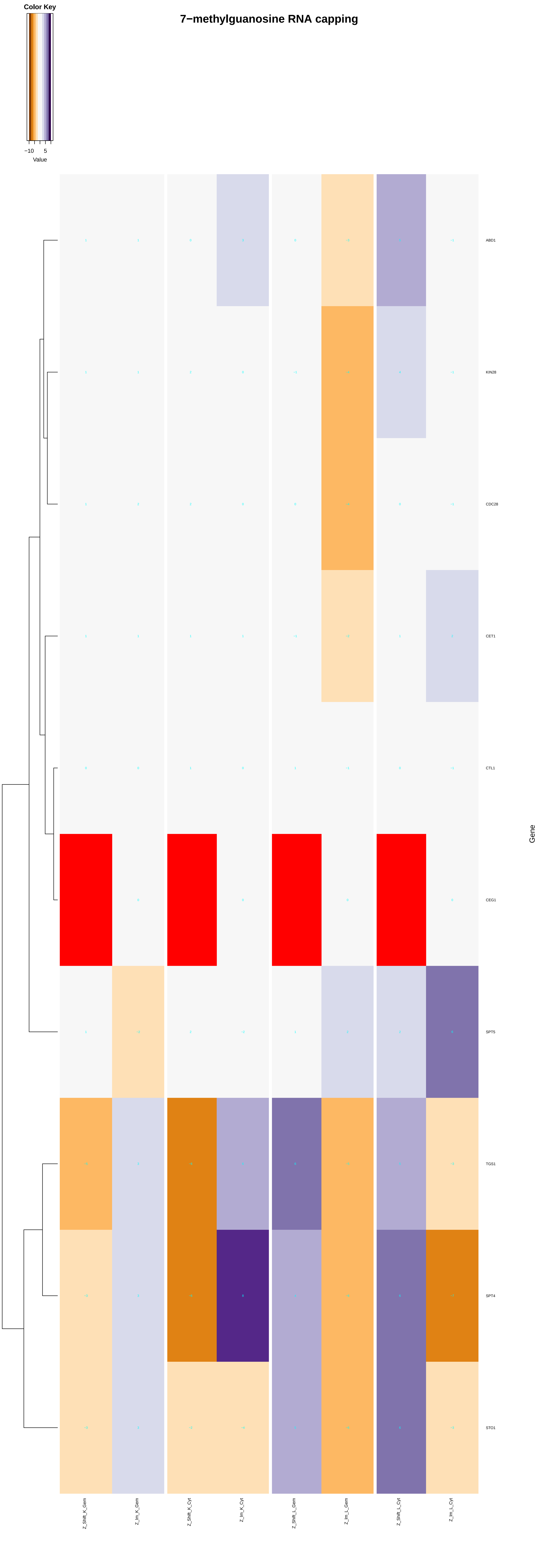

Color Key
−10
5
Value
7−methylguanosine RNA capping
ABD1
KIN28
CDC28
CET1
CTL1
CEG1
SPT5
TGS1
SPT4
STO1
Gene
Z_Shift_K_Gem
Z_lm_K_Gem
Z_Shift_K_Cyt
Z_lm_K_Cyt
Z_Shift_L_Gem
Z_lm_L_Gem
Z_Shift_L_Cyt
Z_lm_L_Cyt

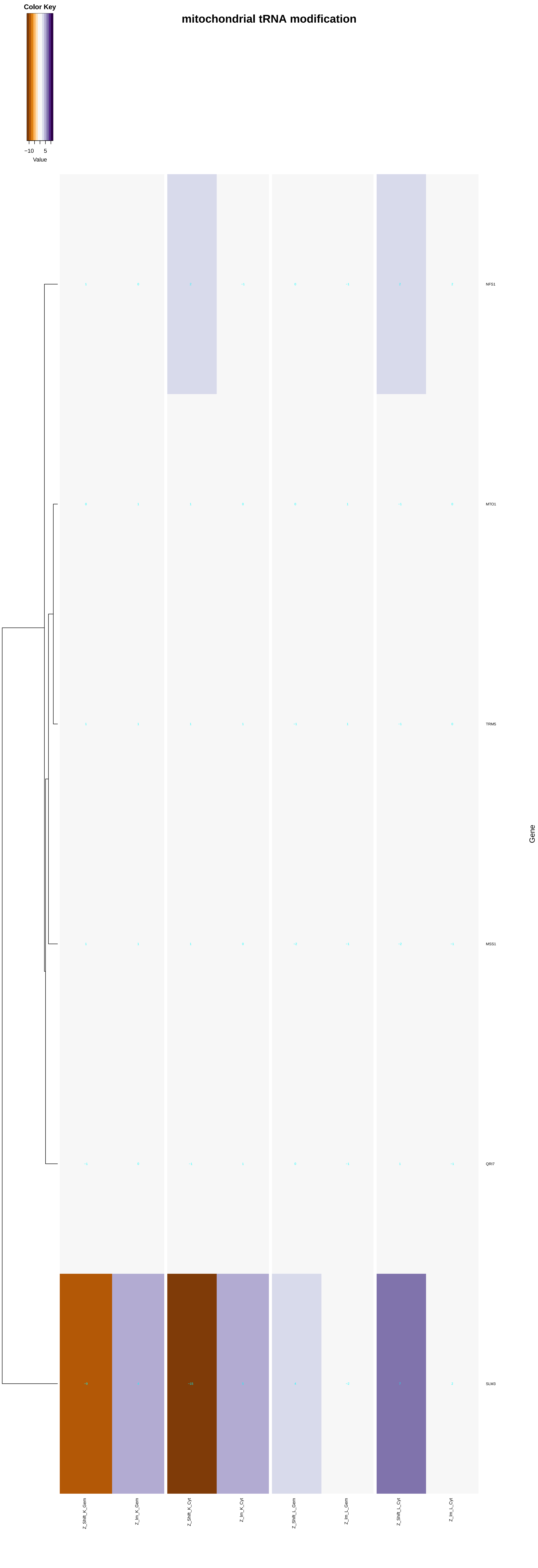

mitochondrial tRNA modification
Color Key
−10
5
Value
NFS1
MTO1
TRM5
MSS1
QRI7
SLM3
Gene
Z_Shift_K_Gem
Z_Im_K_Gem
Z_Shift_K_Cyt
Z_Im_K_Cyt
Z_Shift_L_Gem
Z_Im_L_Gem
Z_Shift_L_Cyt
Z_Im_L_Cyt

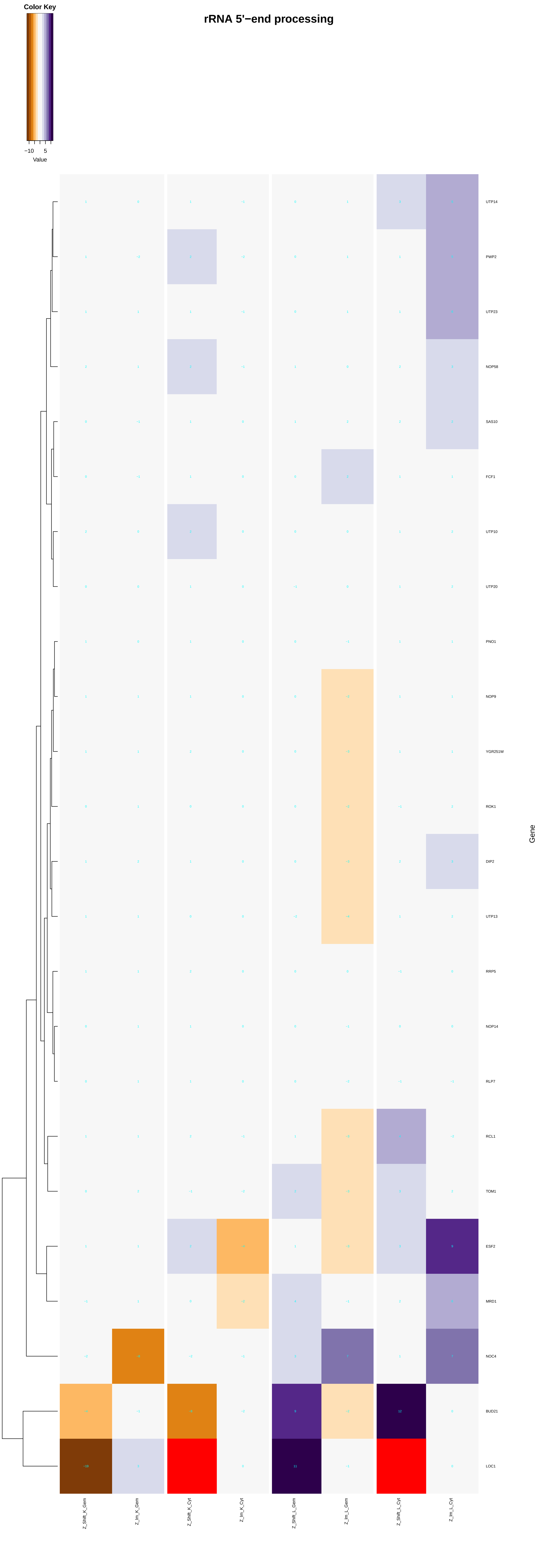

rRNA 5'−end processing
Color Key
−10
5
Value
UTP14
PWP2
UTP23
NOP58
SAS10
FCF1
UTP10
UTP20
PNO1
NOP9
YGR251W
ROK1
DIP2
UTP13
RRP5
NOP14
RLP7
RCL1
TOM1
ESF2
MRD1
NOC4
BUD21
LOC1
Gene
Z_Shift_K_Gem
Z_Im_K_Gem
Z_Shift_K_Cyt
Z_Im_K_Cyt
Z_Shift_L_Gem
Z_Im_L_Gem
Z_Shift_L_Cyt
Z_Im_L_Cyt

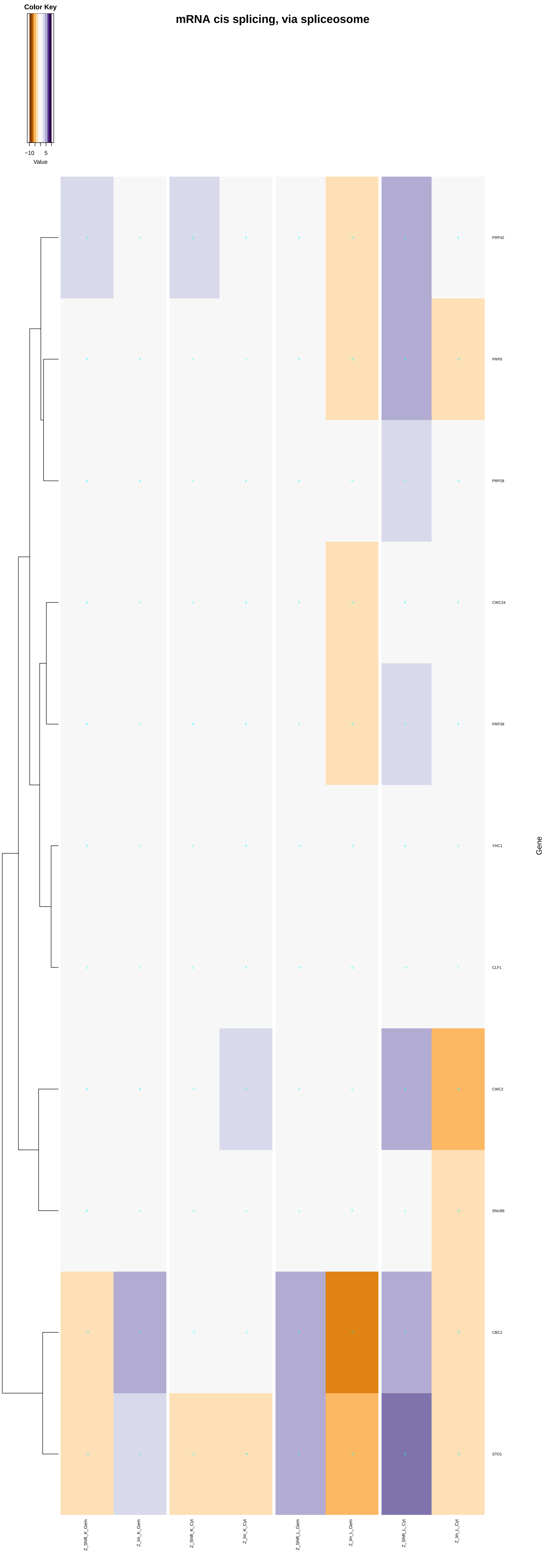
mRNA cis splicing, via spliceosome
Color Key
−10
5
Value
PRP42
PRP9
PRP28
CWC24
PRP39
YHC1
CLF1
CWC2
SNU66
CBC2
STO1
Gene
Z_Shift_K_Gem
Z_Im_K_Gem
Z_Shift_K_Cyt
Z_Im_K_Cyt
Z_Shift_L_Gem
Z_Im_L_Gem
Z_Shift_L_Cyt
Z_Im_L_Cyt

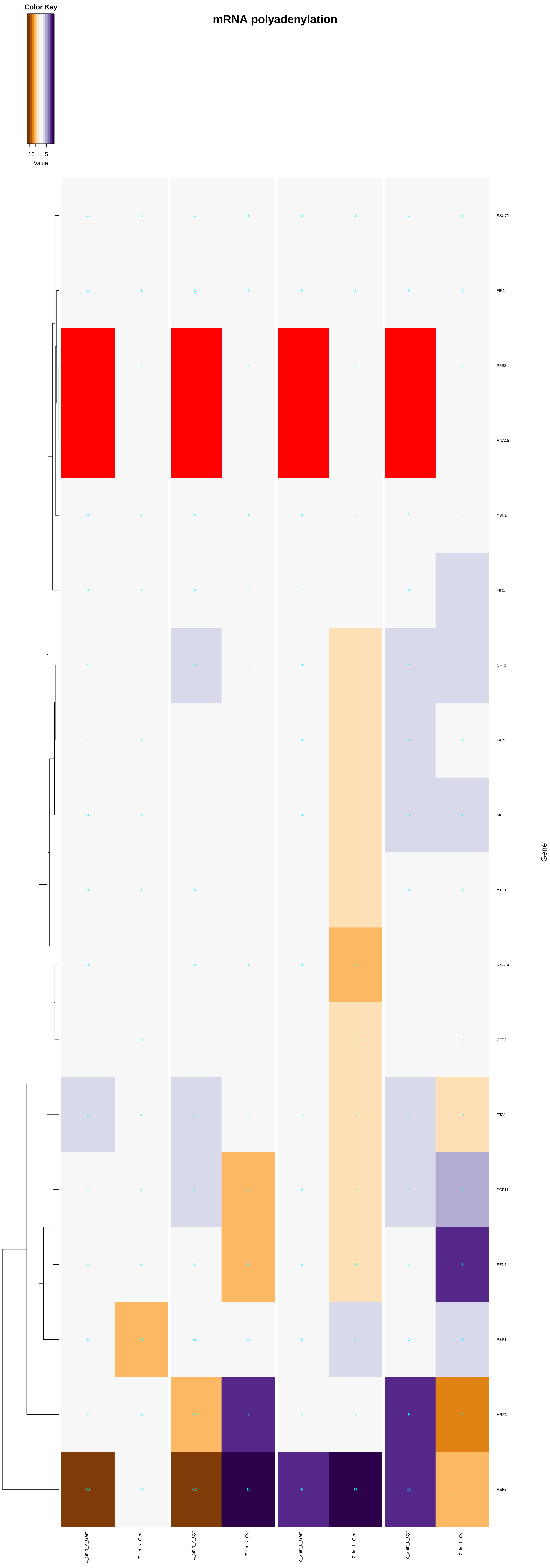
mRNA polyadenylation
Color Key
−10
5
Value
SSU72
FIP1
PFS2
RNA15
YSH1
FIR1
CFT1
PAP1
MPE1
YTH1
RNA14
CFT2
PTA1
PCF11
SEN1
PBP1
HRP1
REF2
Gene
Z_Shift_K_Gem
Z_Im_K_Gem
Z_Shift_K_Cyt
Z_Im_K_Cyt
Z_Shift_L_Gem
Z_Im_L_Gem
Z_Shift_L_Cyt
Z_Im_L_Cyt

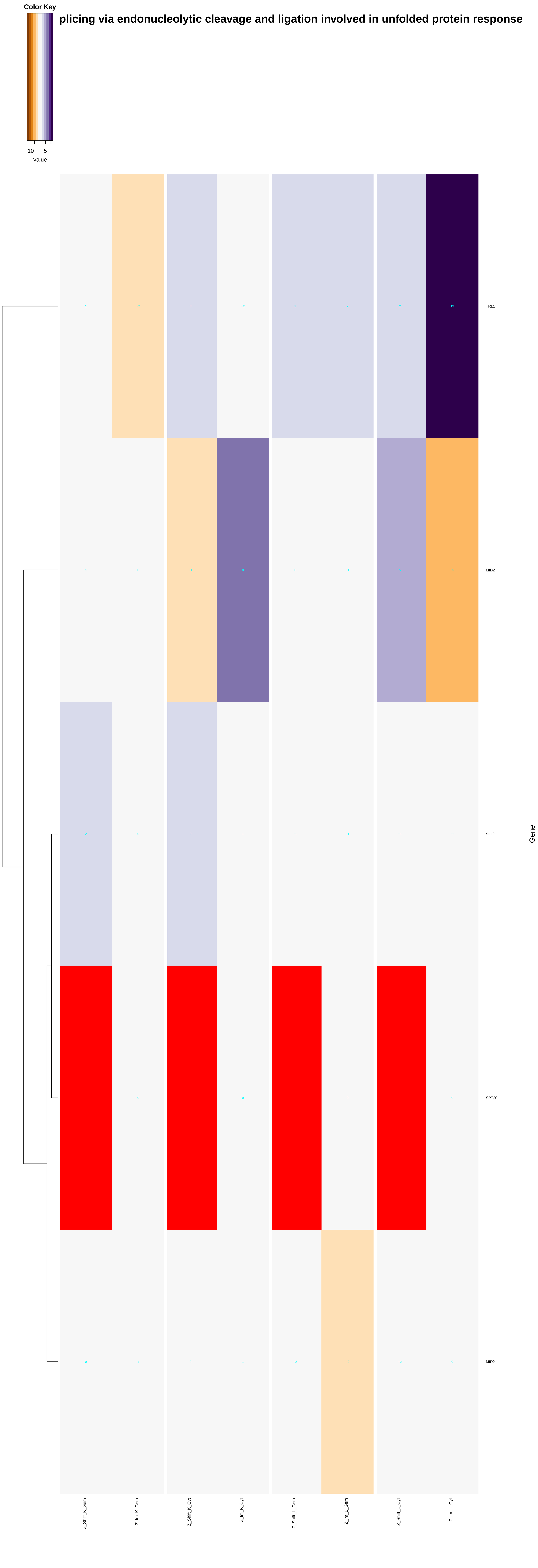

Color Key
−10
5
Value
plicing via endonucleolytic cleavage and ligation involved in unfolded protein response
TRL1
MID2
SLT2
SPT20
MID2
Gene
Z_Shift_K_Gem
Z_Im_K_Gem
Z_Shift_K_Cyt
Z_Im_K_Cyt
Z_Shift_L_Gem
Z_Im_L_Gem
Z_Shift_L_Cyt
Z_Im_L_Cyt

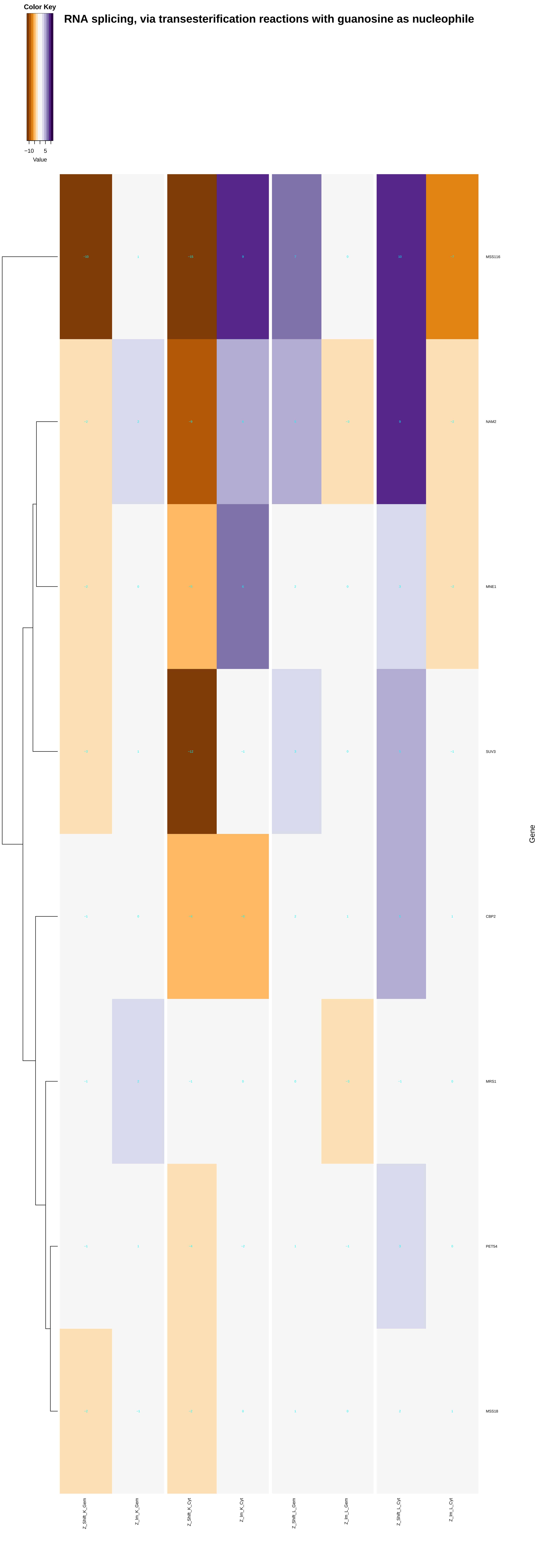

Color Key
−10
5
Value
RNA splicing, via transesterification reactions with guanosine as nucleophile
MSS116
NAM2
MNE1
SUV3
CBP2
MRS1
PET54
MSS18
Gene
Z_Shift_K_Gem
Z_Im_K_Gem
Z_Shift_K_Cyt
Z_Im_K_Cyt
Z_Shift_L_Gem
Z_Im_L_Gem
Z_Shift_L_Cyt
Z_Im_L_Cyt

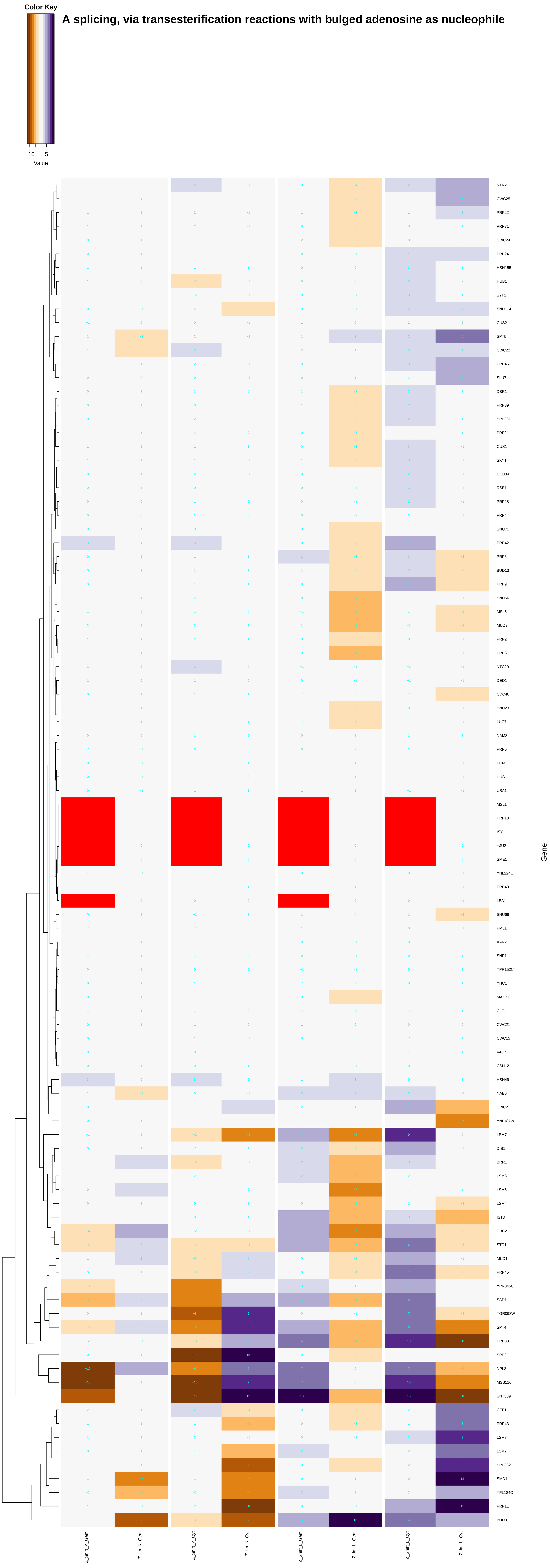
A splicing, via transesterification reactions with bulged adenosine as nucleophile
Color Key
−10
5
Value
NTR2
CWC25
PRP22
PRP31
CWC24
PRP24
HSH155
HUB1
SYF2
SNU114
CUS2
SPT5
CWC22
PRP46
SLU7
DBR1
PRP39
SPP381
PRP21
CUS1
SKY1
EXO84
RSE1
PRP28
PRP4
SNU71
PRP42
PRP5
BUD13
PRP9
SNU56
MSL5
MUD2
PRP2
PRP3
NTC20
DED1
CDC40
SNU23
LUC7
NAM8
PRP6
ECM2
HUS1
USA1
MSL1
PRP18
ISY1
YJU2
SME1
YNL224C
PRP40
LEA1
SNU66
PML1
AAR2
SNP1
YPR152C
YHC1
MAK31
CLF1
CWC21
CWC15
VAC7
CSN12
HSH49
NAB6
CWC2
YNL187W
LSM7
DIB1
BRR1
LSM3
LSM6
LSM4
IST3
CBC2
STO1
MUD1
PRP45
YPR045C
SAD1
YGR093W
SPT4
PRP38
SPP2
NPL3
MSS116
SNT309
CEF1
PRP43
LSM8
LSM7
SPP382
SMD1
YPL184C
PRP11
BUD31
Gene
Z_Shift_K_Gem
Z_Im_K_Gem
Z_Shift_K_Cyt
Z_Im_K_Cyt
Z_Shift_L_Gem
Z_Im_L_Gem
Z_Shift_L_Cyt
Z_Im_L_Cyt

tRNA splicing, via endonucleolytic cleavage and ligation

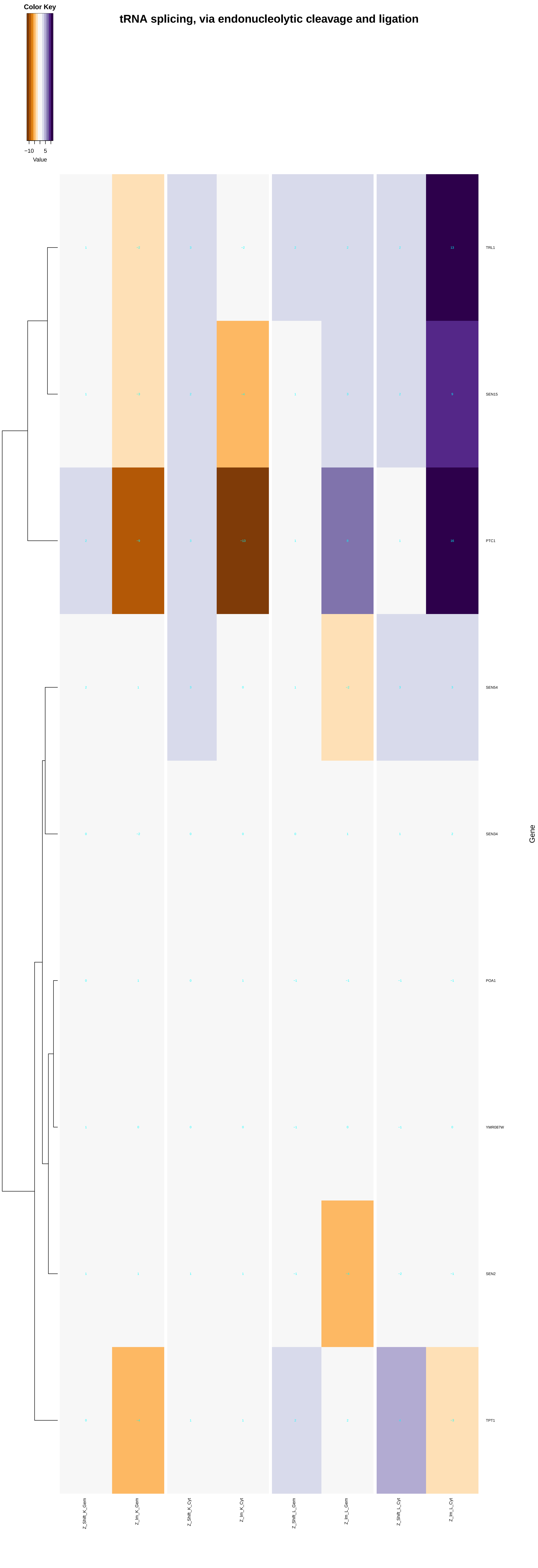

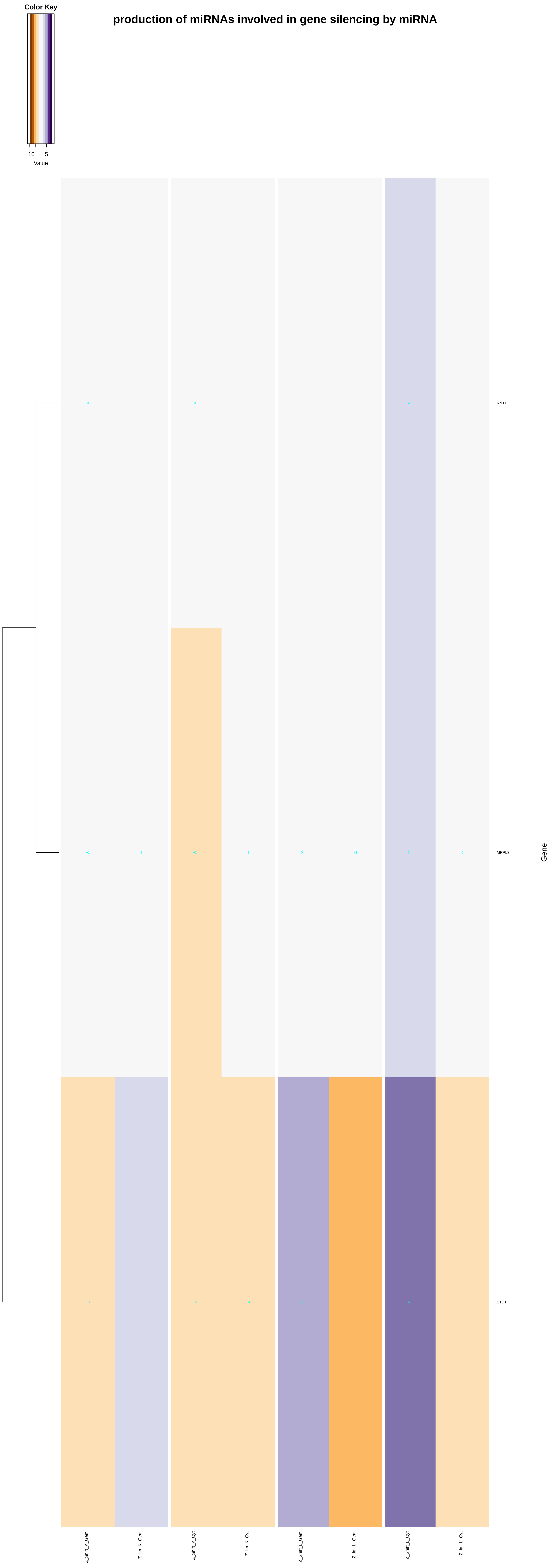
production of miRNAs involved in gene silencing by miRNA
Color Key
−10
5
Value
RNT1
MRPL3
STO1
Gene
Z_Shift_K_Gem
Z_Im_K_Gem
Z_Shift_K_Cyt
Z_Im_K_Cyt
Z_Shift_L_Gem
Z_Im_L_Gem
Z_Shift_L_Cyt
Z_Im_L_Cyt

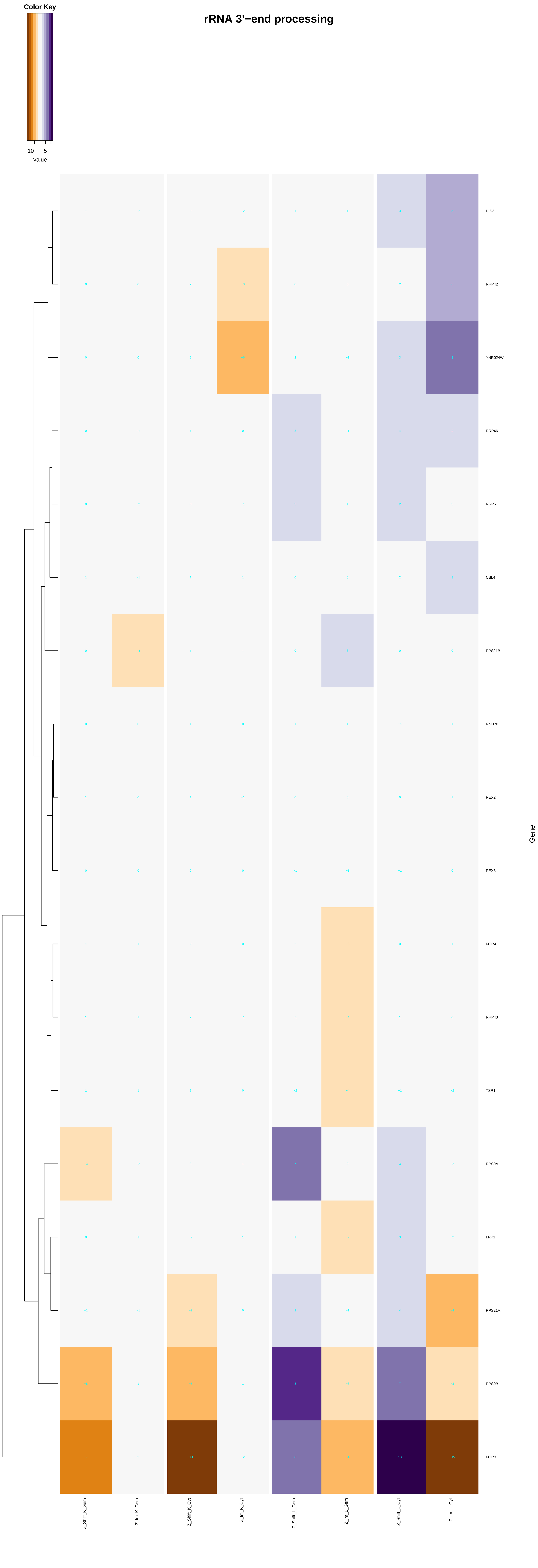

Color Key
−10
5
Value
rRNA 3'−end processing
DIS3
RRP42
YNR024W
RRP46
RRP6
CSL4
RPS21B
RNH70
REX2
REX3
MTR4
RRP43
TSR1
RPS0A
LRP1
RPS21A
RPS0B
MTR3
Gene
Z_Shift_K_Gem
Z_Im_K_Gem
Z_Shift_K_Cyt
Z_Im_K_Cyt
Z_Shift_L_Gem
Z_Im_L_Gem
Z_Shift_L_Cyt
Z_Im_L_Cyt

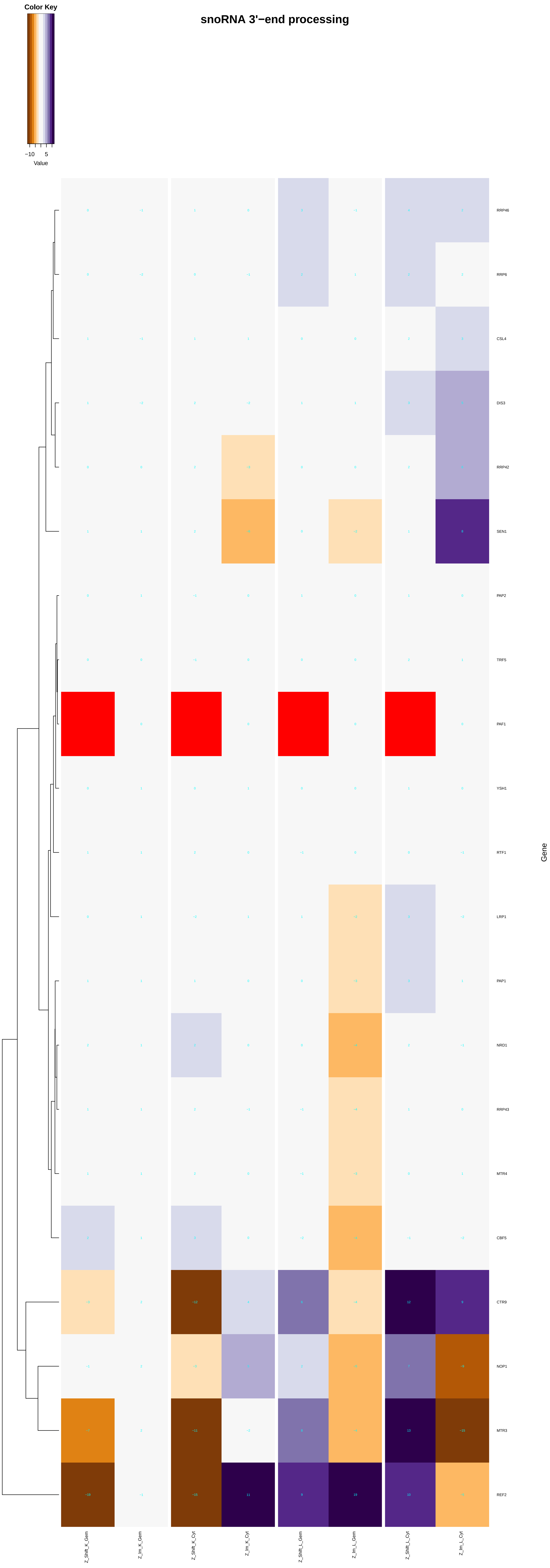

snoRNA 3'−end processing
Color Key
−10
5
Value
Gene
RRP46
RRP6
CSL4
DIS3
RRP42
SEN1
PAP2
TRF5
PAF1
YSH1
RTF1
LRP1
PAP1
NRD1
RRP43
MTR4
CBF5
CTR9
NOP1
MTR3
REF2
Z_Shift_K_Gem
Z_Im_K_Gem
Z_Shift_K_Cyt
Z_Im_K_Cyt
Z_Shift_L_Gem
Z_Im_L_Gem
Z_Shift_L_Cyt
Z_Im_L_Cyt

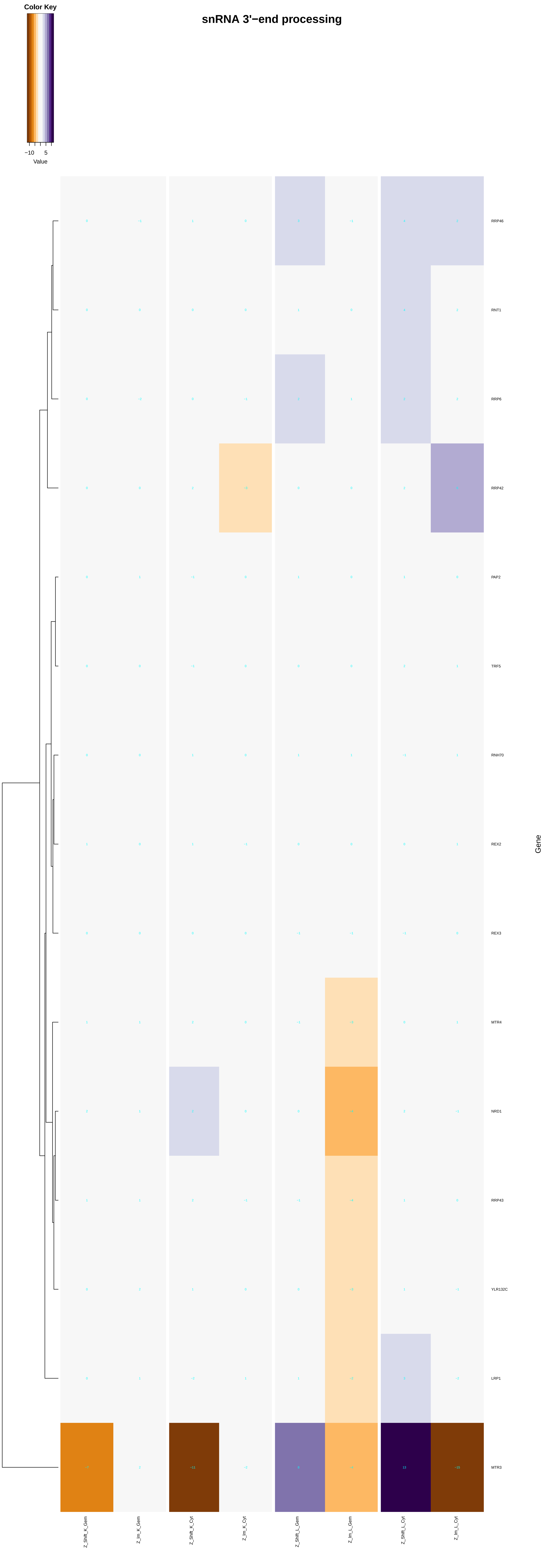
snRNA 3'−end processing
Color Key
−10
5
Value
RRP46
RNT1
RRP6
RRP42
PAP2
TRF5
RNH70
REX2
REX3
MTR4
NRD1
RRP43
YLR132C
LRP1
MTR3
Gene
Z_Shift_K_Gem
Z_lm_K_Gem
Z_Shift_K_Cyt
Z_lm_K_Cyt
Z_Shift_L_Gem
Z_lm_L_Gem
Z_Shift_L_Cyt
Z_lm_L_Cyt

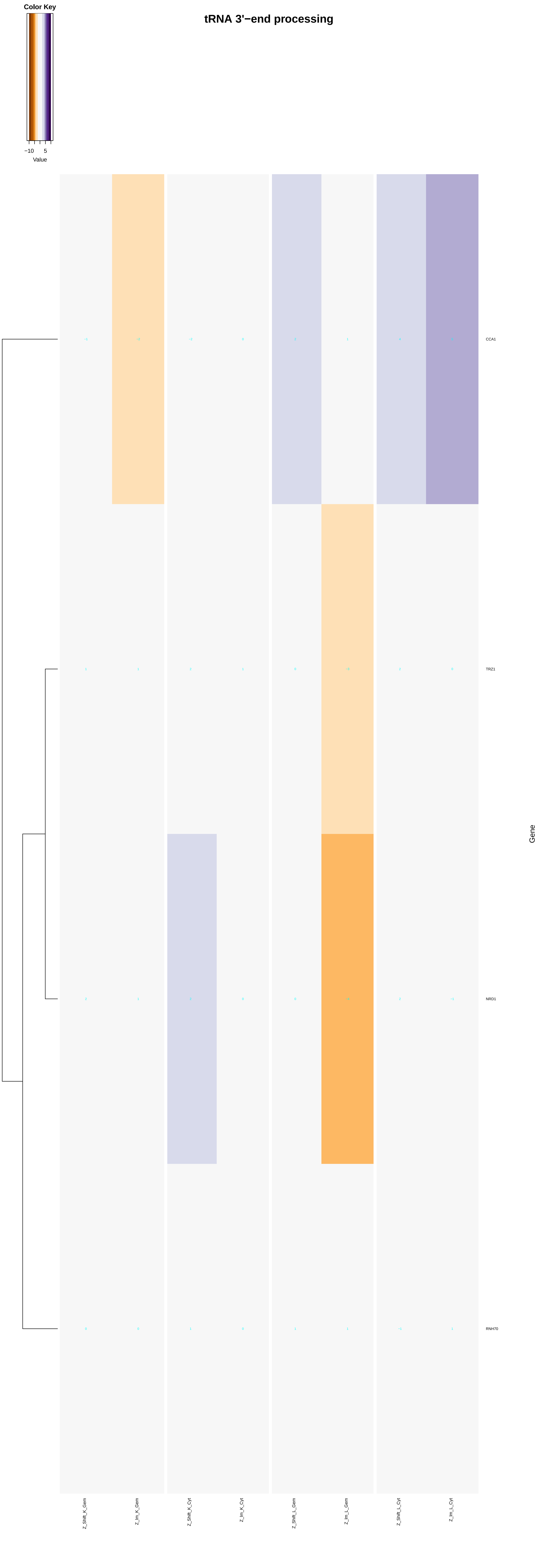
tRNA 3'−end processing
Color Key
−10
5
Value
CCA1
TRZ1
NRD1
RNH70
Gene
Z_Shift_K_Gem
Z_Im_K_Gem
Z_Shift_K_Cyt
Z_Im_K_Cyt
Z_Shift_L_Gem
Z_Im_L_Gem
Z_Shift_L_Cyt
Z_Im_L_Cyt

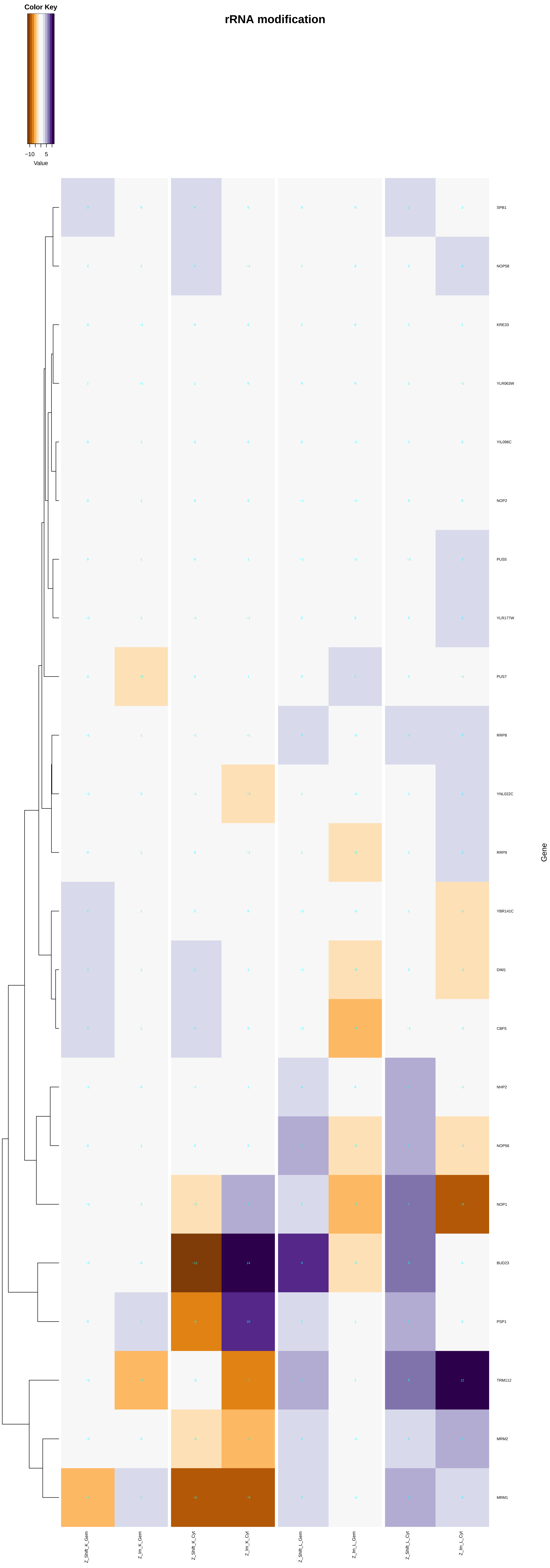

Color Key
−10
5
Value
rRNA modification
SPB1
NOP58
KRE33
YLR063W
YIL096C
NOP2
PUS5
YLR177W
PUS7
RRP8
YNL022C
RRP9
YBR141C
DIM1
CBF5
NHP2
NOP56
NOP1
BUD23
PSP1
TRM112
MRM2
MRM1
Gene
Z_Shift_K_Gem
Z_Im_K_Gem
Z_Shift_K_Cyt
Z_Im_K_Cyt
Z_Shift_L_Gem
Z_Im_L_Gem
Z_Shift_L_Cyt
Z_Im_L_Cyt

maturation of 5.8S rRNA

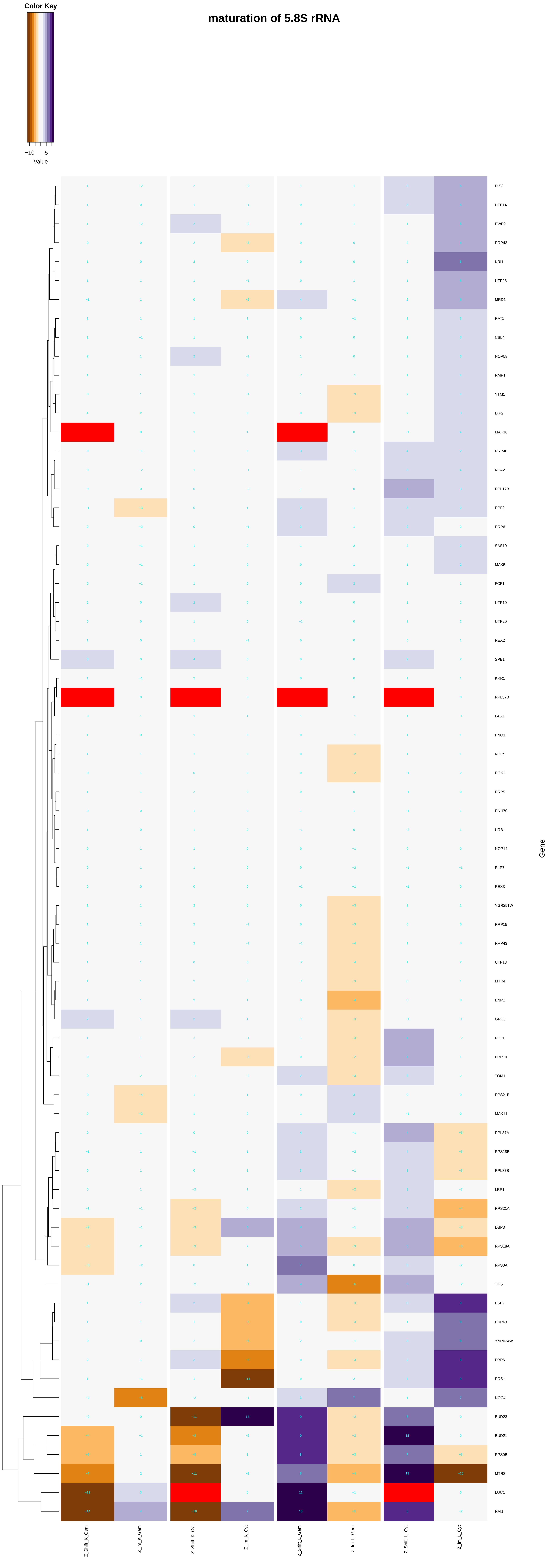

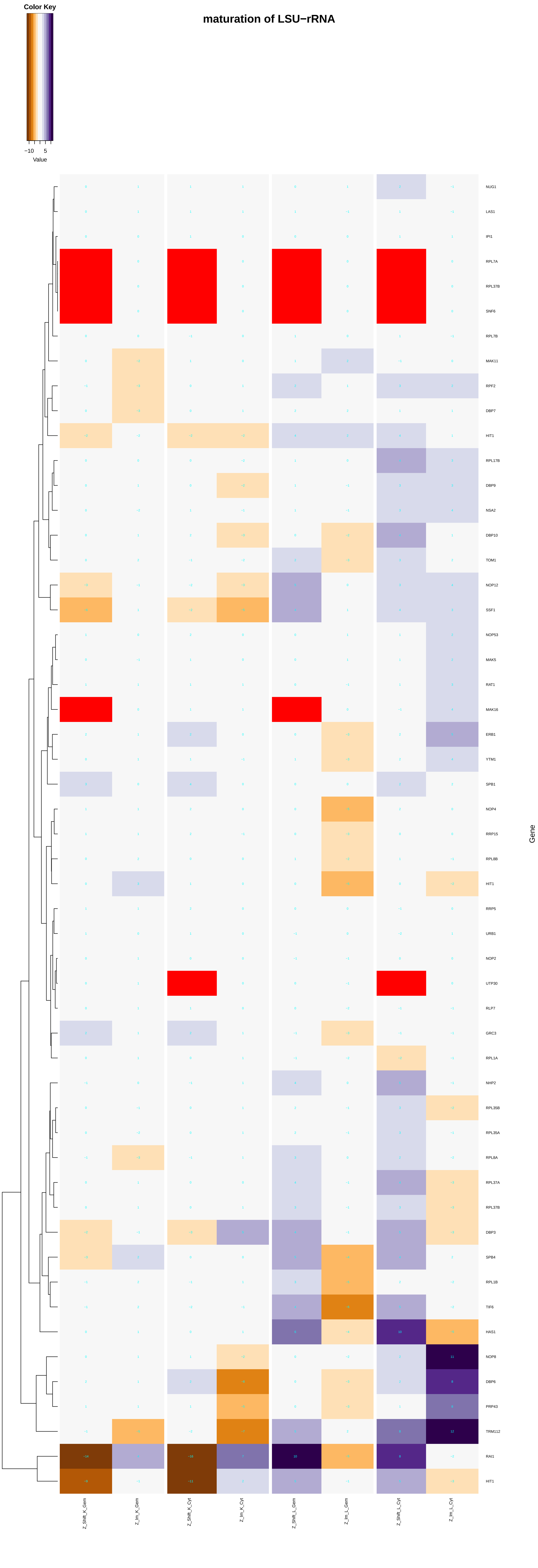

maturation of LSU−rRNA
Color Key
−10
5
Value
NUG1
LAS1
IPI1
RPL7A
RPL37B
SNF6
RPL7B
MAK11
RPF2
DBP7
HIT1
RPL17B
DBP9
NSA2
DBP10
TOM1
NOP12
SSF1
NOP53
MAK5
RAT1
MAK16
ERB1
YTM1
SPB1
NOP4
RRP15
RPL8B
HIT1
RRP5
URB1
NOP2
UTP30
RLP7
GRC3
RPL1A
NHP2
RPL35B
RPL35A
RPL8A
RPL37A
RPL37B
DBP3
SPB4
RPL1B
TIF6
HAS1
NOP8
DBP6
PRP43
TRM112
RAI1
HIT1
Gene
Z_Shift_K_Gem
Z_Im_K_Gem
Z_Shift_K_Cyt
Z_Im_K_Cyt
Z_Shift_L_Gem
Z_Im_L_Gem
Z_Shift_L_Cyt
Z_Im_L_Cyt

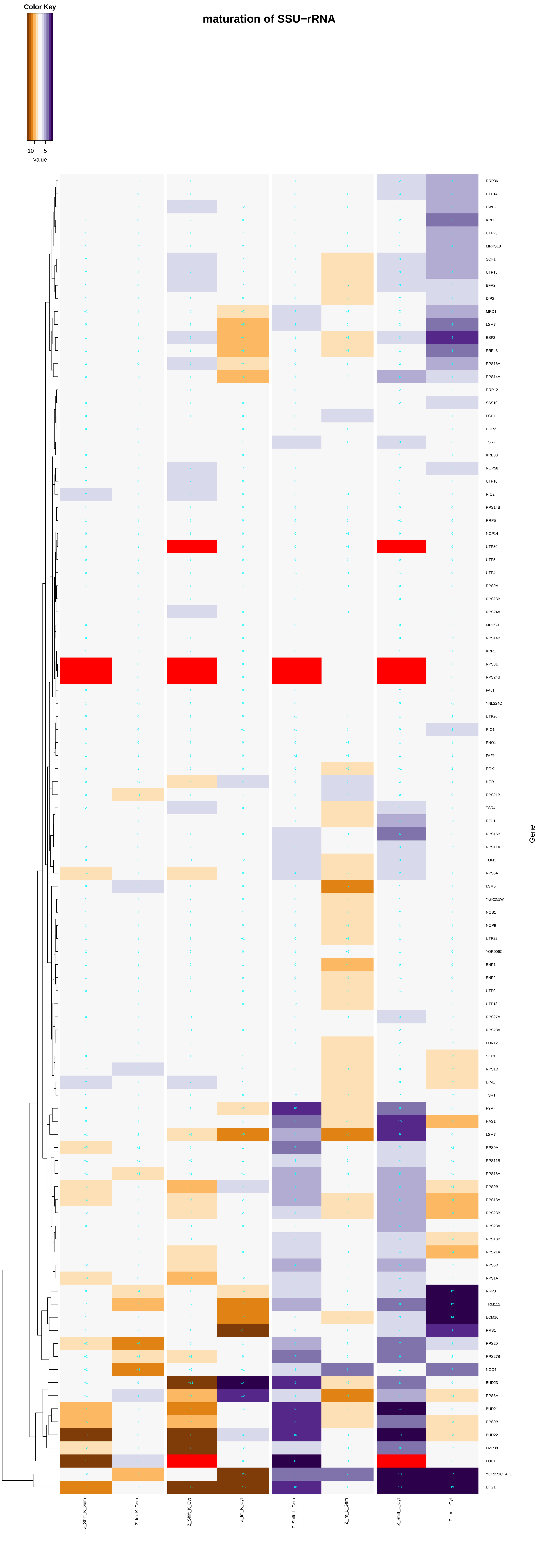
maturation of SSU−rRNA
Color Key
−10
5
Value
RRP36
UTP14
PWP2
KRI1
UTP23
MRPS18
SOF1
UTP15
BFR2
DIP2
MRD1
LSM7
ESF2
PRP43
RPS16A
RPS14A
RRP12
SAS10
FCF1
DHR2
TSR2
KRE33
NOP58
UTP10
RIO2
RPS14B
RRP5
NOP14
UTP30
UTP5
UTP4
RPS9A
RPS23B
RPS24A
MRPS9
RPS14B
KRR1
RPS31
RPS24B
FAL1
YNL224C
UTP20
RIO1
PNO1
FAF1
ROK1
HCR1
RPS21B
TSR4
RCL1
RPS16B
RPS11A
TOM1
RPS6A
LSM6
YGR251W
NOB1
NOP9
UTP22
YOR006C
ENP1
ENP2
UTP9
UTP13
RPS27A
RPS28A
FUN12
SLX9
RPS1B
DIM1
TSR1
FYV7
HAS1
LSM7
RPS0A
RPS11B
RPS16A
RPS9B
RPS18A
RPS28B
RPS23A
RPS18B
RPS21A
RPS6B
RPS1A
RRP3
TRM112
ECM16
RRS1
RPS20
RPS27B
NOC4
BUD23
RPS8A
BUD21
RPS0B
BUD22
FMP38
LOC1
YGR271C−A_1
EFG1
Gene
Z_Shift_K_Gem
Z_Im_K_Gem
Z_Shift_K_Cyt
Z_Im_K_Cyt
Z_Shift_L_Gem
Z_Im_L_Gem
Z_Shift_L_Cyt
Z_Im_L_Cyt

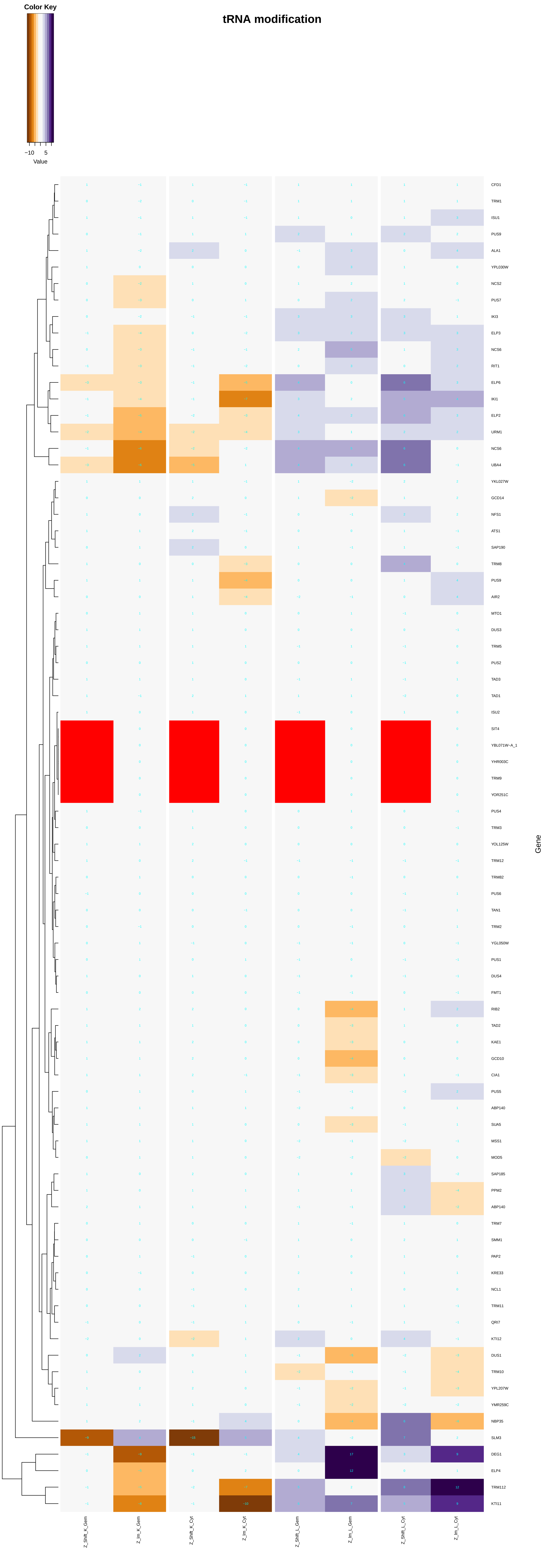
Color Key
−10
5
Value
tRNA modification
CFD1
TRM1
ISU1
PUS9
ALA1
YPL030W
NCS2
PUS7
IKI3
ELP3
NCS6
RIT1
ELP6
IKI1
ELP2
URM1
NCS6
UBA4
YKL027W
GCD14
NFS1
ATS1
SAP190
TRM8
PUS9
AIR2
MTO1
DUS3
TRM5
PUS2
TAD3
TAD1
ISU2
SIT4
YBL071W−A_1
YHR003C
TRM9
YOR251C
PUS4
TRM3
YOL125W
TRM12
TRM82
PUS6
TAN1
TRM2
YGL050W
PUS1
DUS4
FMT1
RIB2
TAD2
KAE1
GCD10
CIA1
PUS5
ABP140
SUA5
MSS1
MOD5
SAP185
PPM2
ABP140
TRM7
SMM1
PAP2
KRE33
NCL1
TRM11
QRI7
KTI12
DUS1
TRM10
YPL207W
YMR259C
NBP35
SLM3
DEG1
ELP4
TRM112
KTI11
Gene
Z_Shift_K_Gem
Z_Im_K_Gem
Z_Shift_K_Cyt
Z_Im_K_Cyt
Z_Shift_L_Gem
Z_Im_L_Gem
Z_Shift_L_Cyt
Z_Im_L_Cyt

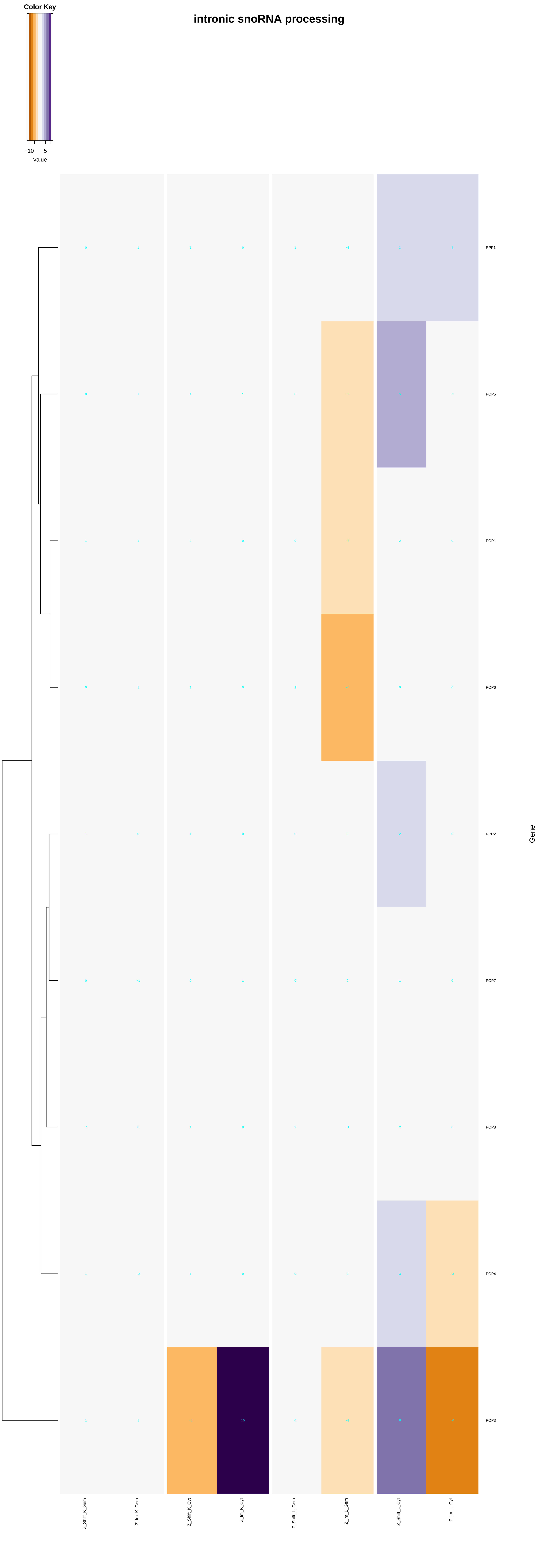
intronic snoRNA processing
Color Key
−10
5
Value
RPP1
POP5
POP1
POP6
RPR2
POP7
POP8
POP4
POP3
Gene
Z_Shift_K_Gem
Z_Im_K_Gem
Z_Shift_K_Cyt
Z_Im_K_Cyt
Z_Shift_L_Gem
Z_Im_L_Gem
Z_Shift_L_Cyt
Z_Im_L_Cyt

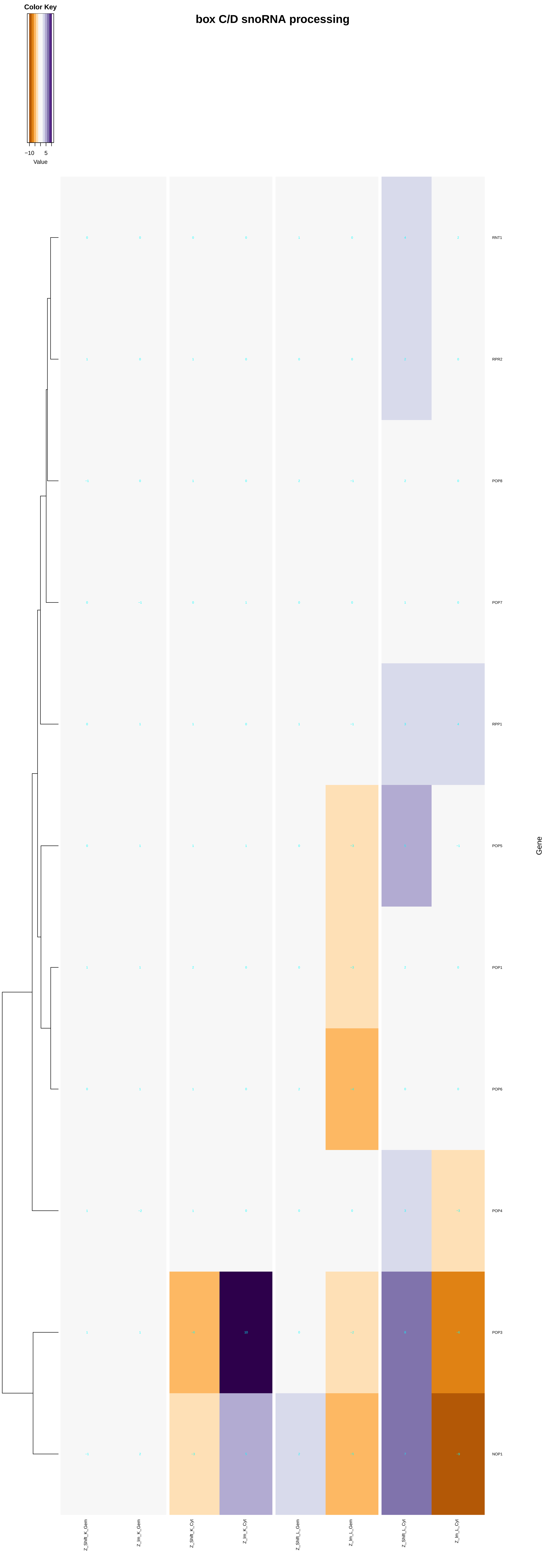
box C/D snoRNA processing
Color Key
−10
5
Value
RNT1
RPR2
POP8
POP7
RPP1
POP5
POP1
POP6
POP4
POP3
NOP1
Gene
Z_Shift_K_Gem
Z_Im_K_Gem
Z_Shift_K_Cyt
Z_Im_K_Cyt
Z_Shift_L_Gem
Z_Im_L_Gem
Z_Shift_L_Cyt
Z_Im_L_Cyt

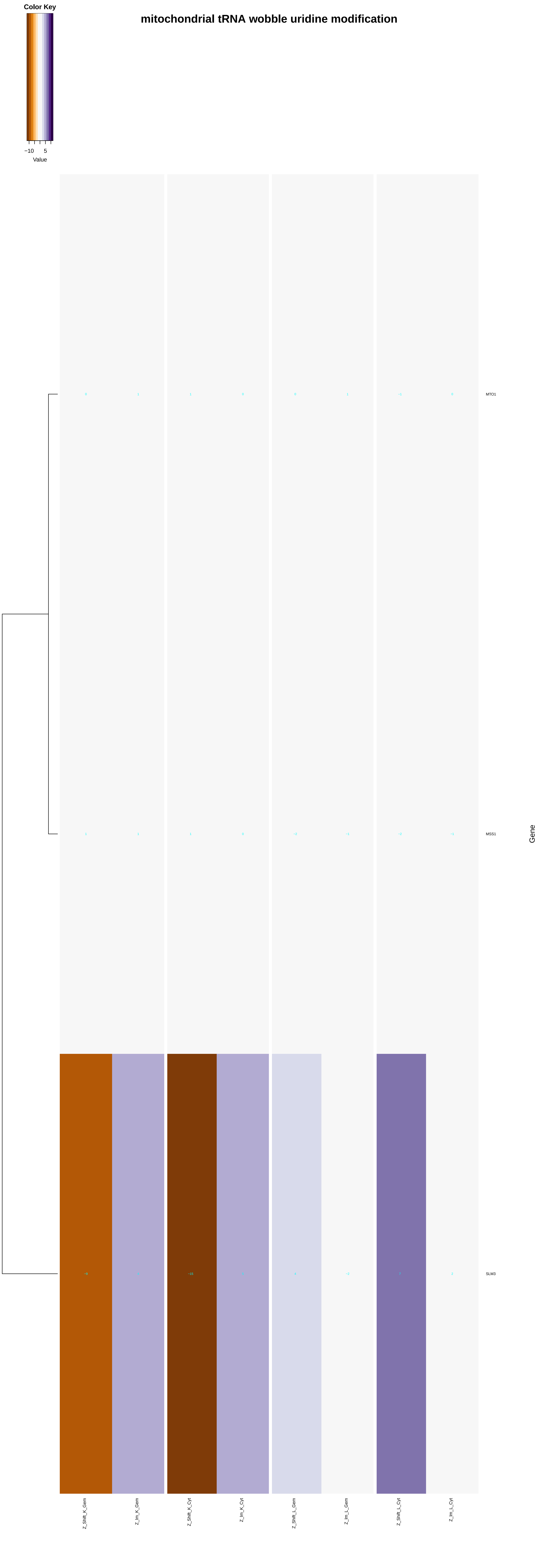

Color Key
−10
5
Value
mitochondrial tRNA wobble uridine modification
0
1
1
0
0
1
−1
0
MTO1
1
1
1
0
−2
−1
−2
−1
MSS1
−9
−15
4
−2
7
2
SLM3
Gene
Z_Shift_K_Gem
Z_Im_K_Gem
Z_Shift_K_Cyt
Z_Im_K_Cyt
Z_Shift_L_Gem
Z_Im_L_Gem
Z_Shift_L_Cyt
Z_Im_L_Cyt

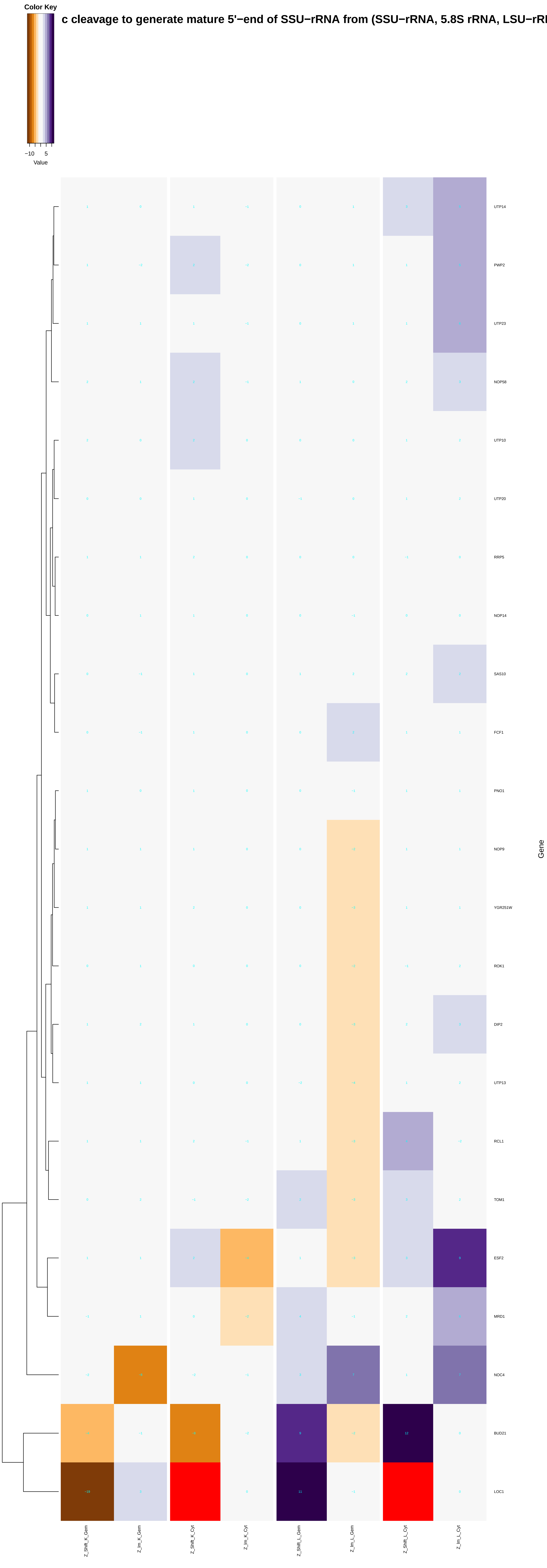
c cleavage to generate mature 5'−end of SSU−rRNA from (SSU−rRNA, 5.8S rRNA, LSU−rR
Color Key
−10
5
Value
Gene
UTP14
PWP2
UTP23
NOP58
UTP10
UTP20
RRP5
NOP14
SAS10
FCF1
PNO1
NOP9
YGR251W
ROK1
DIP2
UTP13
RCL1
TOM1
ESF2
MRD1
NOC4
BUD21
LOC1
Z_Shift_K_Gem
Z_Im_K_Gem
Z_Shift_K_Cyt
Z_Im_K_Cyt
Z_Shift_L_Gem
Z_Im_L_Gem
Z_Shift_L_Cyt
Z_Im_L_Cyt

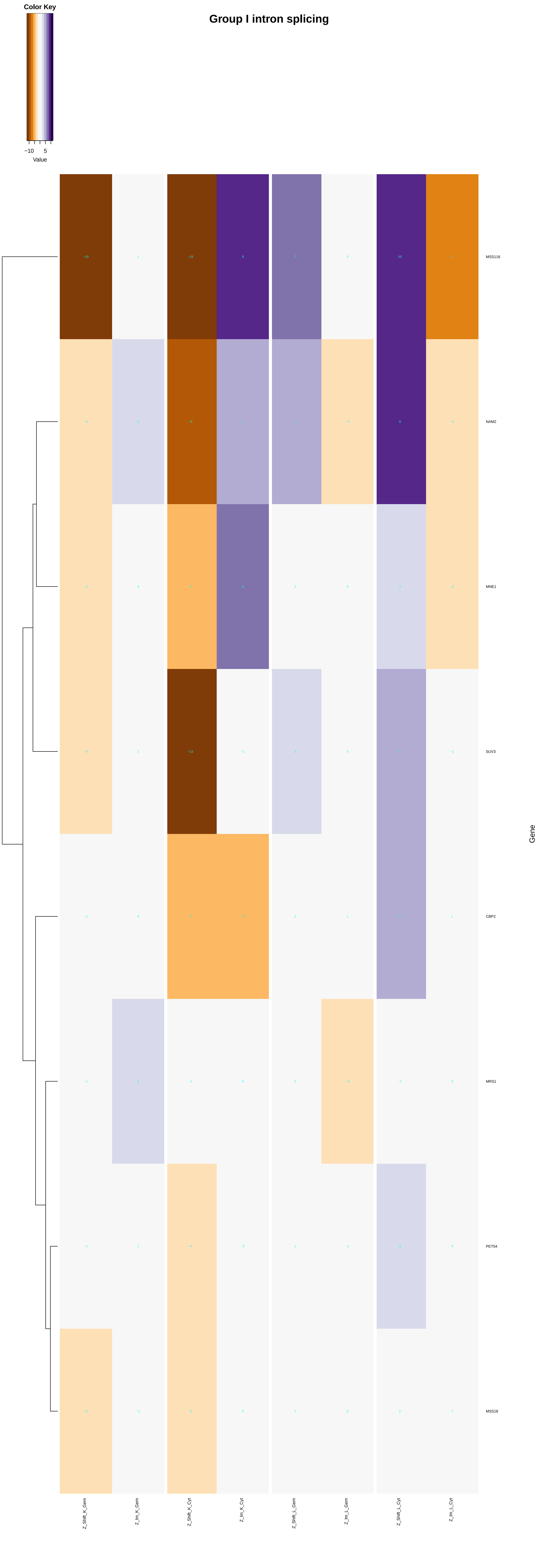
Group I intron splicing
Color Key
−10
5
Value
MSS116
NAM2
MNE1
SUV3
CBP2
MRS1
PET54
MSS18
Gene
Z_Shift_K_Gem
Z_Im_K_Gem
Z_Shift_K_Cyt
Z_Im_K_Cyt
Z_Shift_L_Gem
Z_Im_L_Gem
Z_Shift_L_Cyt
Z_Im_L_Cyt

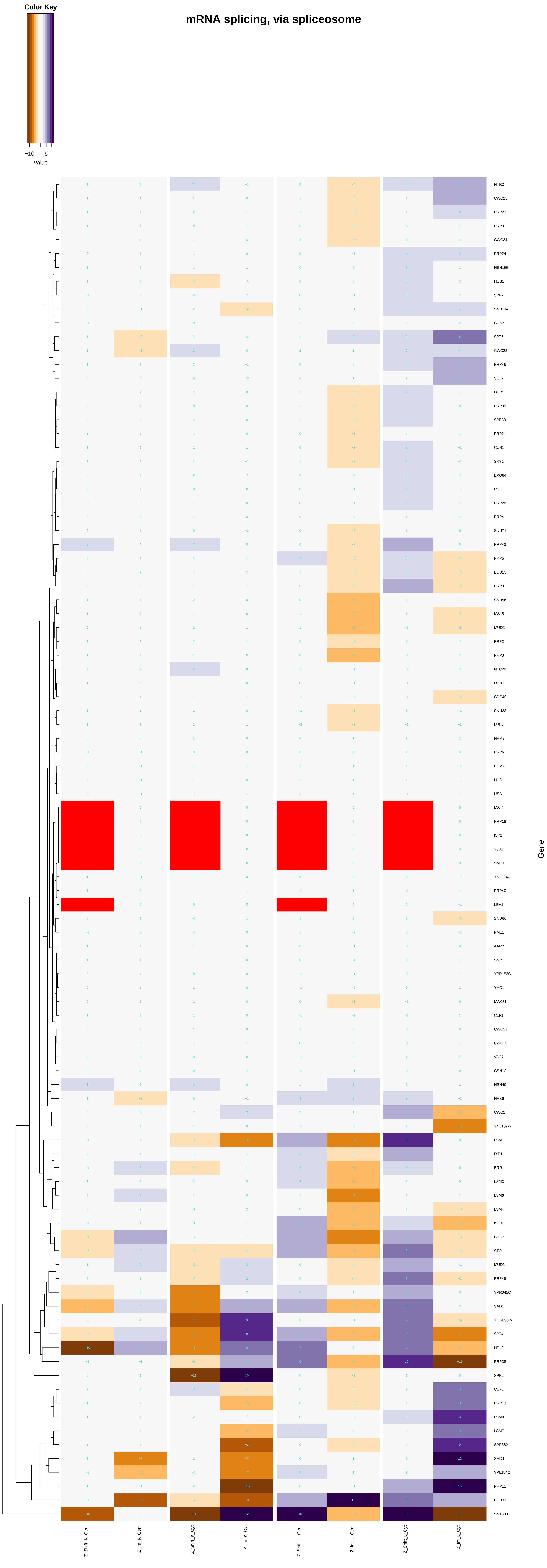

Color Key
−10
5
Value
mRNA splicing, via spliceosome
NTR2
CWC25
PRP22
PRP31
CWC24
PRP24
HSH155
HUB1
SYF2
SNU114
CUS2
SPT5
CWC22
PRP46
SLU7
DBR1
PRP39
SPP381
PRP21
CUS1
SKY1
EXO84
RSE1
PRP28
PRP4
SNU71
PRP42
PRP5
BUD13
PRP9
SNU56
MSL5
MUD2
PRP2
PRP3
NTC20
DED1
CDC40
SNU23
LUC7
NAM8
PRP6
ECM2
HUS1
USA1
MSL1
PRP18
ISY1
YJU2
SME1
YNL224C
PRP40
LEA1
SNU66
PML1
AAR2
SNP1
YPR152C
YHC1
MAK31
CLF1
CWC21
CWC15
VAC7
CSN12
HSH49
NAB6
CWC2
YNL187W
LSM7
DIB1
BRR1
LSM3
LSM6
LSM4
IST3
CBC2
STO1
MUD1
PRP45
YPR045C
SAD1
YGR093W
SPT4
NPL3
PRP38
SPP2
CEF1
PRP43
LSM8
LSM7
SPP382
SMD1
YPL184C
PRP11
BUD31
SNT309
Gene
Z_Shift_K_Gem
Z_Im_K_Gem
Z_Shift_K_Cyt
Z_Im_K_Cyt
Z_Shift_L_Gem
Z_Im_L_Gem
Z_Shift_L_Cyt
Z_Im_L_Cyt

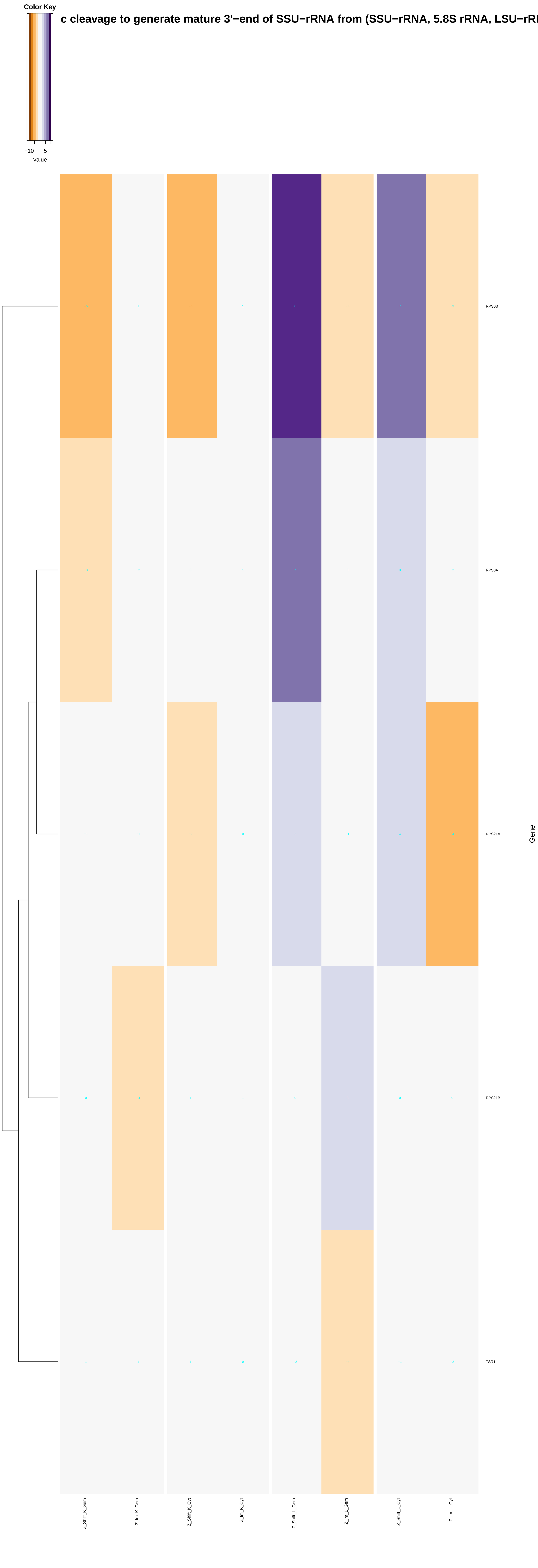
c cleavage to generate mature 3'−end of SSU−rRNA from (SSU−rRNA, 5.8S rRNA, LSU−rRI
Color Key
−10
5
Value
RPS0B
RPS0A
RPS21A
RPS21B
TSR1
Gene
Z_Shift_K_Gem
Z_Im_K_Gem
Z_Shift_K_Cyt
Z_Im_K_Cyt
Z_Shift_L_Gem
Z_Im_L_Gem
Z_Shift_L_Cyt
Z_Im_L_Cyt

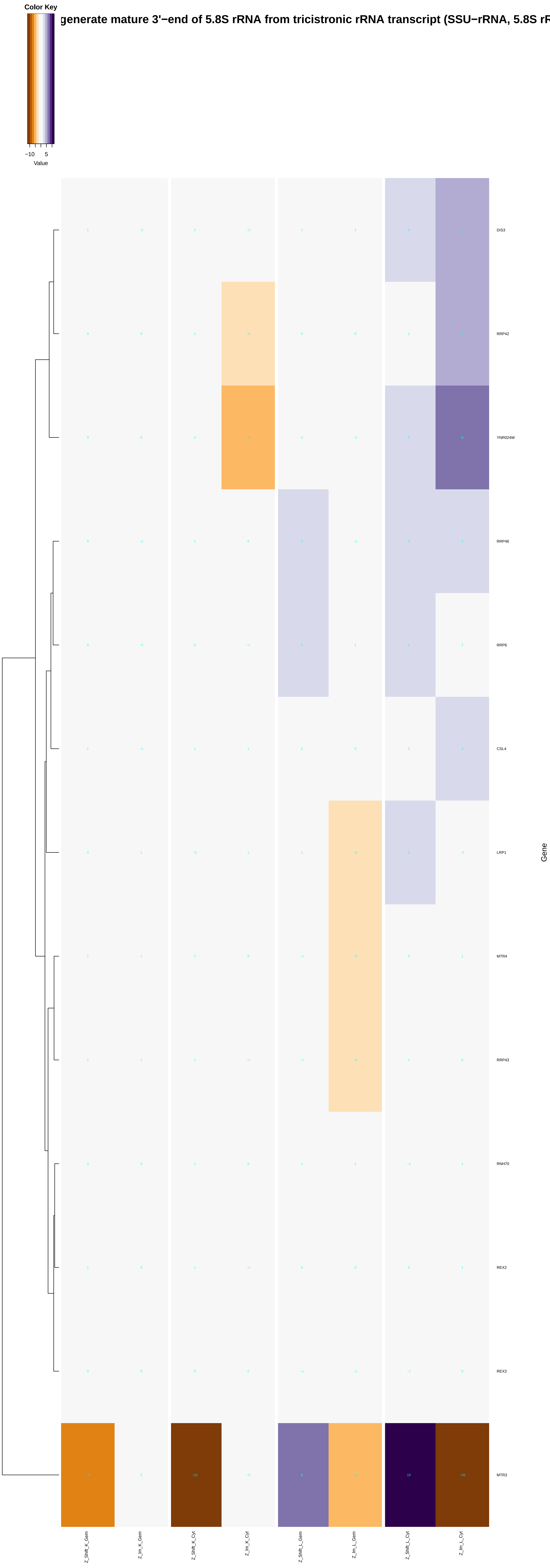

Color Key
−10
5
Value
generate mature 3'−end of 5.8S rRNA from tricistronic rRNA transcript (SSU−rRNA, 5.8S rR
DIS3
RRP42
YNR024W
RRP46
RRP6
CSL4
LRP1
MTR4
RRP43
RNH70
REX2
REX3
MTR3
Gene
Z_Shift_K_Gem
Z_Im_K_Gem
Z_Shift_K_Cyt
Z_Im_K_Cyt
Z_Shift_L_Gem
Z_Im_L_Gem
Z_Shift_L_Cyt
Z_Im_L_Cyt

snoRNA polyadenylation

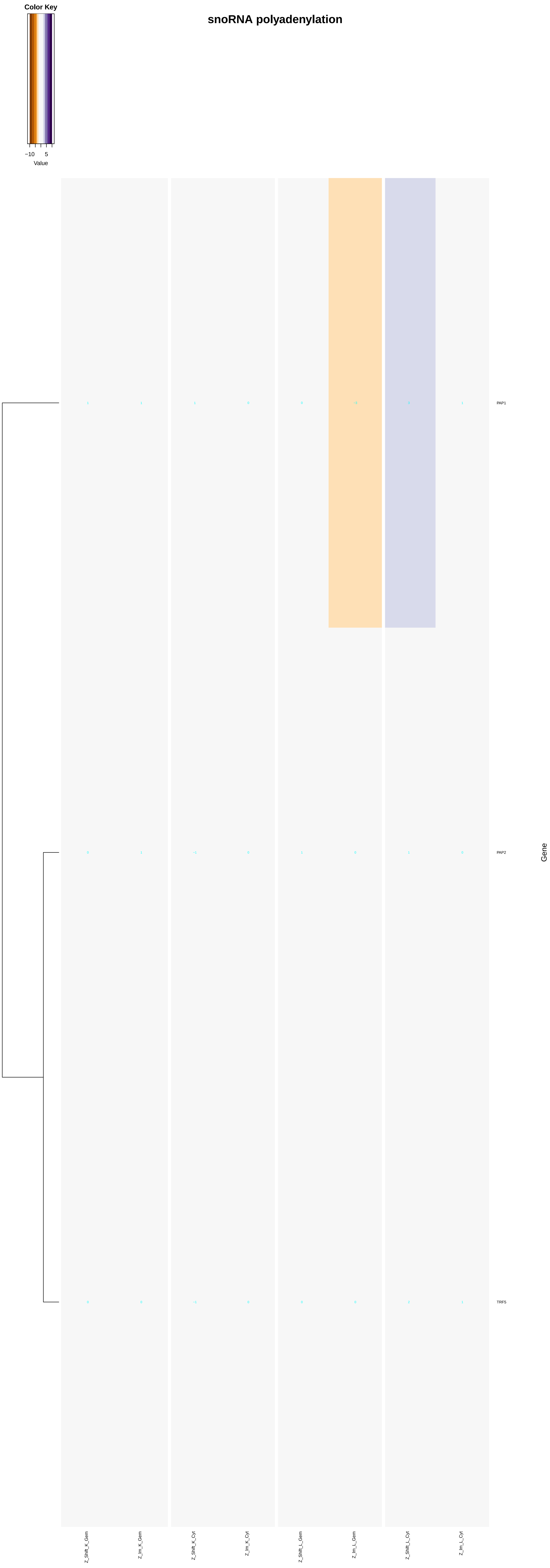

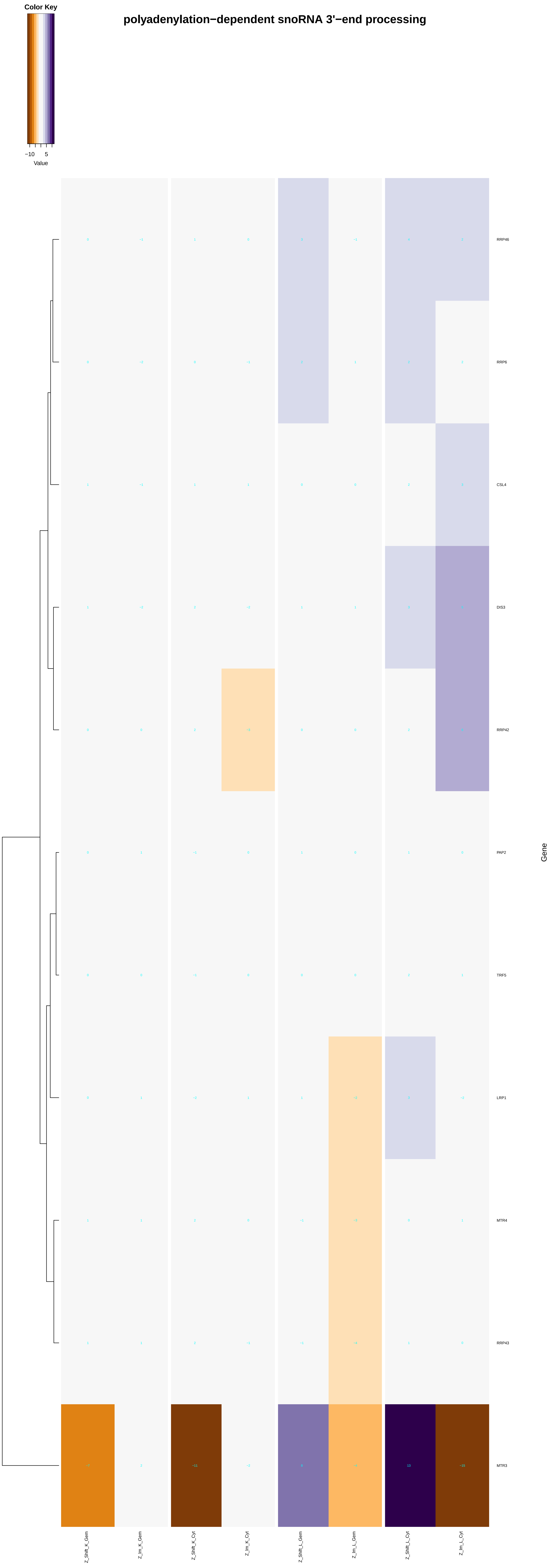

polyadenylation−dependent snoRNA 3'−end processing
Color Key
−10
5
Value
RRP46
RRP6
CSL4
DIS3
RRP42
PAP2
TRF5
LRP1
MTR4
RRP43
MTR3
Gene
Z_Shift_K_Gem
Z_Im_K_Gem
Z_Shift_K_Cyt
Z_Im_K_Cyt
Z_Shift_L_Gem
Z_Im_L_Gem
Z_Shift_L_Cyt
Z_Im_L_Cyt

U1 snRNA 3'−end processing

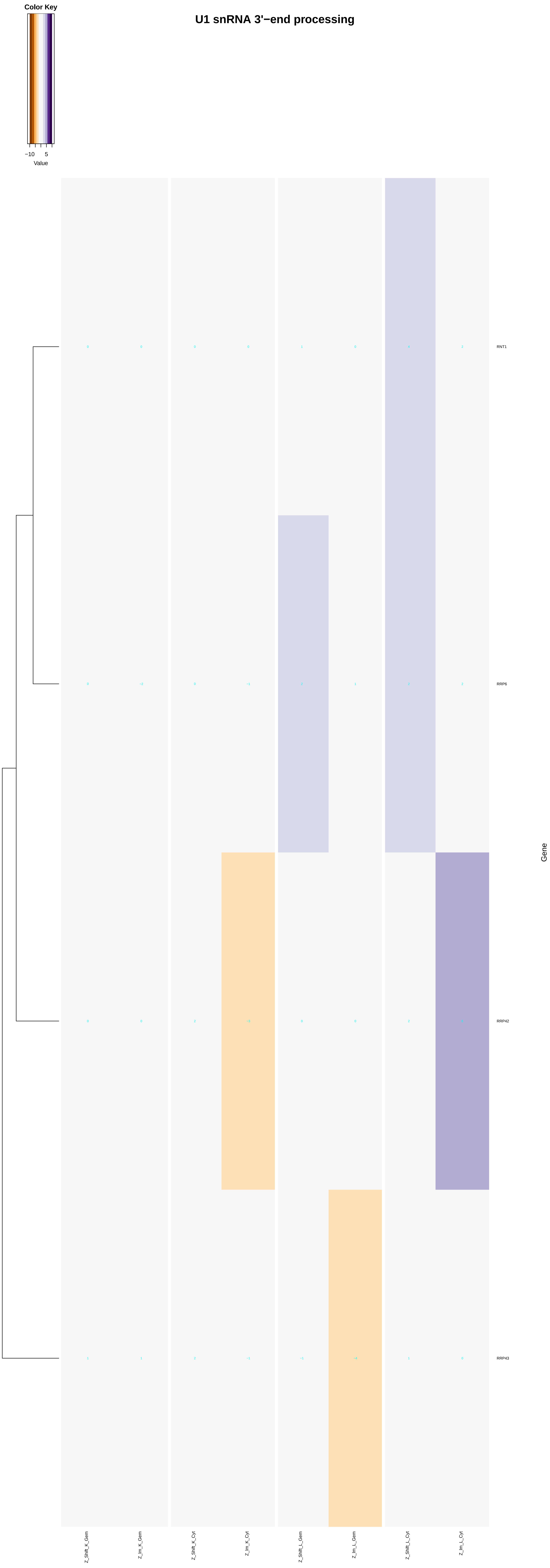

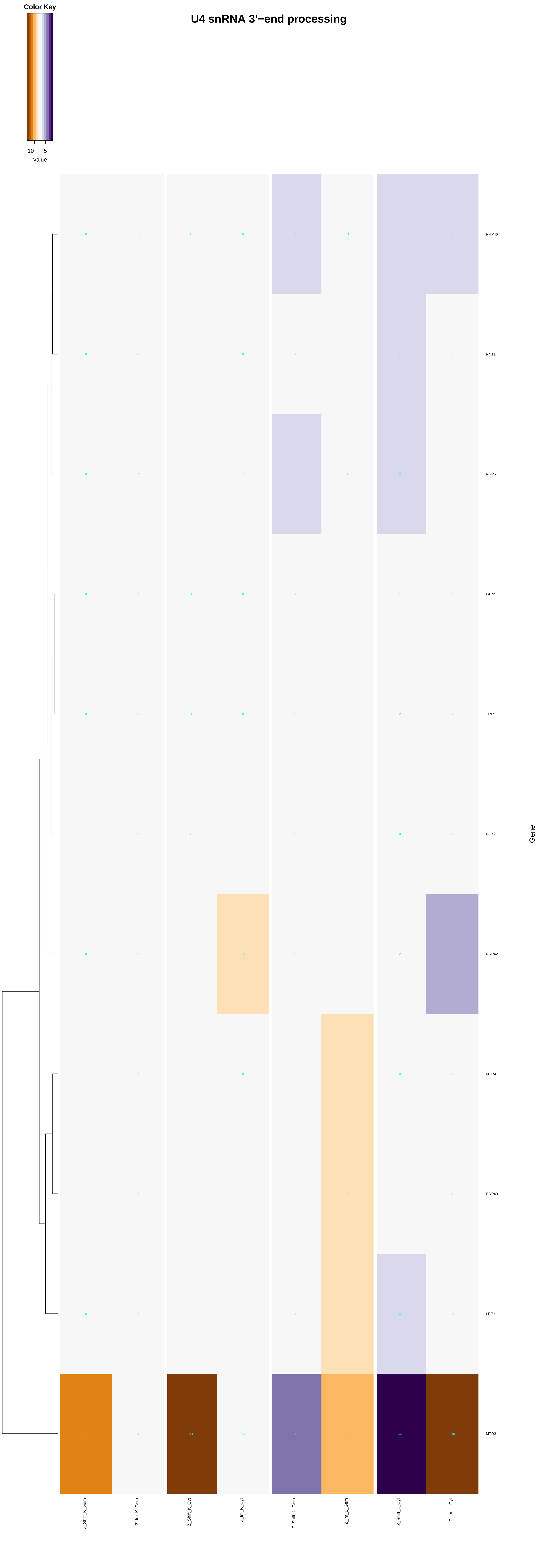

U4 snRNA 3'−end processing
Color Key
−10
5
Value
RRP46
RNT1
RRP6
PAP2
TRF5
REX2
RRP42
MTR4
RRP43
LRP1
MTR3
Gene
Z_Shift_K_Gem
Z_Im_K_Gem
Z_Shift_K_Cyt
Z_Im_K_Cyt
Z_Shift_L_Gem
Z_Im_L_Gem
Z_Shift_L_Cyt
Z_Im_L_Cyt

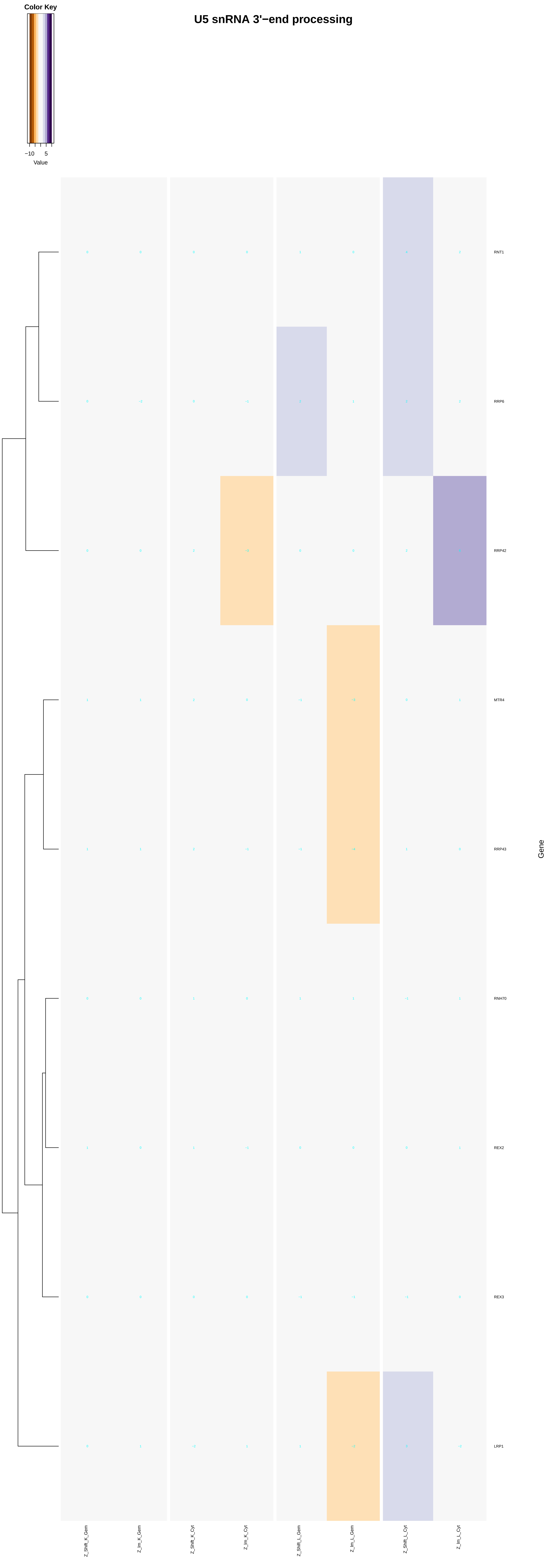
U5 snRNA 3'−end processing
Color Key
−10
5
Value
RNT1
RRP6
RRP42
MTR4
RRP43
RNH70
REX2
REX3
LRP1
Gene
Z_Shift_K_Gem
Z_Im_K_Gem
Z_Shift_K_Cyt
Z_Im_K_Cyt
Z_Shift_L_Gem
Z_Im_L_Gem
Z_Shift_L_Cyt
Z_Im_L_Cyt

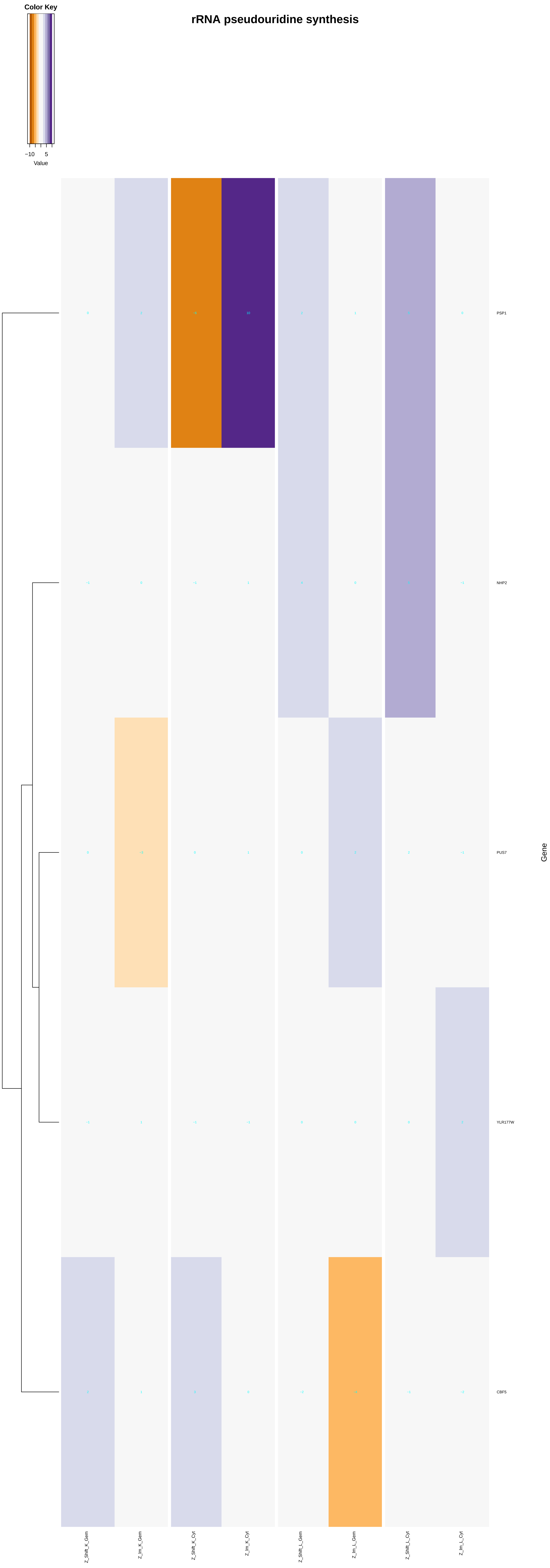
rRNA pseudouridine synthesis
Color Key
−10
5
Value
PSP1
NHP2
PUS7
YLR177W
CBF5
Gene
Z_Shift_K_Gem
Z_Im_K_Gem
Z_Shift_K_Cyt
Z_Im_K_Cyt
Z_Shift_L_Gem
Z_Im_L_Gem
Z_Shift_L_Cyt
Z_Im_L_Cyt

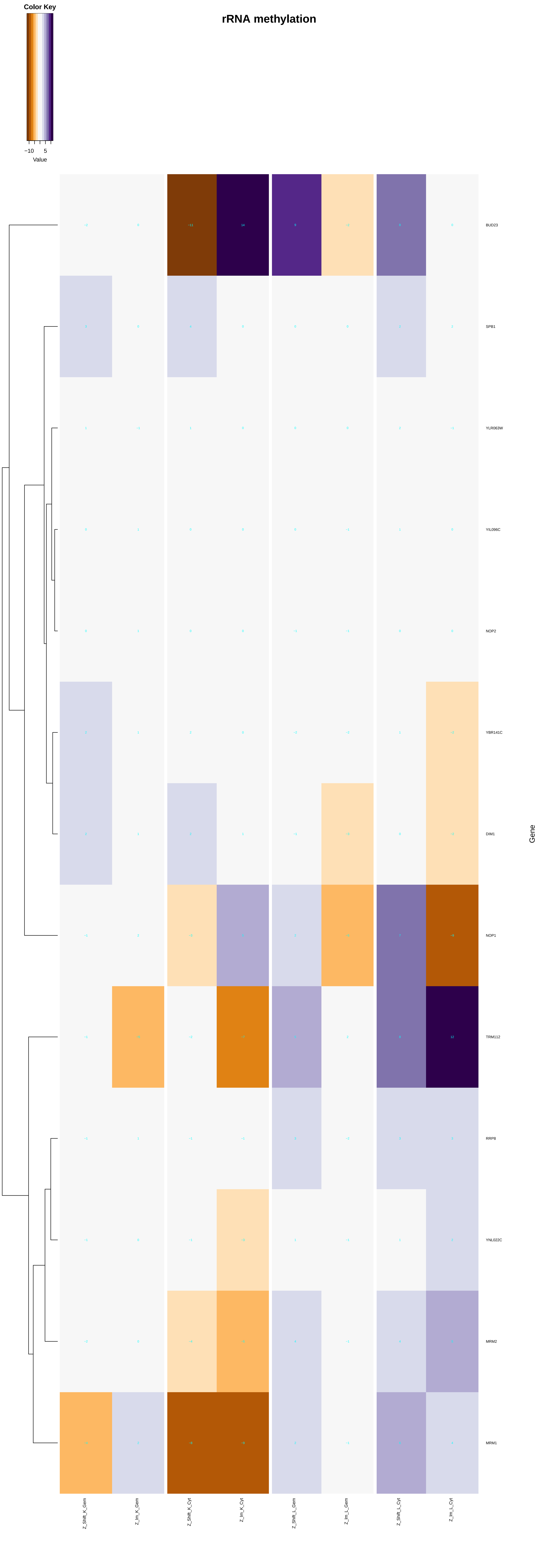
Color Key
−10
5
Value
rRNA methylation
BUD23
SPB1
YLR063W
YIL096C
NOP2
YBR141C
DIM1
NOP1
TRM112
RRP8
YNL022C
MRM2
MRM1
Gene
Z_Shift_K_Gem
Z_Im_K_Gem
Z_Shift_K_Cyt
Z_Im_K_Cyt
Z_Shift_L_Gem
Z_Im_L_Gem
Z_Shift_L_Cyt
Z_Im_L_Cyt

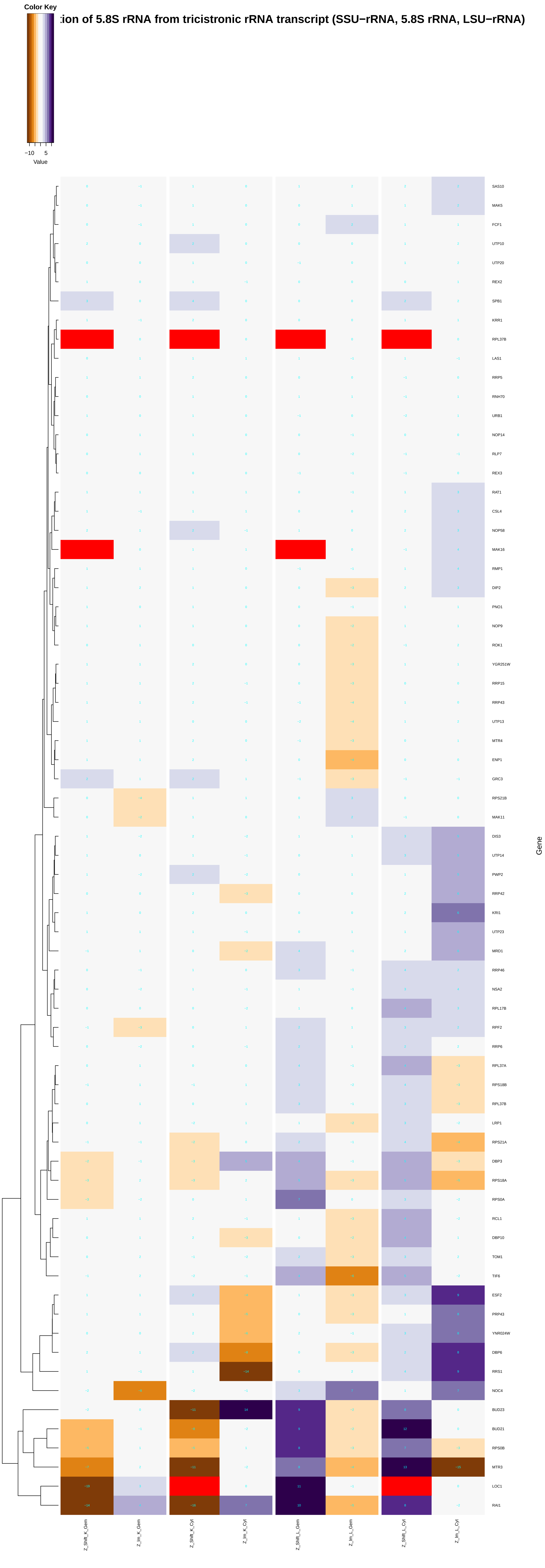

Color Key
−10
5
Value
:ion of 5.8S rRNA from tricistronic rRNA transcript (SSU−rRNA, 5.8S rRNA, LSU−rRNA)
SAS10
MAK5
FCF1
UTP10
UTP20
REX2
SPB1
KRR1
RPL37B
LAS1
RRP5
RNH70
URB1
NOP14
RLP7
REX3
RAT1
CSL4
NOP58
MAK16
RMP1
DIP2
PNO1
NOP9
ROK1
YGR251W
RRP15
RRP43
UTP13
MTR4
ENP1
GRC3
RPS21B
MAK11
DIS3
UTP14
PWP2
RRP42
KRI1
UTP23
MRD1
RRP46
NSA2
RPL17B
RPF2
RRP6
RPL37A
RPS18B
RPL37B
LRP1
RPS21A
DBP3
RPS18A
RPS0A
RCL1
DBP10
TOM1
TIF6
ESF2
PRP43
YNR024W
DBP6
RRS1
NOC4
BUD23
BUD21
RPS0B
MTR3
LOC1
RAI1
Gene
Z_Shift_K_Gem
Z_Im_K_Gem
Z_Shift_K_Cyt
Z_Im_K_Cyt
Z_Shift_L_Gem
Z_Im_L_Gem
Z_Shift_L_Cyt
Z_Im_L_Cyt

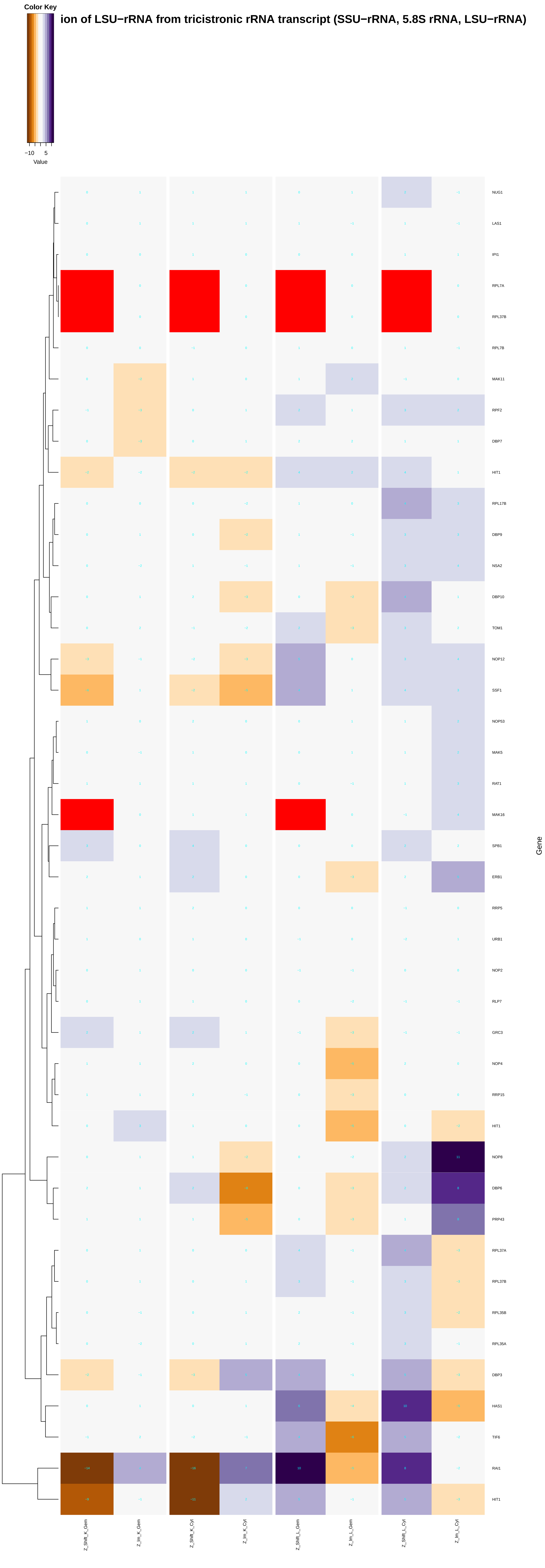
ion of LSU−rRNA from tricistronic rRNA transcript (SSU−rRNA, 5.8S rRNA, LSU−rRNA)
Color Key
−10
5
Value
NUG1
LAS1
IPI1
RPL7A
RPL37B
RPL7B
MAK11
RPF2
DBP7
HIT1
RPL17B
DBP9
NSA2
DBP10
TOM1
NOP12
SSF1
NOP53
MAK5
RAT1
MAK16
SPB1
ERB1
RRP5
URB1
NOP2
RLP7
GRC3
NOP4
RRP15
HIT1
NOP8
DBP6
PRP43
RPL37A
RPL37B
RPL35B
RPL35A
DBP3
HAS1
TIF6
RAI1
HIT1
Gene
Z_Shift_K_Gem
Z_Im_K_Gem
Z_Shift_K_Cyt
Z_Im_K_Cyt
Z_Shift_L_Gem
Z_Im_L_Gem
Z_Shift_L_Cyt
Z_Im_L_Cyt

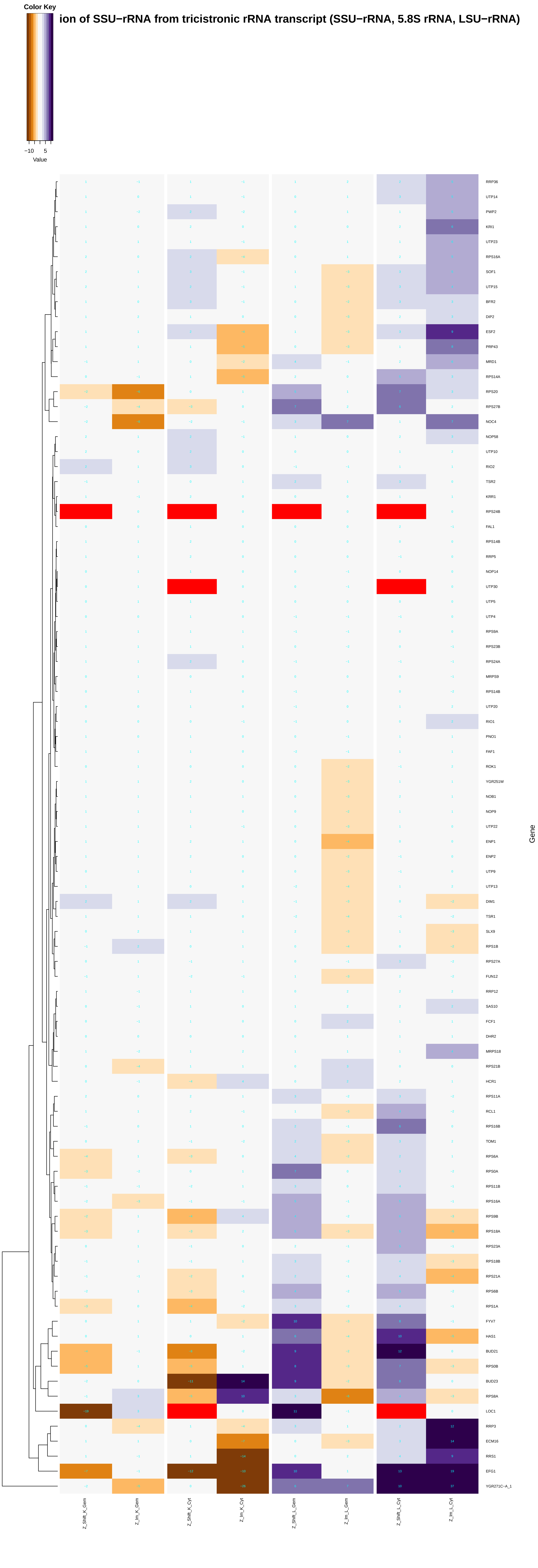
ion of SSU−rRNA from tricistronic rRNA transcript (SSU−rRNA, 5.8S rRNA, LSU−rRNA)
Color Key
−10
5
Value
RRP36
UTP14
PWP2
KRI1
UTP23
RPS16A
SOF1
UTP15
BFR2
DIP2
ESF2
PRP43
MRD1
RPS14A
RPS20
RPS27B
NOC4
NOP58
UTP10
RIO2
TSR2
KRR1
RPS24B
FAL1
RPS14B
RRP5
NOP14
UTP30
UTP5
UTP4
RPS9A
RPS23B
RPS24A
MRPS9
RPS14B
UTP20
RIO1
PNO1
FAF1
ROK1
YGR251W
NOB1
NOP9
UTP22
ENP1
ENP2
UTP9
UTP13
DIM1
TSR1
SLX9
RPS1B
RPS27A
FUN12
RRP12
SAS10
FCF1
DHR2
MRPS18
RPS21B
HCR1
RPS11A
RCL1
RPS16B
TOM1
RPS6A
RPS0A
RPS11B
RPS16A
RPS9B
RPS18A
RPS23A
RPS18B
RPS21A
RPS6B
RPS1A
FYV7
HAS1
BUD21
RPS0B
BUD23
RPS8A
LOC1
RRP3
ECM16
RRS1
EFG1
YGR271C−A_1
Gene
Z_Shift_K_Gem
Z_Im_K_Gem
Z_Shift_K_Cyt
Z_Im_K_Cyt
Z_Shift_L_Gem
Z_Im_L_Gem
Z_Shift_L_Cyt
Z_Im_L_Cyt

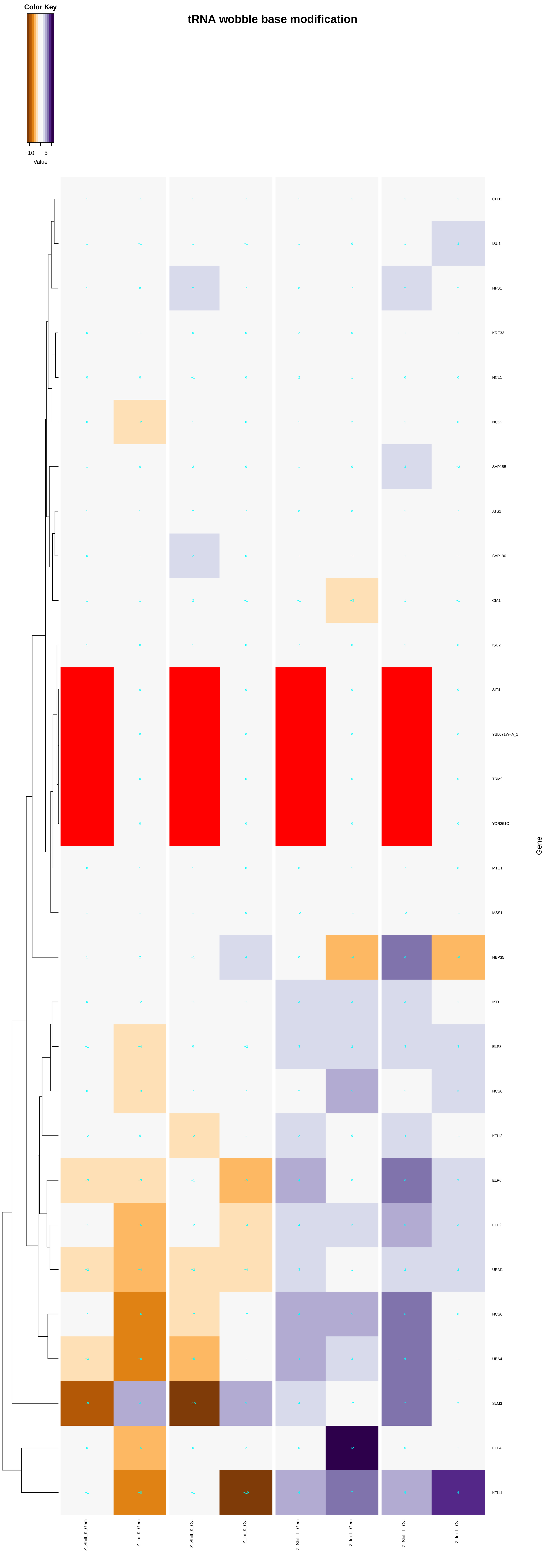
tRNA wobble base modification
Color Key
−10
5
Value
CFD1
ISU1
NFS1
KRE33
NCL1
NCS2
SAP185
ATS1
SAP190
CIA1
ISU2
SIT4
YBL071W−A_1
TRM9
YOR251C
MTO1
MSS1
NBP35
IKI3
ELP3
NCS6
KTI12
ELP6
ELP2
URM1
NCS6
UBA4
SLM3
ELP4
KTI11
Gene
Z_Shift_K_Gem
Z_Im_K_Gem
Z_Shift_K_Cyt
Z_Im_K_Cyt
Z_Shift_L_Gem
Z_Im_L_Gem
Z_Shift_L_Cyt
Z_Im_L_Cyt

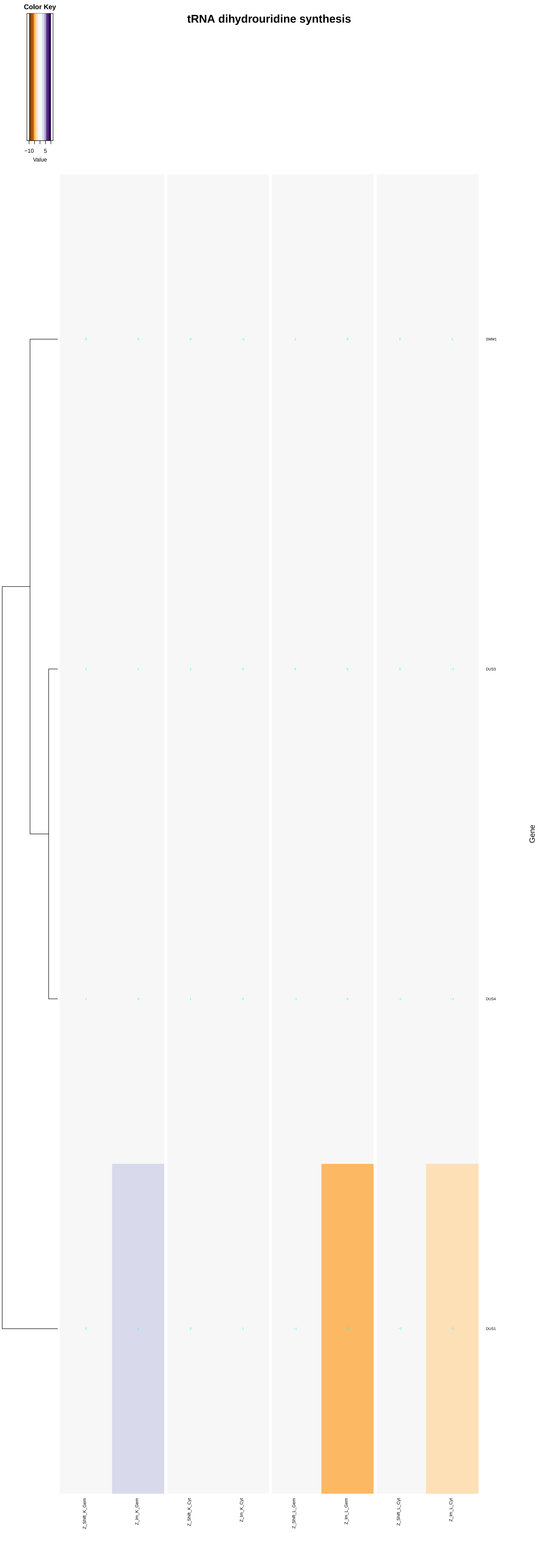

Color Key
−10
5
Value
tRNA dihydrouridine synthesis
SMM1
DUS3
DUS4
DUS1
Gene
Z_Shift_K_Gem
Z_Im_K_Gem
Z_Shift_K_Cyt
Z_Im_K_Cyt
Z_Shift_L_Gem
Z_Im_L_Gem
Z_Shift_L_Cyt
Z_Im_L_Cyt

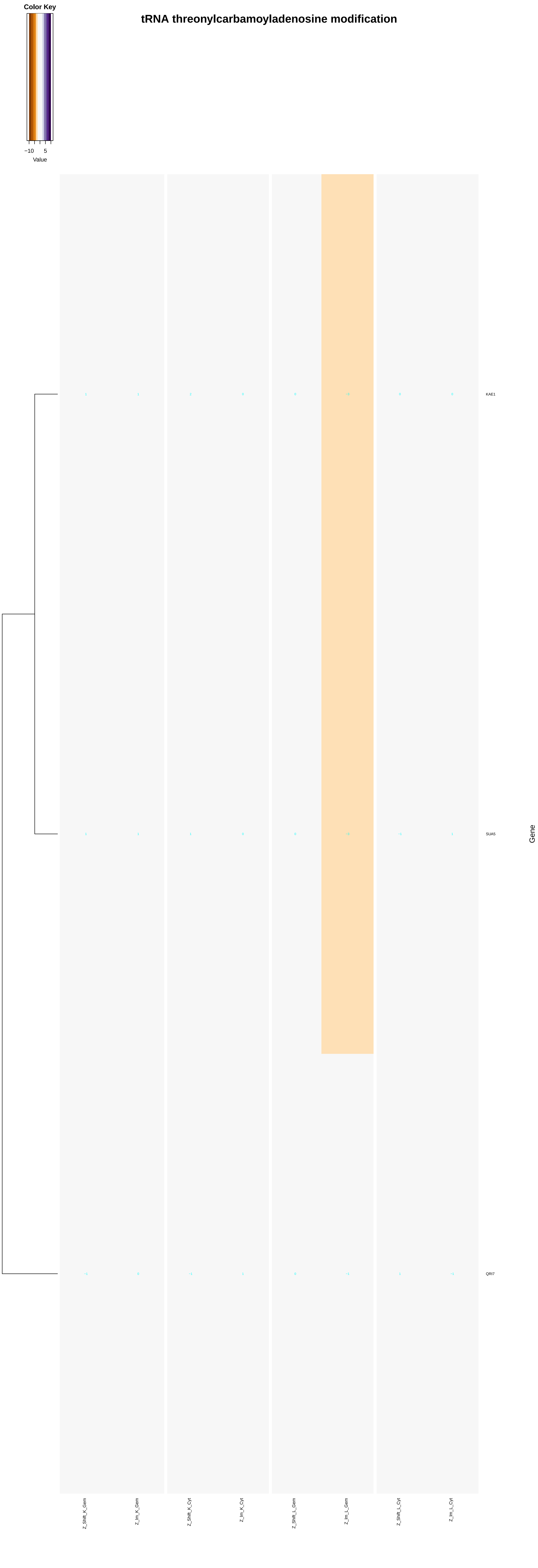

tRNA threonylcarbamoyladenosine modification
Color Key
−10
5
Value
KAE1
SUA5
QRI7
Gene
Z_Shift_K_Gem
Z_Im_K_Gem
Z_Shift_K_Cyt
Z_Im_K_Cyt
Z_Shift_L_Gem
Z_Im_L_Gem
Z_Shift_L_Cyt
Z_Im_L_Cyt

tRNA methylation

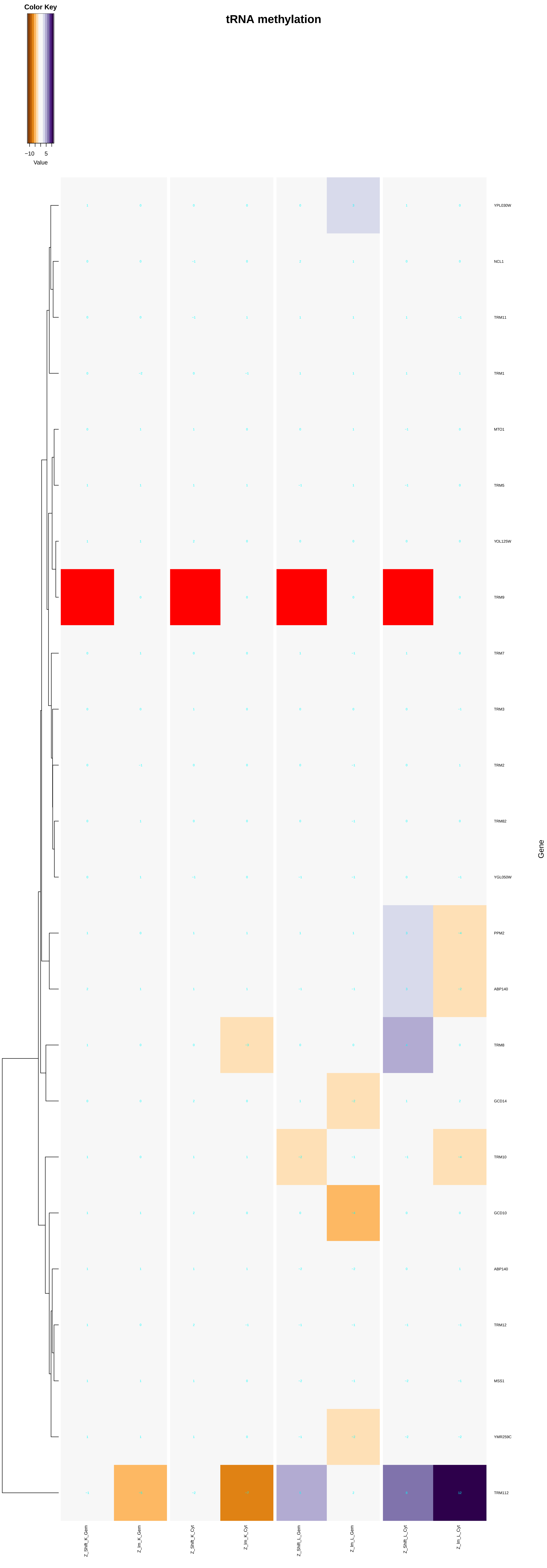

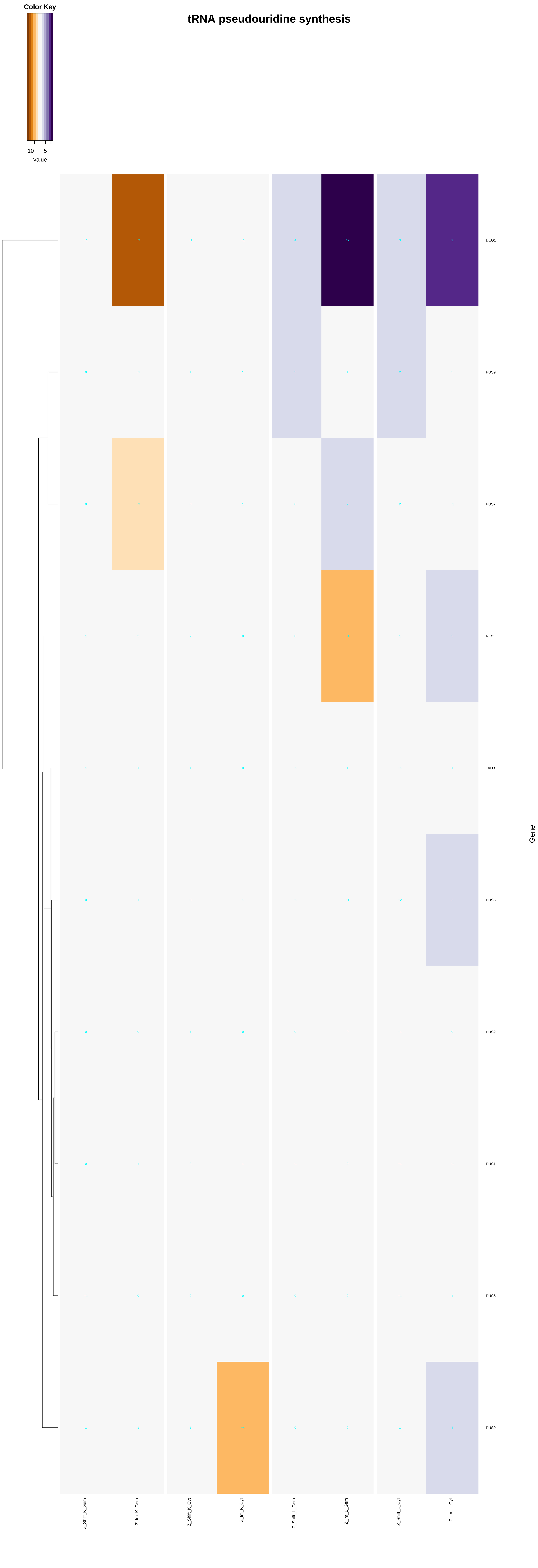

Color Key
−10
5
Value
tRNA pseudouridine synthesis
DEG1
PUS9
PUS7
RIB2
TAD3
PUS5
PUS2
PUS1
PUS6
PUS9
Gene
Z_Shift_K_Gem
Z_Im_K_Gem
Z_Shift_K_Cyt
Z_Im_K_Cyt
Z_Shift_L_Gem
Z_Im_L_Gem
Z_Shift_L_Cyt
Z_Im_L_Cyt

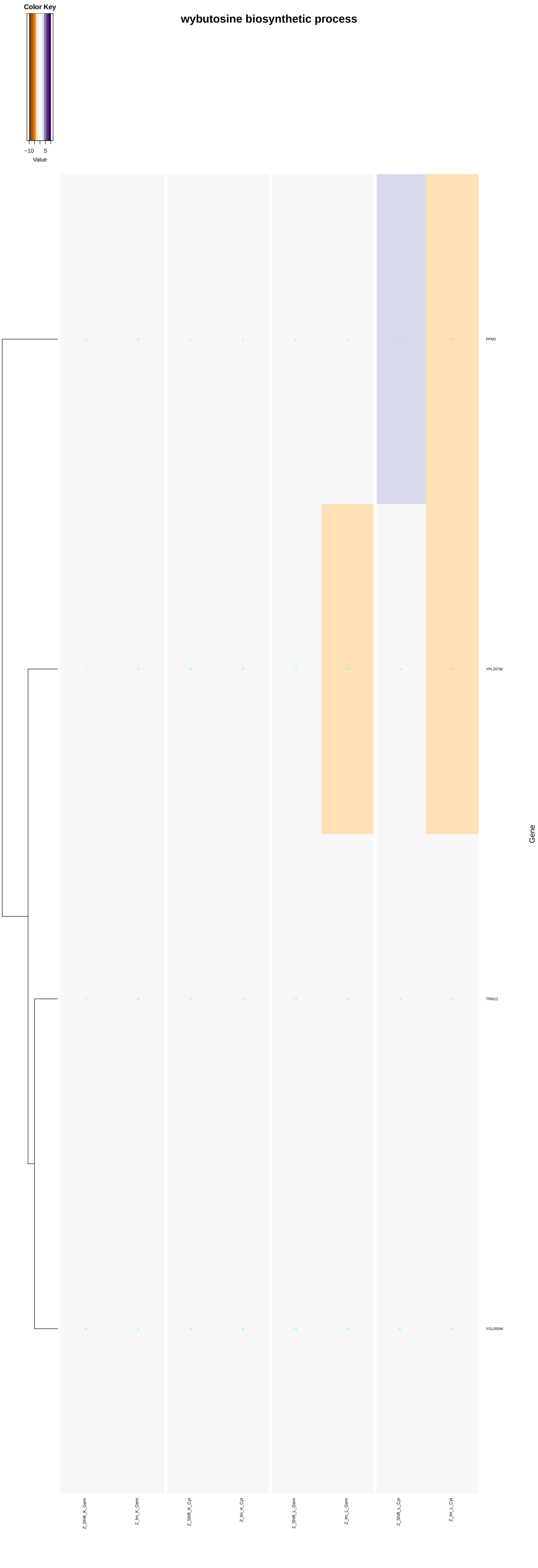
wybutosine biosynthetic process
Color Key
−10
5
Value
PPM2
YPL207W
TRM12
YGL050W
Gene
Z_Shift_K_Gem
Z_Im_K_Gem
Z_Shift_K_Cyt
Z_Im_K_Cyt
Z_Shift_L_Gem
Z_Im_L_Gem
Z_Shift_L_Cyt
Z_Im_L_Cyt

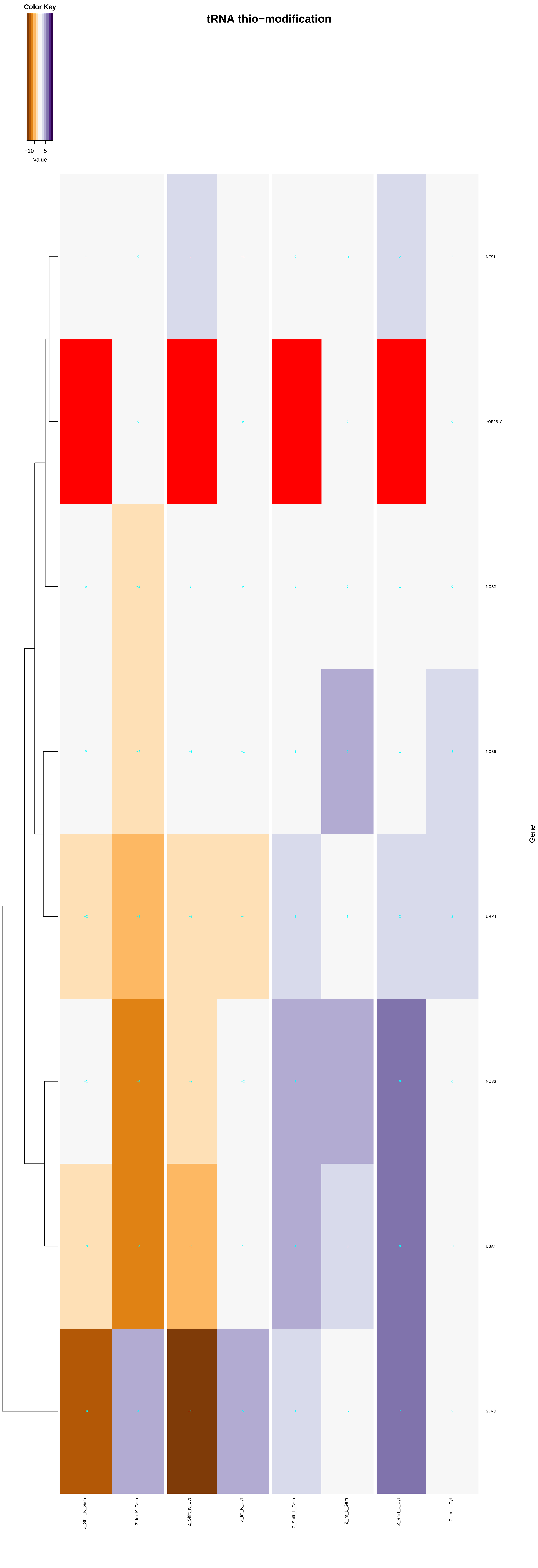
tRNA thio−modification
Color Key
−10
5
Value
NFS1
YOR251C
NCS2
NCS6
URM1
NCS6
UBA4
SLM3
Gene
Z_Shift_K_Gem
Z_Im_K_Gem
Z_Shift_K_Cyt
Z_Im_K_Cyt
Z_Shift_L_Gem
Z_Im_L_Gem
Z_Shift_L_Cyt
Z_Im_L_Cyt

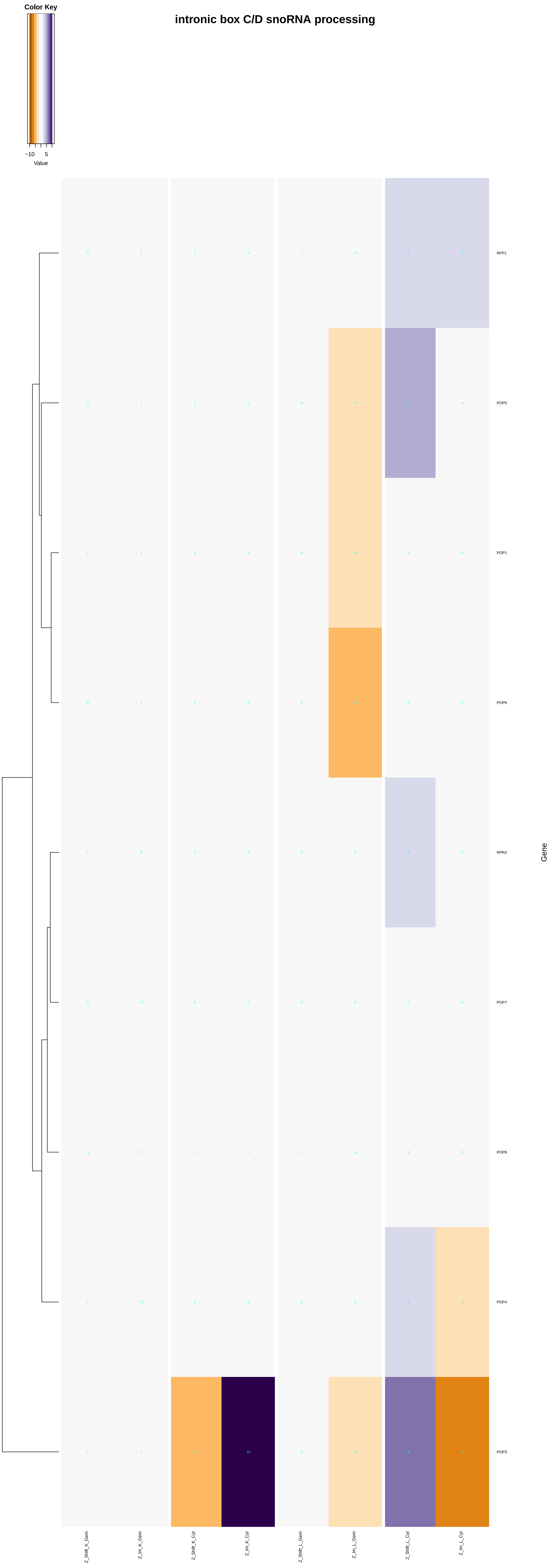
Color Key
−10
5
Value
intronic box C/D snoRNA processing
RPP1
POP5
POP1
POP6
RPR2
POP7
POP8
POP4
POP3
Gene
Z_Shift_K_Gem
Z_Im_K_Gem
Z_Shift_K_Cyt
Z_Im_K_Cyt
Z_Shift_L_Gem
Z_Im_L_Gem
Z_Shift_L_Cyt
Z_Im_L_Cyt

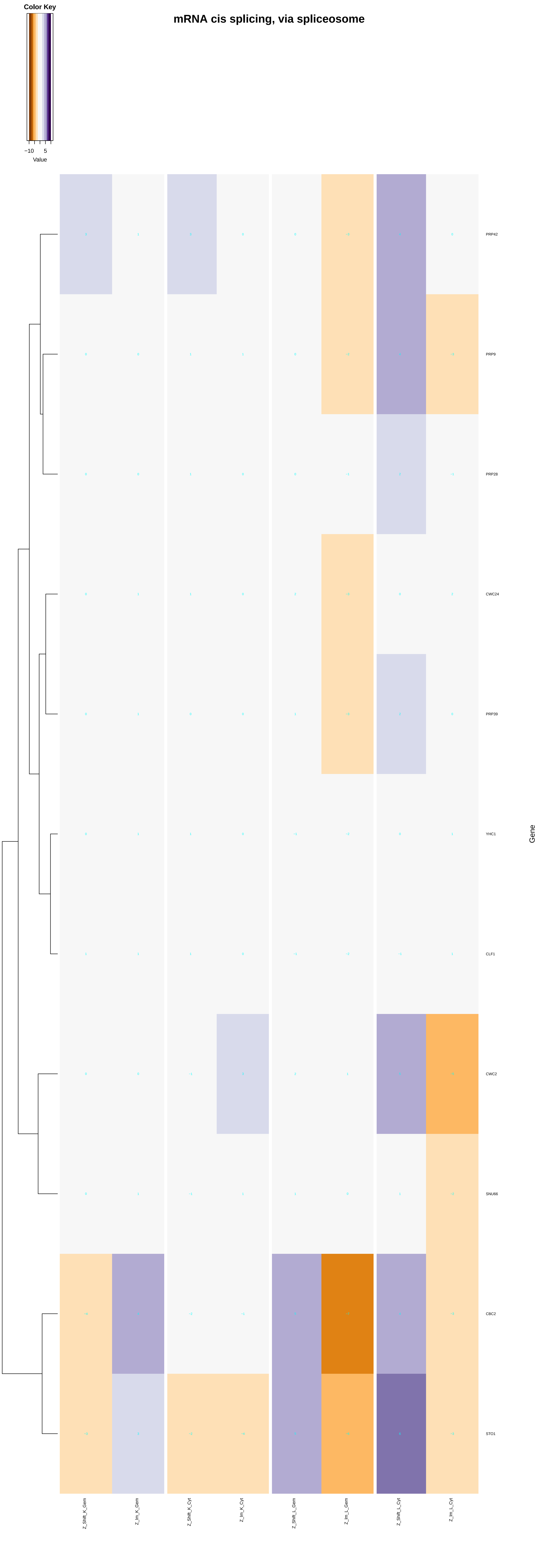
Color Key
−10
5
Value
mRNA cis splicing, via spliceosome
PRP42
PRP9
PRP28
CWC24
PRP39
YHC1
CLF1
CWC2
SNU66
CBC2
STO1
Gene
Z_Shift_K_Gem
Z_Im_K_Gem
Z_Shift_K_Cyt
Z_Im_K_Cyt
Z_Shift_L_Gem
Z_Im_L_Gem
Z_Shift_L_Cyt
Z_Im_L_Cyt

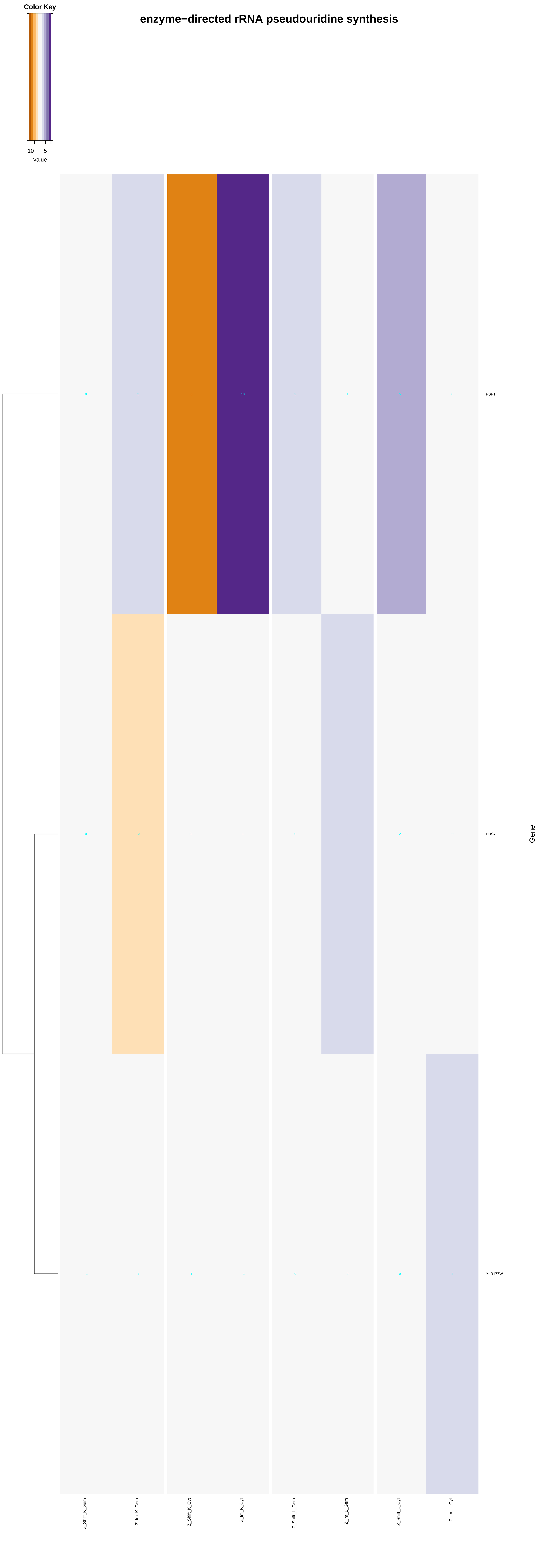

enzyme−directed rRNA pseudouridine synthesis
Color Key
−10
5
Value
PSP1
PUS7
YLR177W
Gene
Z_Shift_K_Gem
Z_Im_K_Gem
Z_Shift_K_Cyt
Z_Im_K_Cyt
Z_Shift_L_Gem
Z_Im_L_Gem
Z_Shift_L_Cyt
Z_Im_L_Cyt

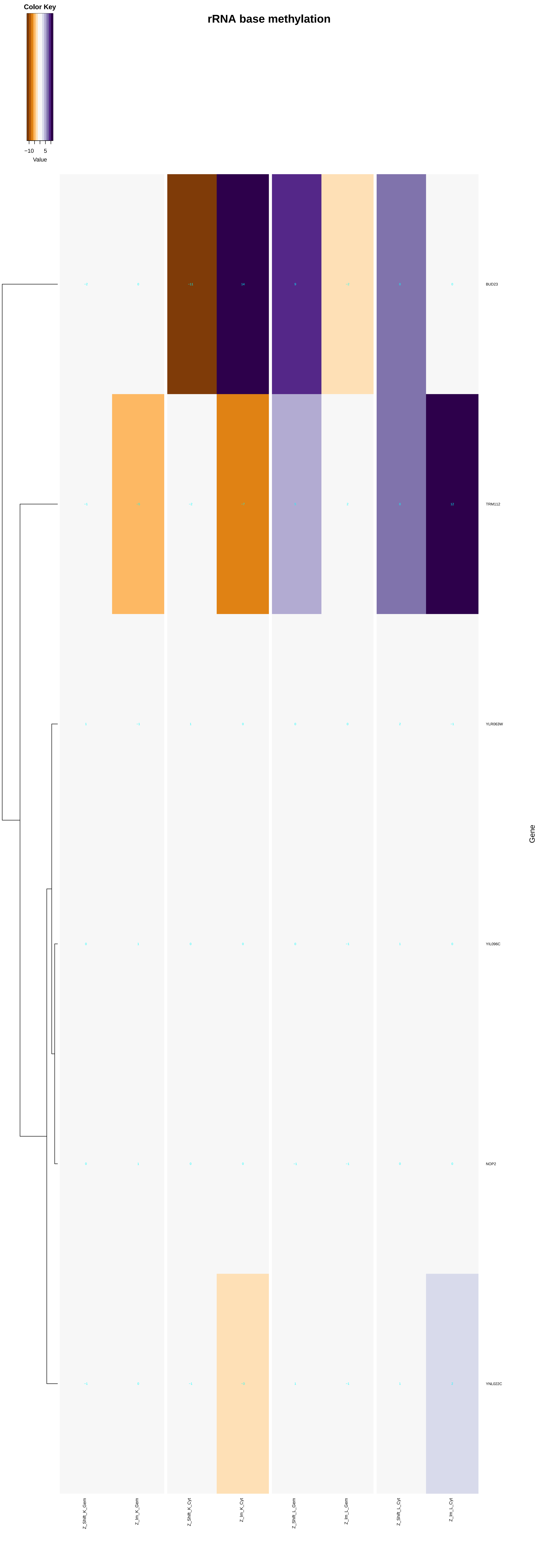

rRNA base methylation
Color Key
−10
5
Value
BUD23
TRM112
YLR063W
YIL096C
NOP2
YNL022C
Gene
Z_Shift_K_Gem
Z_Int_K_Gem
Z_Shift_K_Cyt
Z_Int_K_Cyt
Z_Shift_L_Gem
Z_Int_L_Gem
Z_Shift_L_Cyt
Z_Int_L_Cyt

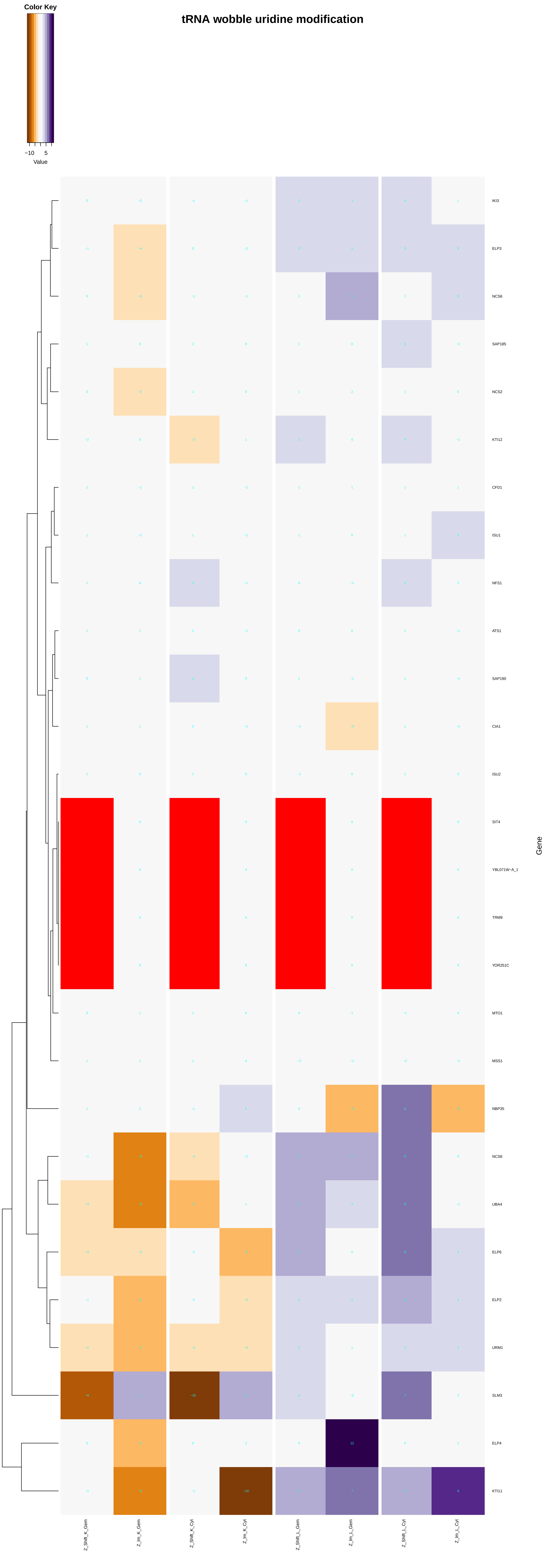
tRNA wobble uridine modification
Color Key
−10
5
Value
IKI3
ELP3
NCS6
SAP185
NCS2
KTI12
CFD1
ISU1
NFS1
ATS1
SAP190
CIA1
ISU2
SIT4
YBL071W−A_1
TRM9
YOR251C
MTO1
MSS1
NBP35
NCS6
UBA4
ELP6
ELP2
URM1
SLM3
ELP4
KTI11
Gene
Z_Shift_K_Gem
Z_Im_K_Gem
Z_Shift_K_Cyt
Z_Im_K_Cyt
Z_Shift_L_Gem
Z_Im_L_Gem
Z_Shift_L_Cyt
Z_Im_L_Cyt

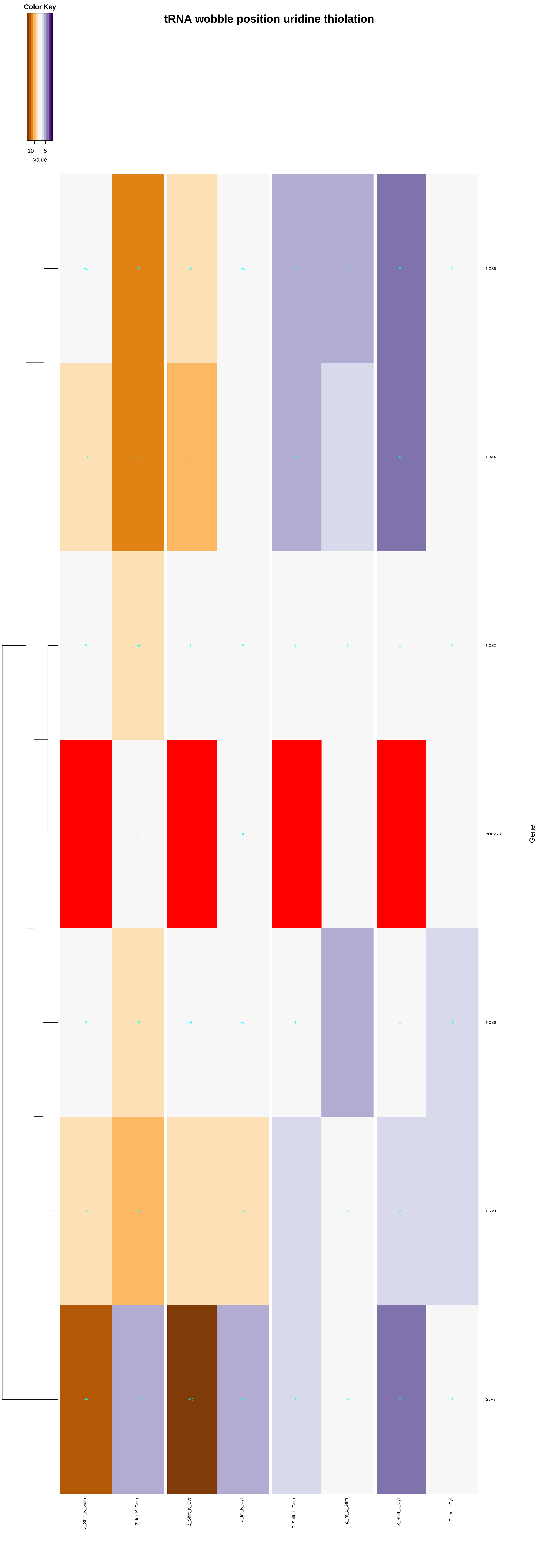

tRNA wobble position uridine thiolation
Color Key
−10
5
Value
NCS6
UBA4
NCS2
YOR251C
NCS6
URM1
SLM3
Gene
Z_Shift_K_Gem
Z_Im_K_Gem
Z_Shift_K_Cyt
Z_Im_K_Cyt
Z_Shift_L_Gem
Z_Im_L_Gem
Z_Shift_L_Cyt
Z_Im_L_Cyt

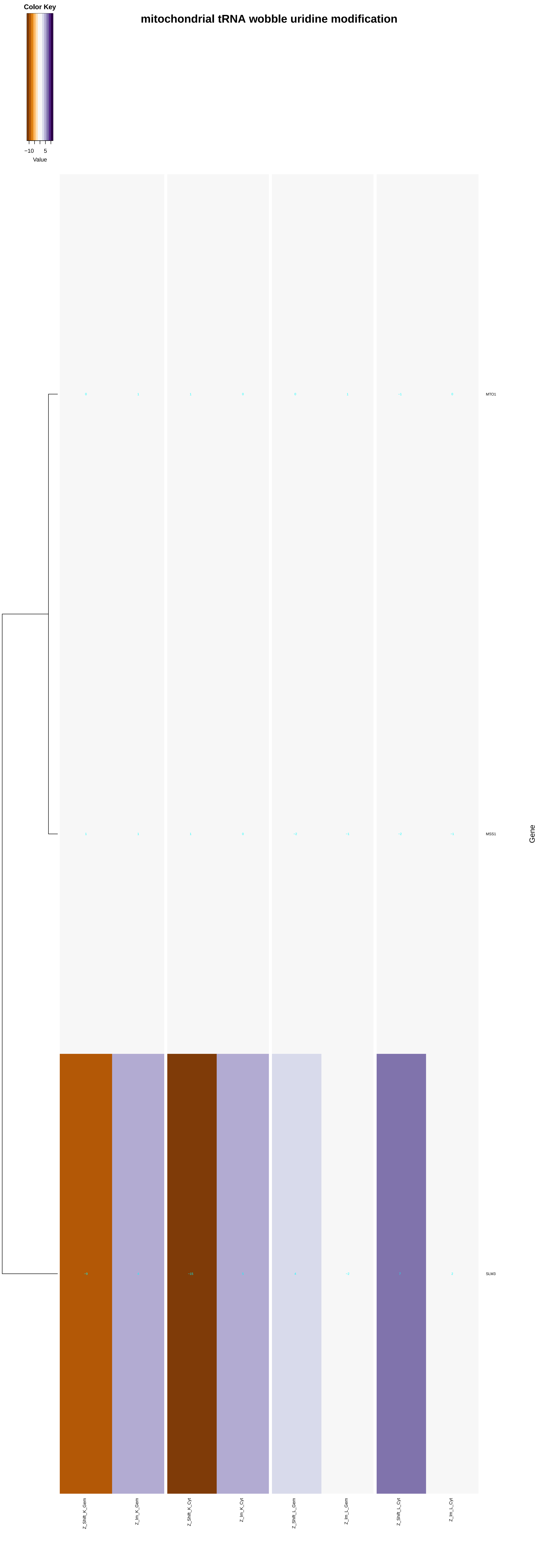
mitochondrial tRNA wobble uridine modification
Color Key
−10
5
Value
MTO1
MSS1
SLM3
Gene
Z_Shift_K_Gem
Z_Im_K_Gem
Z_Shift_K_Cyt
Z_Im_K_Cyt
Z_Shift_L_Gem
Z_Im_L_Gem
Z_Shift_L_Cyt
Z_Im_L_Cyt
